# Supplementary material for: Impact of obesity on life expectancy among different European countries: secondary analysis of population-level data over the 1975–2012 period
Source: BMJ Open. 2019 Jul 31;9(7):e028086. doi: 10.1136/bmjopen-2018-028086 (PMC6678519; doi:10.1136/bmjopen-2018-028086)
Supplement: Supplementary data [file bmjopen-2018-028086supp001.pdf]

Impact of obesity on life expectancy among different European countries, 1975-2012

Nikoletta Vidra[1], Sergi Trias-Llimós[1], Fanny Janssen[1,2]

[1] Population Research Centre, Faculty of Spatial Sciences, University of Groningen, Landleven 1, 9747 AD Groningen, The Netherlands.

[2] Netherlands Interdisciplinary Demographic Institute, Lange Houtstraat 19, 2511 CV The Hague, The Netherlands

Supplementary material

Table S1: Age-and sex-specific RRs of dying from obesity from the meta-review from Lobstein et al. (2010)

**Age**	**RR***	
	Men	***Women***
**<50**	1.55	1.5
**50-59**	1.539	1.49
**60-69**	1.5225	1.475
**70+**	1.495	1.45

*Reference group for the RRs: normal weight (18.5≥BMI≤24.9 kg/m^2)

Table S2: Potential gains in life expectancy at birth (PGLE) if obesity-attributable mortality was eliminated, in 26 European countries (differentiating Western and Central Eastern Europe) and the USA, in 1975 and 2012, 18-100 years

	PGLE 1975		PGLE 2012	
**Country**	***Men***	***Women***	***Men***	***Women***
Central Eastern Europe (CEE)				
Belarus	0.41	0.79	1.41	1.19
Czech Republic	0.70	0.98	1.39	1.03
Estonia	0.55	1.00	1.37	1.04
Hungary	0.64	0.86	1.52	1.04
Latvia	0.58	1.00	1.48	1.18
Lithuania	0.54	1.05	1.67	1.31
Poland	0.57	0.93	1.48	1.19
Russian Federation	0.51	1.26	1.53	1.54
Slovakia	0.43	0.62	1.31	0.96
Ukraine	0.47	0.95	1.25	1.16
Average CEE	0.54	0.94	1.44	1.16
Western Europe				
Austria	0.40	0.39	1.03	0.73
Belgium	0.55	0.61	1.17	0.97
Denmark	0.42	0.46	1.04	0.79
France	0.49	0.53	1.18	0.84
Finland	0.45	0.40	1.19	0.90
Ireland	0.38	0.31	1.21	1.01
Iceland	0.49	0.48	0.97	0.80
Italy	0.44	0.60	1.06	0.93
Luxembourg	0.47	0.42	1.19	0.79
Netherlands	0.29	0.39	0.86	0.88
Norway	0.34	0.39	1.07	0.91
Portugal	0.24	0.41	1.01	0.81
Spain	0.42	0.66	1.22	1.05
Sweden	0.42	0.43	0.91	0.76
Switzerland	0.35	0.35	0.93	0.66
United Kingdom	0.53	0.50	1.27	1.09
**Average Western Europe**	0.41	0.48	1.08	0.86
USA	0.69	0.72	1.73	1.44
**Average European countries**	0.46	0.64	1.22	0.98
**Average all countries**	0.47	0.64	1.23	1.00

Figure S1: Age-standardised obesity-attributable mortality fractions in 26 European countries*, grouped by 5 regions and USA, 1975-2014, 18-100 years

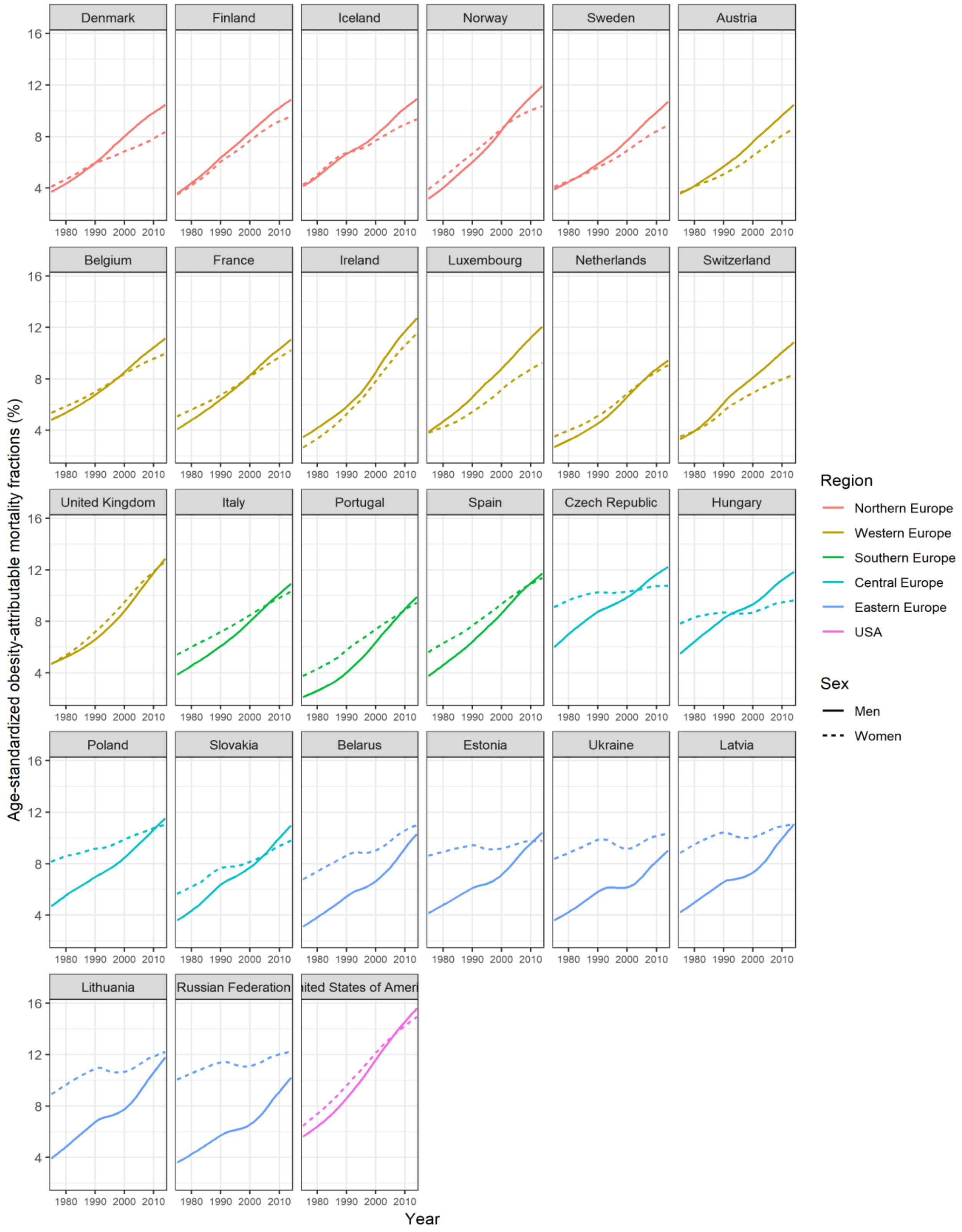

* Countries within the same region are presented with the same colour

Figure S2: Potential gains in life expectancy at birth (PGLE) if obesity-attributable mortality was eliminated, in 26 European countries*, grouped by 5 regions and USA, 1975-2012, 18-100 years

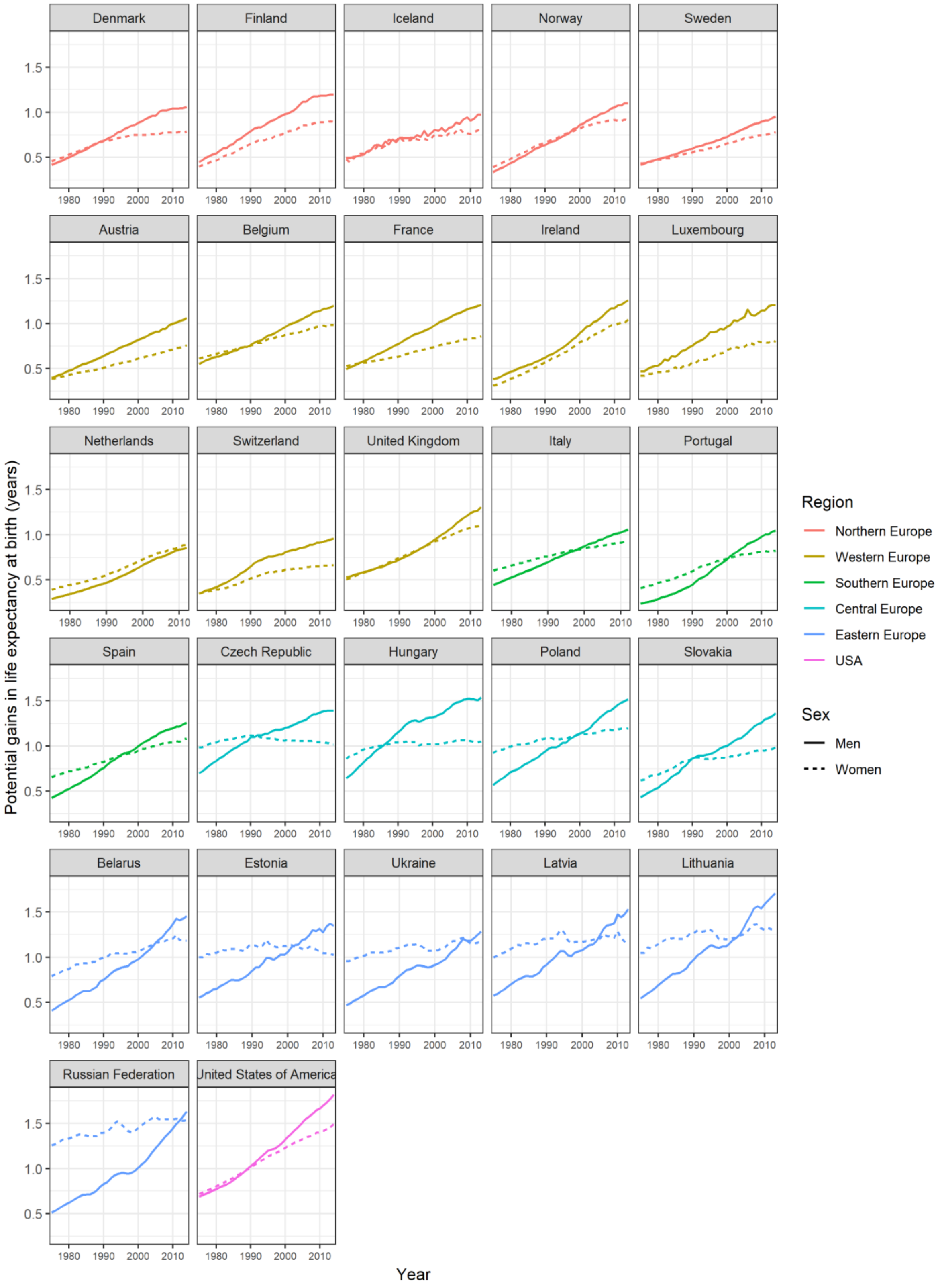

* Countries within the same region are presented with the same colour

Table S3: Age-standardised obesity prevalence and 95% confidence intervals, in 26 European countries (differentiating Western and Central Eastern Europe) and USA, 18-100 years in 2012.

	Age-standardised (stand.) obesity prevalence (%)			
**Country**	**Age-stand. Men**	**95% confidence intervals**	**Age-stand. Women**	**95% confidence intervals**
Central Eastern Europe				
Belarus	20.4	13.6; 28.5	25.4	17.7; 34.1
Czech Republic	25.6	18.9; 33.4	25.2	18.3; 33.0
Estonia	21.0	16.0; 26.8	22.7	17.4; 28.8
Hungary	24.6	17.9;32.0	22.1	15.4; 29.8
Latvia	22.2	15.2; 30.2	25.8	18.4; 34.5
Lithuania	23.8	16.9; 31.8	28.7	21.2; 37.1
Poland	23.6	18.0; 29.7	25.7	19.5; 32.5
Russian Federation	20.4	14.8; 26.8	29.1	22.6; 36.2
Slovakia	22.2	15.7; 29.6	22.2	15.8; 29.6
Ukraine	17.7	11.2; 25.7	23.9	16.3; 32.5
Western Europe				
Austria	21.1	14.7; 28.2	19.1	13.4; 25.5
Belgium	22.9	17.3; 29.1	22.7	17.1; 28.9
Denmark	21.3	15.5; 27.9	18.4	13.1; 24.5
France	22.6	16.3; 29.6	23.1	16.9; 29.9
Finland	22.3	17.1; 28.2	21.7	16.7; 27.3
Ireland	26.2	19.4; 33.8	26.0	19.5; 33.2
Iceland	22.3	15.6; 29.8	21.0	14.7; 28.3
Italy	22.3	17.0; 28.1	23.5	18.1; 29.6
Luxembourg	24.9	17.6; 32.9	20.7	14.3; 28.0
Netherlands	18.9	13.8; 24.5	20.3	15.3; 25.8
Norway	24.5	18.5; 31.2	23.7	18.0; 30.2
Portugal	19.6	13.8; 26.4	21.1	15.0; 28.1
Spain	24.2	18.5; 30.5	26.4	20.3; 32.8
Sweden	21.6	16.3; 27.4	19.8	14.7; 25.6
Switzerland	22.0	16.3; 28.3	18.6	13.1; 24.8
United Kingdom	26.5	22.3; 31.2	29.1	24.8; 33.6
USA	33.4	27.5; 39.5	35.5	29.7; 41.5

Table S4: Potential gains in life expectancy at age 50 (PGLE) if obesity-attributable mortality was eliminated, own estimates and those by Preston et al.2011, in the same countries studied, in 2006

Country	PGLE e50 2006, own estimates	PGLE e50 2006, Preston's estimates	Difference
Men			
Austria	0.81	1.00	-0.19
Belgium	0.95	0.98	-0.03
Czech Republic	1.14	1.34	-0.20
Denmark	0.88	0.82	0.06
France	0.94	0.99	-0.05
Italy	0.88	0.90	-0.02
Netherlands	0.69	0.73	-0.04
Poland	1.14	1.37	-0.23
Spain	1.02	1.15	-0.13
Sweden	0.75	0.72	0.03
Switzerland	0.77	0.79	-0.02
United Kingdom	0.99	1.34	-0.35
USA	1.29	1.85	-0.56
Women			
Austria	0.62	0.71	-0.09
Belgium	0.86	0.73	0.13
Czech Republic	0.87	1.01	-0.14
Denmark	0.71	0.62	0.09
France	0.72	0.52	0.2
Italy	0.84	0.57	0.27
Netherlands	0.76	0.69	0.07
Poland	1.08	1.19	-0.11
Spain	0.95	0.87	0.08
Sweden	0.67	0.63	0.04
Switzerland	0.59	0.50	0.09
United Kingdom	0.94	1.23	-0.29
USA	1.18	1.28	-0.10

Figure S3: Age-standardised obesity prevalence in 26 European countries*, grouped by 5 regions and USA, 1975-2012, 18-100 years

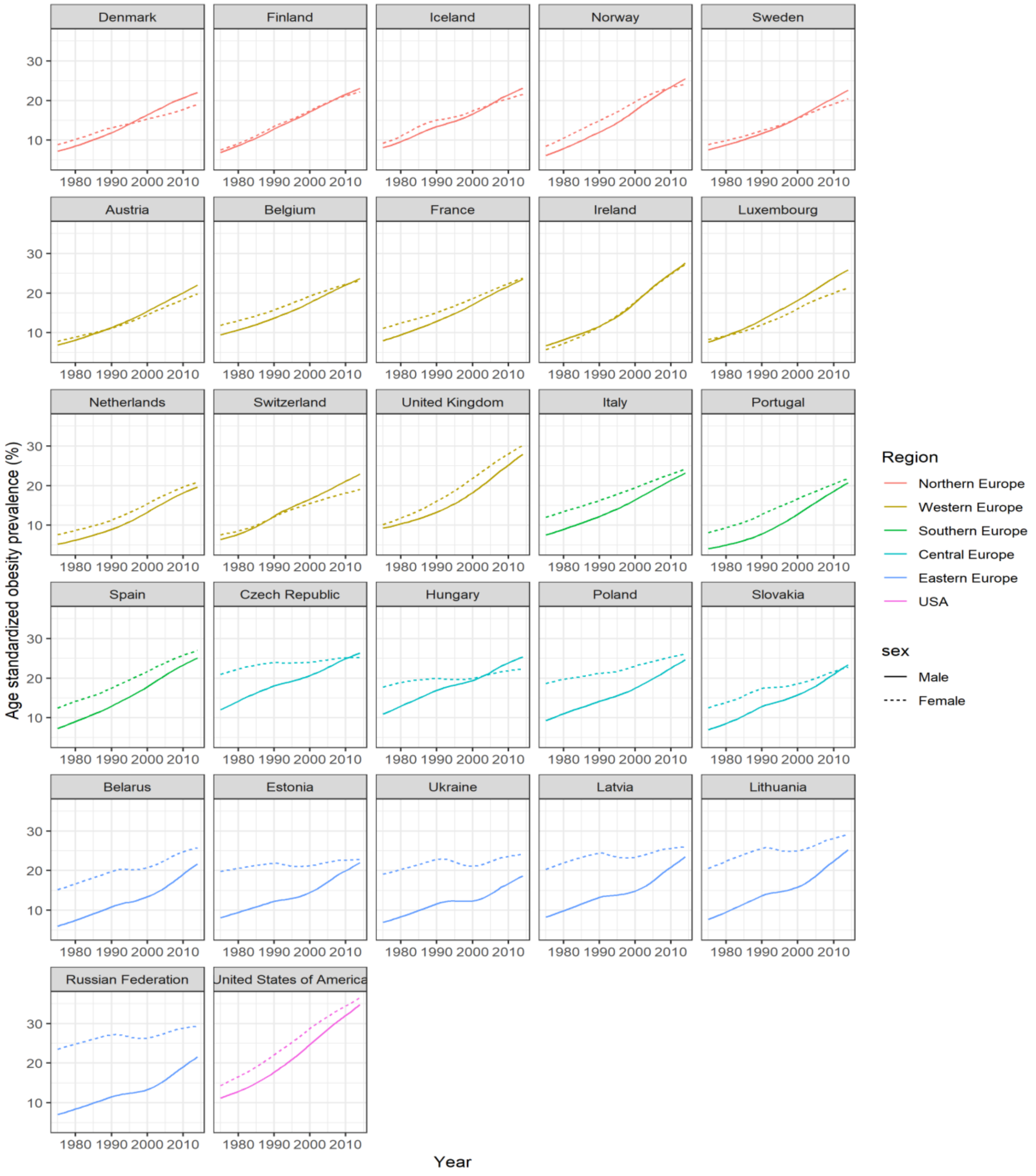

* Countries within the same region are presented with the same colour
