# Supplementary material for: Impact of obesity on life expectancy among different European countries: secondary analysis of population-level data over the 1975–2012 period
Source: BMJ Open. 2019 Jul 31;9(7):e028086. doi: 10.1136/bmjopen-2018-028086 (PMC6678519; doi:10.1136/bmjopen-2018-028086)
Supplement: Author's manuscript [file bmjopen-2018-028086.draft_revisions.pdf]

BMJ Open is committed to open peer review. As part of this commitment we make the peer review history of every article we publish publicly available.

When an article is published we post the peer reviewers' comments and the authors' responses online. We also post the versions of the paper that were used during peer review. These are the versions that the peer review comments apply to.

The versions of the paper that follow are the versions that were submitted during the peer review process. They are not the versions of record or the final published versions. They should not be cited or distributed as the published version of this manuscript.

BMJ Open is an open access journal and the full, final, typeset and author-corrected version of record of the manuscript is available on our site with no access controls, subscription charges or pay-per-view fees (http://bmjopen.bmj.com).

If you have any questions on BMJ Open's open peer review process please email info.bmjopen@bmj.com

BMJ Open

Impact of obesity on life expectancy among different European countries, 1975-2012

Journal:	*BMJ Open*
Manuscript ID	bmjopen-2018-028086
Article Type:	Research
Date Submitted by the Author:	22-Nov-2018
Complete List of Authors:	Vidra, Nikoletta; Population Research Centre, Faculty of Spatial Sciences, Demography<br>Trias-Llimós, Sergi; University of Groningen, Population Research Centre<br>Janssen, Fanny; University of Groningen, Population Research Centre; Nederlands Interdisciplinair Demografisch Instituut
Keywords:	EPIDEMIOLOGY, NUTRITION & DIETETICS, PUBLIC HEALTH

SCHOLARONE™
Manuscripts

Impact of obesity on life expectancy among different European countries, 1975-2012

Nikoletta Vidra[1], Sergi Trias-Llimós[1], Fanny Janssen[1,2]

[1] Population Research Centre, Faculty of Spatial Sciences, University of Groningen, Landleven 1, 9747 AD Groningen, The Netherlands.

[2] Netherlands Interdisciplinary Demographic Institute, Lange Houtstraat 19, 2511 CV The Hague, The Netherlands.

Corresponding author: Nikoletta Vidra, Population Research Centre, Faculty of Spatial Sciences, University of Groningen, PO Box 800, 9700 AV Groningen, The Netherlands. Tel: 0031 (0) 50 363 86 52; e-mail: n.vidra@rug.nl

Word count: 3053 words

For peer review only - http://bmjopen.bmj.com/site/about/guidelines.xhtml

Abstract

Objective: This study assesses the impact of obesity on life expectancy for 26 European national populations and the USA over the 1975-2012 period.

Design: Secondary analysis of population-level obesity and mortality data.

Setting: European countries, namely Austria, Belarus, Belgium, the Czech Republic, Denmark, Estonia, Finland, France, Hungary, Iceland, Ireland, Italy, Latvia, Lithuania, Luxembourg, the Netherlands, Norway, Poland, Portugal, the Russian Federation, Slovakia, Spain, Sweden, Switzerland, Ukraine, the United Kingdom (UK); and the USA.

Participants: National populations aged 18–100 years, by sex.

Measurements: Using data by age and sex, we calculated obesity-attributable mortality by multiplying all-cause mortality (Human Mortality Database) with obesity-attributable mortality fractions (OAMFs). OAMFs were obtained by applying the partially adjusted method to obesity prevalence data (NCD Risk Factor Collaboration) and European Relative Risks (RRs) (DYNAMO). We estimated potential gains in life expectancy at birth (PGLE) by eliminating obesity-attributable mortality from all-cause mortality using associated single-decrement life tables.

Results: In the 26 European countries in 2012, PGLE due to obesity ranged from 0.86 to 1.67 years among men, and from 0.66 to 1.54 years among women. In all countries, PGLE increased over time, with an average annual increase of 2.68% among men and 1.33% and among women. Among women in Denmark, Switzerland, and Central and Eastern European countries, the increase in PGLE levelled off after 1995. Without obesity, the average increase

For peer review only - http://bmjopen.bmj.com/site/about/guidelines.xhtml

in life expectancy between 1975 and 2012 would have been 0.78 years higher among men and 0.30 years higher among women.

Conclusions: Obesity was proven to have an impact on both life expectancy levels and trends in Europe. The differences found in this impact between countries and the sexes can be linked to contextual factors, as well as to differences in people's ability and capacity to adopt healthier lifestyles.

Keywords: Obesity, life expectancy, Europe, USA

Article Summary

Strengths and limitations of the study

- This is the first study to assess the impact of obesity on life expectancy at birth over time, and we do so here for 26 European countries and the USA.
- We used recent long-term comparable data on obesity prevalence based on population-based measurement studies, and European relative risks of dying from obesity by age and sex from a recent meta-analysis.
- Because of remaining data limitations regarding prevalence and relative risks, we had to use a fairly simple – albeit common applied - methodology to estimate obesity-attributable mortality.

For peer review only - http://bmjopen.bmj.com/site/about/guidelines.xhtml

Introduction

Obesity is a global epidemic (1), with Europe currently ranking second worldwide after the USA (2). Over the last 20 years obesity prevalence has increased threefold in Europe, (3) although not uniformly across countries (4). Estimates for 2014 indicate that obesity varied threefold across European countries, ranging from a low of 9% in Romania to a high of 26% in Malta (5). Obesity constitutes a serious health burden at the individual and population levels because it is associated with an increased risk of morbidity (6), and mortality (7). However, the potential impact of the increase in obesity on life expectancy trends remains largely unknown (8).

The few existing studies that assessed the impact of obesity on life expectancy at the population level provided estimates at one specific point in time only (9, 10). Olshansky et al. found that if obesity was eliminated, life expectancy at birth (e0) in the USA in 2000 would be 0.21 to 1.08 years higher, depending on gender and ethnicity (9). Preston et al. (10) estimated for 16 low-mortality countries in 2006 that the reduction in life expectancy at age 50 (e50) due to obesity was greatest in the USA, at more than 1.5 years; and ranged from 0.50 to 1.19 years for women and from 0.72 to 1.37 years for men in European countries.

Gaining insight into the impact of obesity on trends in life expectancy is especially relevant (11) given the marked differences in life expectancy trends across Europe (12). In Western European countries, e0 has been increasing steadily, and has risen six to eight years since 1970. But in Central and Eastern Europe (CEE), e0 stagnated or even declined between the 1970s and the 1980s, and did not start increasing again until the 1990s. There are also marked differences in e0 trends between individual European countries (12).

For peer review only - http://bmjopen.bmj.com/site/about/guidelines.xhtml

For peer review only

In light of these important differences between European countries in both obesity prevalence and life expectancy over time, our aim is to assess the impact of obesity on long-term trends in life expectancy across a wide range of European countries.

Data and Methods

Setting

We studied the impact of obesity on life expectancy by sex over the 1975-2012 period in 26 European countries: Austria, Belarus, Belgium, the Czech Republic, Denmark, Estonia, Finland, France, Hungary, Iceland, Ireland, Italy, Latvia, Lithuania, Luxembourg, the Netherlands, Norway, Poland, Portugal, the Russian Federation, Slovakia, Spain, Sweden, Switzerland, Ukraine, the United Kingdom (UK); and the USA as a comparison country (10).

Data

Long-term comparable obesity prevalence data (BMI≥30kg/m2) by country, sex, age (18-19, 20-24, …, 85+), and year (1975-2012), based on population-based measurement studies, were obtained from the NCD Risk Factor Collaboration study (13). These validated data comprise the available measured height and weight data, supplemented with estimates based on information from other years and related countries from a Bayesian hierarchical model (13).

The age- (<50, 50-59, 60-69 and ≥70 years) and sex-specific relative risks (RRs) of dying from obesity (see Appendix Table S1) came from a review of studies mainly conducted in Western Europe and the USA (14). These age- and sex-specific RRs were largely in line with the overall European RR of 1.64 recently estimated by the Global BMI Mortality Collaboration (15).

For peer review only - http://bmjopen.bmj.com/site/about/guidelines.xhtml

All-cause mortality numbers and exposure population data by single year of age, sex, and year were obtained from the Human Mortality Database (16).

Patient and Public Involvement

No patients were involved in this study.

Methods

We performed our analyses separately by country and sex, based on data by single year of age (18-100). The obesity prevalence data were turned into single-age prevalence (18-100) by applying Loess smoothing (17). The RRs were turned into single-year RRs (18- 100) using linear regression.

To estimate the obesity-attributable mortality fraction (OAMF) – i.e., the share of all-cause mortality due to obesity – we used the Rockhill formula to estimate OAMFs by age (a) and sex (s) (18).

$$OAMF_{a,s} = \frac{P_{a,s} \cdot (RR_{a,s} - 1)}{1 + (P_{a,s} \cdot (RR_{a,s} - 1))} \text{(Equation 1)}$$

where *P* is the obesity prevalence. We then weighted the $OAMF_{a,s}$ with the corresponding number of deaths.

For the estimation of the impact of obesity on life expectancy (see 2.3.2) we needed age-and sex-specific (non-) obesity-attributable mortality rates. These were obtained by multiplying $OAMF_{a,s}$ and [1- $OAMF_{a,s}$], respectively, with age- and sex-specific all-cause mortality rates.

To ensure comparability across countries, over time, and between men and women, we applied direct age- and sex-standardisation (19) to obesity prevalence, obesity-

For peer review only - http://bmjopen.bmj.com/site/about/guidelines.xhtml

attributable mortality fractions, and obesity-attributable mortality rates, using the European population of 2011 (20) as the standard.

To assess the impact of adult obesity on e_0, we calculated for each country the potential gain in life expectancy (PGLE) if obesity-attributable mortality were eliminated, by calendar year and sex. First, we calculated e_0 by applying standard life table techniques to age-specific all-cause mortality rates (0-100) (19). Second, we applied associated single-decrement life tables (ASDLT) (19) to age- and sex-specific non-obesity-attributable mortality rates (0-100) to obtain e_0 if obesity-attributable mortality were eliminated. The PGLE represents the difference between the e_0 based on the ASDLT and the original e_0.

To summarise the changes in PGLE across countries, we estimated the average annual changes in PGLE (in %):

$$Average\ annual\ changes\ in\ PGLE\ (\%) = \frac{\sum_{t=1976}^{2012} \frac{(PGLE_t - PGLE_{t-1})}{PGLE_{t-1}}}{2012 - 1975} 100$$

To assess the impact of obesity on time trends in e_0 between 1975 and 2012, we subtracted the observed change in e_0 from the change in e_0 without obesity. The change in e_0 without obesity was obtained by using the e_0 values from the associated single-decrement life tables applied to non-obesity-attributable mortality for 1975 and 2012.

Results

For the 26 European countries, the age-standardised obesity-attributable mortality fraction (OAMF) was, on average, 11% among men and 10% among women in 2012. For the USA, these estimates were substantially higher; i.e., 15% and 14%, respectively. The average

For peer review only - http://bmjopen.bmj.com/site/about/guidelines.xhtml

OAMF levels were higher in Northern, Western, and Southern Europe combined (hereafter, Western Europe) than in CEE among men, while the opposite was the case among women.

OAMFs were increasing over time for all countries and both sexes, although not to the same extent (see Figure 1, Figure S1). In Western Europe, OAMFs generally increased over the 1975-2012 period, and at a faster pace among men. In CEE, by contrast, OAMFs clearly stagnated, and even declined between 1990 and 2000. The overall increase in OAMFs was greatest in the USA, Ireland, Norway (men), and the UK (women).

Figure 1: Age-standardised obesity-attributable mortality fractions in 26 European countries (by 5 regions) and USA, 1975-2014, 18-100 years

<approximately here>

In the 26 European countries in 2012, estimates of potential gains in life expectancy at birth (PGLE) if obesity was eliminated ranged from 0.86 to 1.67 years among men (1.22 on average) and from 0.66 to 1.54 years (0.98 on average) among women (Figure 2; Figure S2 and Table S2 Appendix). Among men in the USA, the PGLE estimate was, at 1.73 years, slightly higher than the highest estimate in Europe; and among women in the USA, the PGLE estimate was, at 1.44 years, the second-highest after the estimate for Russia. The average PGLE estimate was 1.08 among men and 0.86 among women in Western Europe, and was 1.44 among men and 1.16 among women in CEE (see Appendix Table S2).

For peer review only - http://bmjopen.bmj.com/site/about/guidelines.xhtml

Figure 2: Potential gains in life expectancy at birth (PGLE) if obesity-attributable mortality was eliminated, in 26 European countries (differentiating Western and Central Eastern Europe) and the USA, 1975-2012

<approximately here>

Overall, from 1975 to 2012, PGLE due to obesity increased in all of the countries (Figure 2, Figure S2, Figure 3, 4). The increase was greater among men (average annual increase of 2.68%) than among women (average annual increase of 1.33%), was largest among men in Portugal and Belarus and among women in Portugal, and was substantial among men and women in Norway (Figure 3, 4). While there was a general increase in PGLE due to obesity, this trend stagnated among women in CEE from around 1990 onwards, and levelled off after 1995 among women in Denmark and Switzerland.

Figure 3: Average annual increase (%) in potential gains in life expectancy due to obesity in 26 European countries and the USA between 1975-2012, in men

<approximately here>

Figure 4: Average annual increase (%) in potential gains in life expectancy due to obesity in 26 European countries and the USA between 1975-2012, in women

<approximately here>

Table 1 shows the impact of obesity on time trends in life expectancy at birth (e_0). Overall, the average increase in e_0 between 1975 and 2012 was 7.26 years for men and 6.28 years for women in the 26 European countries. Without obesity, the average increase in e_0 would have been 8.04 years for men and 6.58 years for women; or 0.78 and 0.30 years higher, respectively. Among men, obesity had the greatest impact on e_0 trends in Lithuania

For peer review only - http://bmjopen.bmj.com/site/about/guidelines.xhtml

and the USA (more than one year), and the smallest impact in Iceland and Sweden (0.5 years). Among women, obesity had the greatest impact on e_0 trends in the USA and Ireland (0.7 years) and the smallest impact in Estonia and the Czech Republic (less than 0.1 year).

Table 1: Impact of obesity on trends in life expectancy at birth (e_0) in 26 European countries and USA 1975-2012, by sex

Country	Change in e_0 with obesity 2012-1975 (years)		Change in e_0 without obesity 2012-1975 (years)		Effect of obesity on e_0 change 2012-1975 (years)	
	Men	*Women*	*Men*	*Women*	*Men*	*Women*
Austria	10.62	8.61	11.25	8.95	0.63	0.34
Belarus	-0.55	1.43	0.46	1.83	1.00	0.40
Belgium	8.85	7.63	9.46	7.99	0.61	0.36
Czech Republic	7.97	6.98	8.66	7.03	0.69	0.05
Denmark	6.78	5.03	7.40	5.36	0.63	0.33
Estonia	6.43	6.42	7.26	6.46	0.82	0.04
France	9.49	7.99	10.17	8.30	0.68	0.31
Finland	10.07	7.26	10.82	7.75	0.74	0.50
Hungary	5.29	6.18	6.17	6.36	0.87	0.18
Iceland	9.02	5.19	9.51	5.51	0.48	0.32
Ireland	9.40	8.40	10.22	9.10	0.83	0.69
Italy	10.19	8.56	10.81	8.89	0.62	0.33
Latvia	4.91	4.53	5.82	4.70	0.90	0.18
Lithuania	2.01	3.80	3.14	4.06	1.13	0.26
Luxembourg	11.78	9.27	12.50	9.65	0.72	0.37
Netherlands	7.68	5.10	8.26	5.6	0.56	0.49
Norway	7.70	5.33	8.42	5.86	0.74	0.51
Poland	5.90	6.74	6.81	7.00	0.91	0.27
Portugal	12.14	10.87	12.91	11.26	0.77	0.40
Russian Federation	2.05	2.62	3.06	2.89	1.02	0.28
Slovakia	5.65	5.82	6.52	6.16	0.88	0.34
Spain	8.82	8.75	9.62	9.14	0.79	0.39
Sweden	7.69	5.59	8.18	5.93	0.49	0.33
Switzerland	8.98	6.63	9.55	6.93	0.58	0.30
Ukraine	0.48	1.73	1.26	1.94	0.78	0.21
United Kingdom	9.46	6.96	10.20	7.55	0.74	0.59
USA	7.86	4.89	8.90	5.61	1.04	0.71
**Average CEE countries**	4.01	4.63	4.92	4.84	0.90	0.22

For peer review only - http://bmjopen.bmj.com/site/about/guidelines.xhtml

**Average Western countries**	9.10	7.09	9.76	7.50	0.66	0.41

Discussion

Summary of results

In the 26 European countries studied, the share of mortality due to obesity in 2012 was, on average, 11% among men and 10% among women. PGLE due to obesity in 2012 ranged from 0.86 to 1.73 years among men, and from 0.66 to 1.54 years among women. Overall, PGLE increased between 1975 and 2012, albeit more quickly among men (average annual increase: 2.68%) than among women (1.33%). Among women in Denmark, Switzerland, and the CEE countries the increase in PGLE levelled off after 1995. Without obesity, the average increase in e0 between 1975 and 2012 would have been 0.78 years higher among men and 0.30 years higher among women.

Evaluation of data and methods

Using the recent advances in obesity data, it is now possible to study the impact of obesity on life expectancy for a large number of countries and a long period of time. Two methodological issues warrant our attention, however.

First, in calculating the share of mortality due to obesity (OAMF), which also forms the basis for our PGLE calculations, we were hindered by limitations in the available prevalence and RRs data, which also affected the method used. As has previously been documented, OAMF estimates are sensitive to the data and the methods used (21).

In selecting obesity prevalence data, we used the longest validated time series based

For peer review only

For peer review only - http://bmjopen.bmj.com/site/about/guidelines.xhtml

on population-based measurement studies that are suitable for studying the impact of obesity on long-term life expectancy trends across Europe (13). For those countries with less available obesity data – especially CEE countries – a portion of the data we used were modelled, and these should be treated with some caution (13).

Because age- and sex-specific RRs of mortality associated with obesity are not available by country and year, we applied to all of the countries studied time-constant age- and sex-specific RRs from Western European and US populations that are largely suitable for our setting. However, literature for the USA has demonstrated that RRs have been changing over time, pointing to both a decline (22-24) and an increase (25). Before implementing time-variant European RRs, more information on their direction is required. Similarly, comparable country-specific RRs are urgently needed.

Based on the available data, only a fairly simple – albeit common applied –the partially adjusted method could be applied (21) to estimate the OAMFs. The application of a more advanced methodology (21) could have affected the OAMFs and thus the PGLE levels, but less the trends (26).

Second, besides being the result of the OAMFs, the PGLE estimates can also be affected by all-cause mortality levels and trends as age- and sex-specific all-cause mortality rates are used to estimate PGLE. Since all-cause mortality fluctuated greatly in CEE in the analysed period (12), short-term variations in PGLE in CEE countries should be treated with more caution.

Explanation of results

In 2012, the PGLE due to obesity were, on average, 1.22 years for men and 0.98 years for women in the 26 European countries, and 1.73 years for men and 1.43 years for women in the USA. A comparison of our 2006 e50 estimates with those of Preston et al. (10) for the

For peer review only - http://bmjopen.bmj.com/site/about/guidelines.xhtml

same countries uncovered only small differences, except among men in the USA (our estimate was 0.56 years lower) and women in the UK (our estimate was 0.29 years lower) (see Appendix Table S3). Given that approximately the same methodology was used to estimate the OAMFs, the observed differences are most likely due to the use of different obesity prevalence and RRs data. Preston used prevalence data from national representative surveys and RRs from the Prospective Studies collaboration (14).

To further evaluate our observed PGLE levels, we compared them with own PGLE estimates for smoking and alcohol (27). Our PGLE estimates for smoking were 2.38 years for men and 1.00 year for women in Western Europe, and 3.82 years for men and 0.67 years for women in CEE. Our PGLE estimates for alcohol were 0.90 years for men and 0.44 years for women in Western Europe, and 2.15 years for men and 1.00 year for women in CEE (27). Thus, obesity's impact on life expectancy lies between that of smoking and alcohol, and can be considered significant.

In our study, we found that PGLE due to obesity was increasing, but that this trend differed across countries and between the sexes. This overall trend can be explained by the general increase in obesity prevalence in European countries (see Appendix Figure S3) (13) and the resulting growth in the burden of obesity (3), which is also reflected in the OAMFs (Figure 1, Figure S1) in these countries.

At the same time, parts of the observed variation in the increase in PGLE estimates across the USA, Western Europe, and CEE and between the sexes reflect differences in the onset, the development, and the impact of the obesity epidemic in these countries and in men and women. Across the countries studied, the absolute increase in PGLE was largest among women and second-largest among men in the USA. This pattern is in line with

For peer review only - http://bmjopen.bmj.com/site/about/guidelines.xhtml

evidence showing that between 1980 and 2008, obesity increased much more in the USA than in Europe (1, 28). This rapid progression of the obesity epidemic in the USA and its large impact on life expectancy has been attributed to an increasingly obesogenic environment caused by factors such as changes in food preparation and processes that promote the consumption of calorically dense foods, and a pronounced decrease in physical activity levels (29). The obesity epidemic has progressed more slowly in Western Europe than in the USA (1, 13). However, obesity levels in countries like the UK and Ireland are rapidly approaching those in the USA (30), as our PGLE estimates also show.

In the CEE countries, the PGLE trends track the evolution of the obesity epidemic in that region (see Appendix Figure S3). Obesity levels have been higher in CEE than in Western Europe since 1980 (31, 32), which suggests that the epidemic started earlier in CEE. As a result of this earlier onset, the impact of obesity (as expressed in terms of OAMF and PGLE) in the 1970s and 1980s was at times even greater in CEE than in the USA, especially among women. While there are many potential explanations for this early onset of the obesity epidemic in CEE, the available data indicate that the main factors were the relatively high total energy supply and energy intake in CEE in those years (33).

The overall progress of the obesity epidemic was lower in CEE than in Western Europe, and the increase was not constant (1). Indeed, in CEE, increases in obesity prevalence (1, 34), OAMFs, and PGLE stagnated in the 1980-2008 period, more pronounced in the 1990s (1, 34). This pattern could be explained by the decrease in energy supplies at the beginning of the 1990s in CEE (31) resulting from the dramatic economic and political changes in those countries (1, 31, 34). Among CEE women, the increase in obesity starting in the 1990s was smaller than it was in the previous period, and was smaller than it was among

For peer review only - http://bmjopen.bmj.com/site/about/guidelines.xhtml
For peer review only

CEE men. The lower risk of obesity observed among women than among men with low socioeconomic status (SES) in low-income countries (35) may explain this difference.

In Western Europe, a stagnation in PGLE levels was observed among women in Denmark and Switzerland after 1995. This finding seems to be in line with studies reporting a levelling-off of mean BMI since the 1990s (36); and in specific sub-populations, such as adults with high SES in regions within Switzerland, Italy, France, and Finland (31). Although dietary and physical activity information is spreading equally across socioeconomic groups, those with higher SES have a greater ability and capacity to adopt a healthier dietary and physical activity pattern (37). In addition, it appears that higher SES women in particular are more health-conscious, have healthier food habits, and are more prone to follow nutritional recommendations (38) as they are under greater social pressure to be thin (39). Similarly, countries with higher income levels and lower levels of inequality (40), like Switzerland and Denmark, tend to have lower obesity levels, especially among women.

When we considered the impact of obesity on life expectancy in the 26 European countries, we found that without obesity, the increase in e0 between 1975 and 2012 would have been, on average, 0.78 years higher among men and 0.30 years higher among women. These figures account for approximately 10% of the average change in e0 between 1975 and 2012 among men, and 5% among women. It is therefore clear that the impact of obesity on changes in e0 should not be ignored. Moreover, the impact of obesity on life expectancy trends is likely to increase, given that this impact is already substantially greater in the USA (13% among men and 15% among women), and that obesity prevalence is still increasing rapidly in most European countries (see Appendix Figure S3).

Conclusion and implications

For peer review only - http://bmjopen.bmj.com/site/about/guidelines.xhtml

Obesity was proven to have an impact on both life expectancy levels and trends in Europe. The observed differences in the increase in the impact of obesity across countries and between the sexes reflect differences in the onset and the progression of the obesity epidemic, and can be linked to contextual factors (economic conditions, obesogenic environment, energy supplies), as well as to differences in people's ability and capacity to adopt healthier lifestyles.

It is likely that in the future obesity will have a larger impact on mortality and life expectancy in Europe, as obesity continues to increase in the majority of countries. It is therefore crucial that effective public health initiatives are undertaken to tackle the obesity epidemic and its effects on public health. Such initiatives should address the multifactorial and complex obesity aetiology; the clear differences between countries and the sexes; as well as the factors underlying these differences, such as contextual factors and differences in individuals' ability and capacity to adopt healthier lifestyles.

For peer review only

For peer review only - http://bmjopen.bmj.com/site/about/guidelines.xhtml

Funding

This work was supported by the Netherlands Organisation for Scientific Research (NWO) in connection with the research programme “Smoking, alcohol, and obesity, ingredients for improved and robust mortality projections, grant no. 452-13-001; see www.futuremortality.com.

Conflict of Interest

None declared.

Author contribution

N.Vidra contributed to the conception and design of the work; to the acquisition, analysis, and interpretation of data for the work; drafted and revised the work critically for important intellectual content; approved the final version of the work to be published and agrees to be accountable for all aspects of the work in ensuring that questions related to the accuracy or integrity of any part of the work are appropriately investigated and resolved.

S. Trias-Llimós contributed to the analysis, and interpretation of data for the work; revised it critically for important intellectual content; approved the final version of the work to be

For peer review only

published and agrees to be accountable for all aspects of the work in ensuring that questions related to the accuracy or integrity of any part of the work are appropriately investigated and resolved.

F. Janssen contributed to the conception and design of the work; to the interpretation of data for the work; revised the work critically for important intellectual content; approved the final version of the work to be published and agrees to be accountable for all aspects of the work in ensuring that questions related to the accuracy or integrity of any part of the work are appropriately investigated and resolved.

Data statement

Obesity prevalence data can be obtained from NCD Risk Factor Collaboration (NCD-RisC) (www.ncdrisc.org).

All-cause mortality and exposure population data can be obtained from the Human Mortality Database (http://www.mortality.org).

Acknowledgements

The authors are grateful to Professor Majid Ezatti and Dr James Bentham (Faculty of Medicine, School of Public Health at Imperial College London); Dr Gretchen A. Stevens (World Health Organization); and the NCD Risk Factor Collaboration (NCD-RisC) (www.ncdrisc.org) for sharing the obesity data.

This work is financed by the Netherlands Organisation for Scientific Research (NWO) as part of the research programme “Smoking, alcohol, and obesity, ingredients for improved and robust mortality projections, grant no. 452-13-001. See www.futuremortality.com

For peer review only - http://bmjopen.bmj.com/site/about/guidelines.xhtml

References

1. Finucane MM, Stevens GA, Cowan MJ, Danaei G, Lin JK, Paciorek CJ, et al. National, regional, and global trends in body-mass index since 1980: systematic analysis of health examination surveys and epidemiological studies with 960 country-years and 9.1 million participants. Lancet. 2011 Feb 12;377(9765):557-67.

2. Overweight and obesity - BMI statistics [Internet].; 2017 []. Available from: http://ec.europa.eu/eurostat/statistics-explained/index.php/Overweight_and_obesity_-_BMI_statistics.

3. WHO. The challenge of Obesity in the WHO European Region and the Strategies for Response: Summary. Copenhagen: World Health Organization, Regional Office for Europe; 2007.

4. Seidell JC. Prevalence and time trends of obesity in Europe. Journal of endocrinological investigation. 2002;25(10):816-22.

5. Eurostat. European Health Interview Survey - Almost 1 adult in 6 in the EU is considered obese - Share of obesity increases with age and decreases with education level. 2016.

6. Field AE, Coakley EH, Must A, Spadano JL, Laird N, Dietz WH, et al. Impact of overweight on the risk of developing common chronic diseases during a 10-year period. Archives of Internal Medicine. 2001 Jul 9;161(13):1581-6.

7. Global BMI Mortality C, Di Angelantonio E, Bhupathiraju S, Wormser D, Gao P, Kaptoge S, et al. Body-mass index and all-cause mortality: individual-participant-data meta-analysis of 239 prospective studies in four continents. Lancet. 2016 Aug 20;388(10046):776-86.

8. Alley DE, Lloyd J, Shardell M. Can obesity account for cross-national differences in life expectancy trends? In: Crimmins EM, Preston SH, Cohen B, editors. International differences in mortality at older ages: Dimensions and sources, panel on understanding divergent trends in longevity in high-income countries. Washington, DC: National Academies Press; 2011. p. 164-92.

9. Olshansky SJ, Passaro DJ, Hershow RC, Layden J, Carnes BA, Brody J, et al. A potential decline in life expectancy in the United States in the 21st century. The New England Journal of Medicine. 2005 Mar 17;352(11):1138-45.

10. Preston SH, Stokes A. Contribution of obesity to international differences in life expectancy. American Journal of Public Health. 2011 Nov;101(11):2137-43.

11. National Research Council. The Role of Obesity. In: Crimmins EM, Preston SH, Cohen B, editors. Explaining Divergent Levels of Longevity in High-Income Countries. Washington, DC: National Academies Press; 2011. p. 43-55.

For peer review only - http://bmjopen.bmj.com/site/about/guidelines.xhtml

12. Leon DA. Trends in European life expectancy: a salutary view. International Journal of Epidemiology. 2011 Apr;40(2):271-7.

13. NCD Risk Factor Collaboration. Trends in adult body-mass index in 200 countries from 1975 to 2014: a pooled analysis of 1698 population-based measurement studies with 19.2 million participants. Lancet. 2016 Apr 2;387(10026):1377-96.

14. Lobstein T, Leach RJ. Workpackage 7: Overweight and Obesity Report on data collection for overweight and obesity prevalence and related relative risks. 2010.

15. Global BMI Mortality Collaboration. Body-mass index and all-cause mortality: individual-participant-data meta-analysis of 239 prospective studies in four continents. Lancet. 2016 Aug 20;388(10046):776-86.

16. University of California, Berkeley (USA), and Max Planck Institute for Demographic Research (Germany). [Internet]. []. Available from: http://www.mortality.org.

17. Cleveland WS, Loader C. Smoothing by local regression: Principles and methods. In: Murray Hill: A&T Bell Laboratorie; 1995.

18. Rockhill B, Newman B, Weinberg C. Use and misuse of population attributable fractions. American Journal of Public Health. 1998 Jan;88(1):15-9.

19. Preston SH, Heuveline P, Guillot M. Demography. Malden, MA: Blackwell; 2001.

20. Census data [Internet].; 2011 []. Available from: https://ec.europa.eu/CensusHub2/query.do?step=selectHyperCube&qhc=false.

21. Flegal KM, Panagiotou OA, Graubard BI. Estimating population attributable fractions to quantify the health burden of obesity. Annals of Epidemiology. 2015 Mar;25(3):201-7.

22. Flegal KM, Graubard BI, Williamson DF, Gail MH. Excess deaths associated with underweight, overweight, and obesity. JAMA. 2005 Apr 20;293(15):1861-7.

23. Mehta NK, Chang VW. Secular declines in the association between obesity and mortality in the United States. Population and Development Review. 2011;37(3):435-51.

24. Yu Y. Reexamining the declining effect of age on mortality differentials associated with excess body mass: evidence of cohort distortions in the United States. American Journal of Public Health. 2012 May;102(5):915-22.

25. Yu Y. The Changing Body Mass-Mortality Association in the United States: Evidence of Sex-Specific Cohort Trends from Three National Health and Nutrition Examination Surveys. Biodemography and Social Biology. 2016;62(2):143-63.

26. Vidra N, Bijlsma MJ, Janssen F. Impact of Different Estimation Methods on Obesity-Attributable Mortality Levels and Trends: The Case of The Netherlands. Int J Environ Res Public Health. 2018 Sep 29;15(10):10.3390/ijerph15102146.

27. Trias-Llimós S, Kunst AE, Jasilionis D, Janssen F. The contribution of alcohol to the East-West life expectancy gap in Europe from 1990 onward. European Journal of Epidemiology. 2017;47(3):731-9.

For peer review only - http://bmjopen.bmj.com/site/about/guidelines.xhtml

28. Doak CM, Wijnhoven TM, Schokker DF, Visscher TL, Seidell JC. Age standardization in mapping adult overweight and obesity trends in the WHO European Region. Obesity Reviews. 2012 Feb;13(2):174-91.

29. Cutler DM, Glaeser EL, Shapiro JM. Why Have Americans Become More Obese? The Journal of Economic Perspectives. 2003;17(1):93-118.

30. Obesity update 2012 [Internet].; 2012 []. Available from: http://www.oecd.org/els/health-systems/49716427.pdf.

31. Silventoinen K, Sans S, Tolonen H, Monterde D, Kuulasmaa K, Kesteloot H, et al. Trends in obesity and energy supply in the WHO MONICA Project. International Journal of Obesity and Related Metabolic Disorders. 2004 May;28(5):710-8.

32. Malik VS, Willett WC, Hu FB. Global obesity: trends, risk factors and policy implications. Nature reviews Endocrinology. 2013 Jan;9(1):13-27.

33. Zatonski WA, Bhala N. Changing trends of diseases in Eastern Europe: closing the gap. Public Health. 2012 Mar;126(3):248-52.

34. Bray GA, Bouchard C. Handbook of Obesity: Etiology and Pathophysiology. second ed. Boca Raton: CRC Press Taylor & Francis Group; 2003.

35. Monteiro CA, Conde WL, Lu B, Popkin BM. Obesity and inequities in health in the developing world. International Journal of Obesity. 2004 Sep;28(9):1181-6.

36. Wilkins E, Wilson L, Wickramasinghe K, Bhatnagar P, Leal J, Luengo-Fernandez R, et al. European Cardiovascular Disease Statistics 2017. Brussels: European Heart Network; 2017.

37. Robertson A, Lobstein T, Knai C. Obesity and socio-economic groups in Europe: Evidence review and implications for action. Brussels: European Commission. 2007.

38. Fagerli RA, Wandel M. Gender differences in opinions and practices with regard to a "healthy diet". Appetite. 1999 Apr;32(2):171-90.

39. Psaltopoulou T, Hatzis G, Papageorgiou N, Androulakis E, Briasoulis A, Tousoulis D. Socioeconomic status and risk factors for cardiovascular disease: Impact of dietary mediators. Hellenic Journal of Cardiology. 2017 Jan - Feb;58(1):32-42.

40. WHO. Obesity and inequities. Guidance for addressing inequities in overweight and obesity. Copenhagen: World Health Organization, Regional Office for Europe; 2014.

Figure legends

Figure 1: Age-standardised obesity-attributable mortality fractions in 26 European countries (by 5 regions) and USA, 1975-2014, 18-100 years

Footnote:

Countries within the same region are presented with the same colour

For peer review only - http://bmjopen.bmj.com/site/about/guidelines.xhtml

Central Europe: Czech Republic, Hungary, Poland, Slovakia

Eastern Europe: Belarus, Estonia, Ukraine, Latvia, Lithuania, Russian Federation

Northern Europe: Denmark, Finland, Iceland, Norway, Sweden

Southern Europe: Italy, Portugal, Spain

Western Europe: Austria, Belgium, France, Ireland, Luxembourg, Netherlands, Switzerland, United Kingdom

USA: United States of America

Figure 2: Potential gains in life expectancy at birth (PGLE) if obesity-attributable mortality was eliminated, in 26 European countries (differentiating Western and Central Eastern Europe) and the USA, 1975-2012, 18-100 years

Footnote:

Countries within the same region are presented with the same colour

Central Eastern Europe: Belarus, Czech Republic, Estonia, Hungary, Latvia, Lithuania, Poland, Russian Federation, Slovakia, Ukraine

Western Europe: Austria, Belgium, Denmark, Finland, France, Iceland, Ireland, Italy, Luxembourg, Netherlands, Norway, Portugal, Spain, Sweden, Switzerland, United Kingdom

USA: United States of America

Figure 3: Average annual increase (%) in potential gains in life expectancy due to obesity in 26 European countries and the USA between 1975-2012, in men

Figure 4: Average annual increase (%) in potential gains in life expectancy due to obesity in 26 European countries and the USA between 1975-2012, in women

For peer review only

For peer review only - http://bmjopen.bmj.com/site/about/guidelines.xhtml

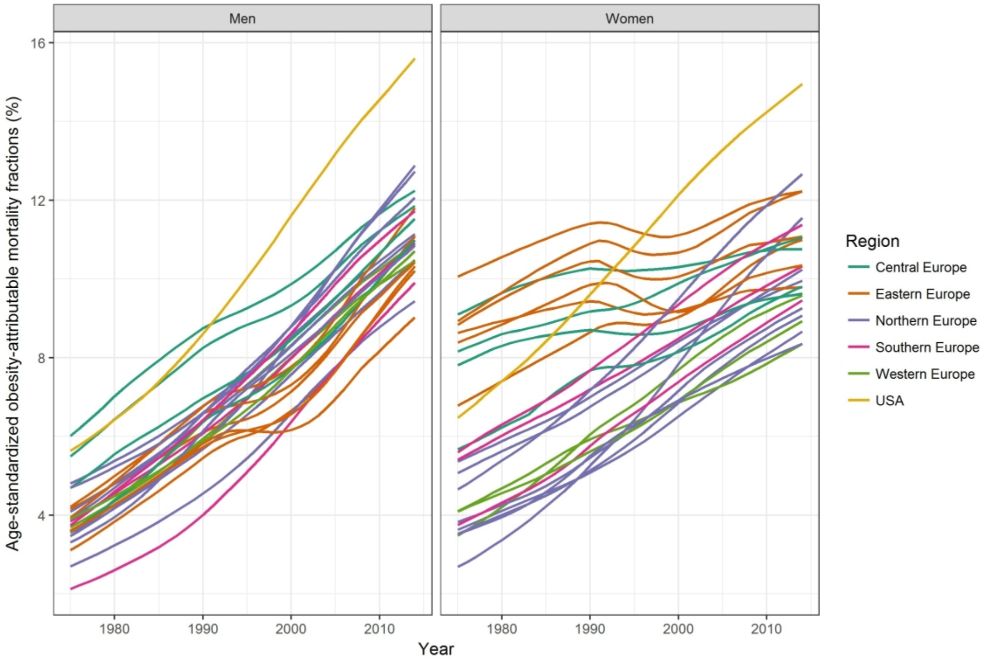

Figure 1: Age-standardised obesity-attributable mortality fractions in 26 European countries (by 5 regions) and USA, 1975-2014, 18-100 years.Footnote: Countries within the same region are presented with the same colour
Central Europe: Czech Republic, Hungary, Poland, SlovakiaEastern Europe: Belarus, Estonia, Ukraine, Latvia, Lithuania, Russian FederationNorthern Europe: Denmark, Finland, Iceland, Norway, SwedenSouthern Europe: Italy, Portugal, SpainWestern Europe: Austria, Belgium, France, Ireland, Luxembourg, Netherlands, Switzerland, United Kingdom
USA: United States of America

228x152mm (300 x 300 DPI)

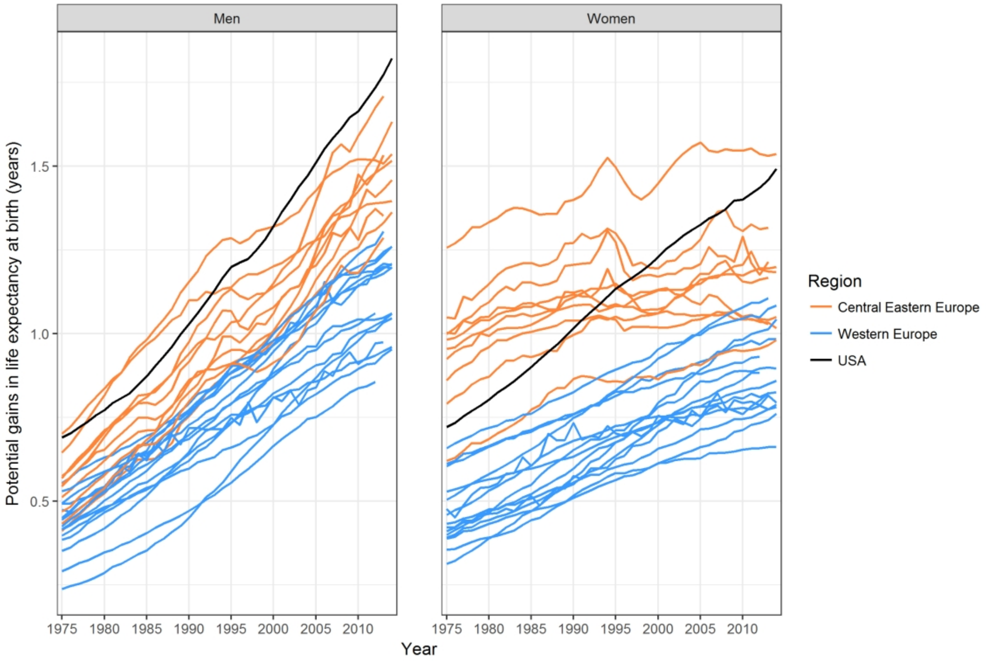

Figure 2: Potential gains in life expectancy at birth (PGLE) if obesity-attributable mortality was eliminated, in 26 European countries (differentiating Western and Central Eastern Europe) and the USA, 1975-2012, 18-100 years
Footnote: Countries within the same region are presented with the same colourCentral Eastern Europe: Belarus, Czech Republic, Estonia, Hungary, Latvia, Lithuania, Poland, Russian Federation, Slovakia, Ukraine
Western Europe: Austria, Belgium, Denmark, Finland, France, Iceland, Ireland, Italy, Luxembourg, Netherlands, Norway, Portugal, Spain, Sweden, Switzerland, United KingdomUSA: United States of America

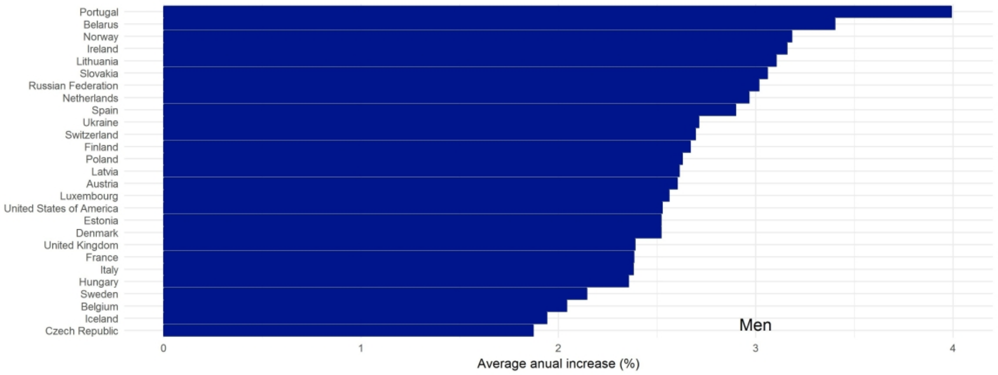

Figure 3: Average annual increase (%) in potential gains in life expectancy due to obesity in 26 European countries and the USA between 1975-2012, in men

1244x472mm (72 x 72 DPI)

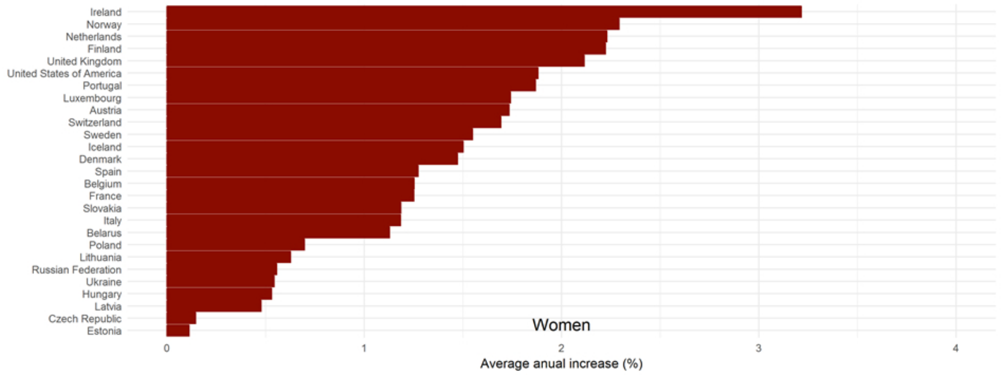

Figure 4: Average annual increase (%) in potential gains in life expectancy due to obesity in 26 European countries and the USA between 1975-2012, in women

Supplementary material

Table S1: Age-and sex-specific RRs of dying from obesity from the Dynamo project (Lobstein et al. 2010)

**Age**	**RR**	
	**Men**	**Women**
**<50**	1.55	1.5
**50-59**	1.539	1.49
**60-69**	1.5225	1.475
**70+**	1.495	1.45

Reference group for the RRs: normal weight (18-24.9 kg/m^2)

For peer review only - http://bmjopen.bmj.com/site/about/guidelines.xhtml

Figure S1: Age-standardised obesity-attributable mortality fractions in 26 European countries, grouped by 5 regions and USA, 1975-2014, 18-100 years

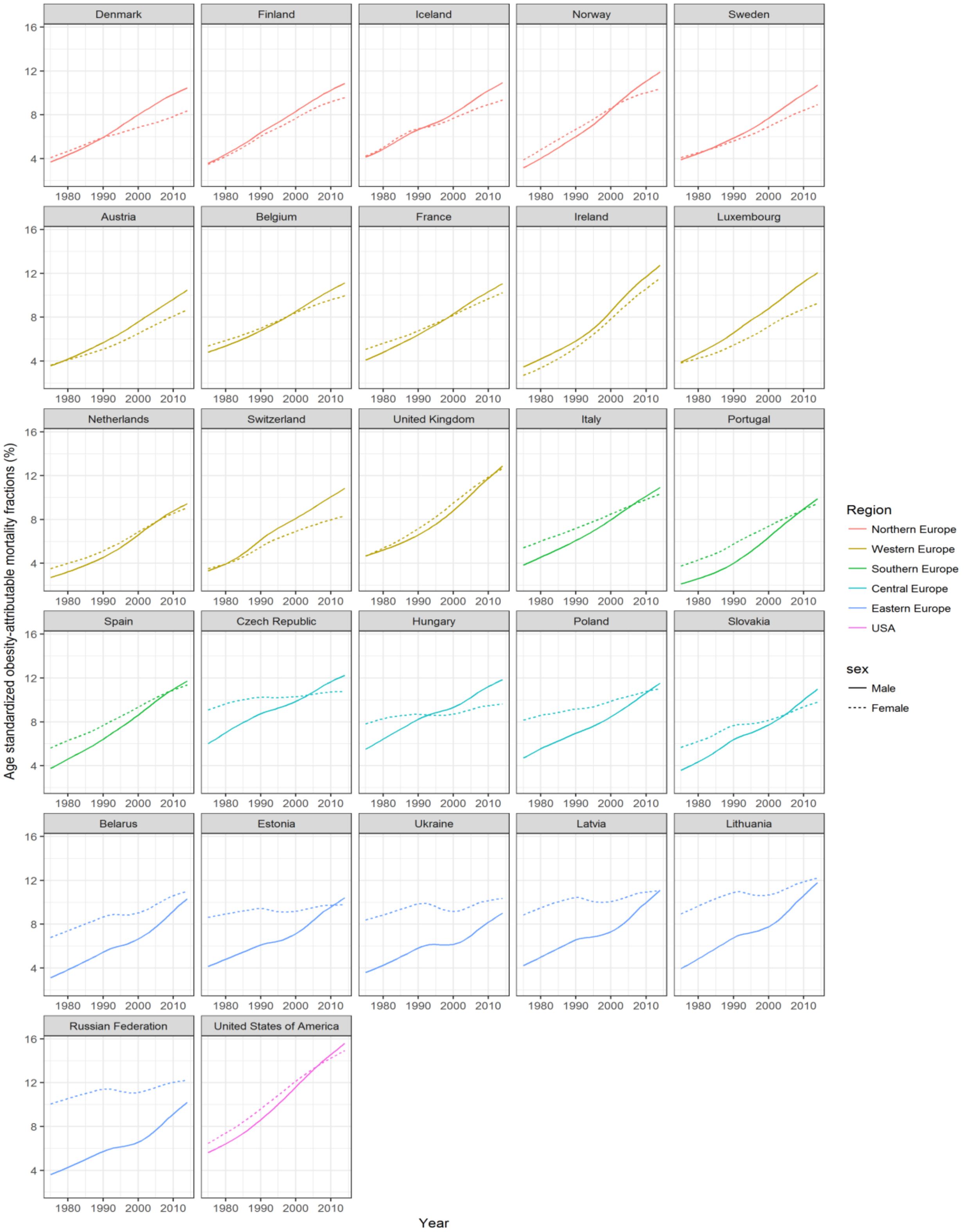

Countries within the same region are presented with the same colour

Figure S2: Potential gains in life expectancy at birth (PGLE) if obesity-attributable mortality was eliminated, in 26 European countries, grouped by 2 regions and USA, 1975-2012, 18-100 years

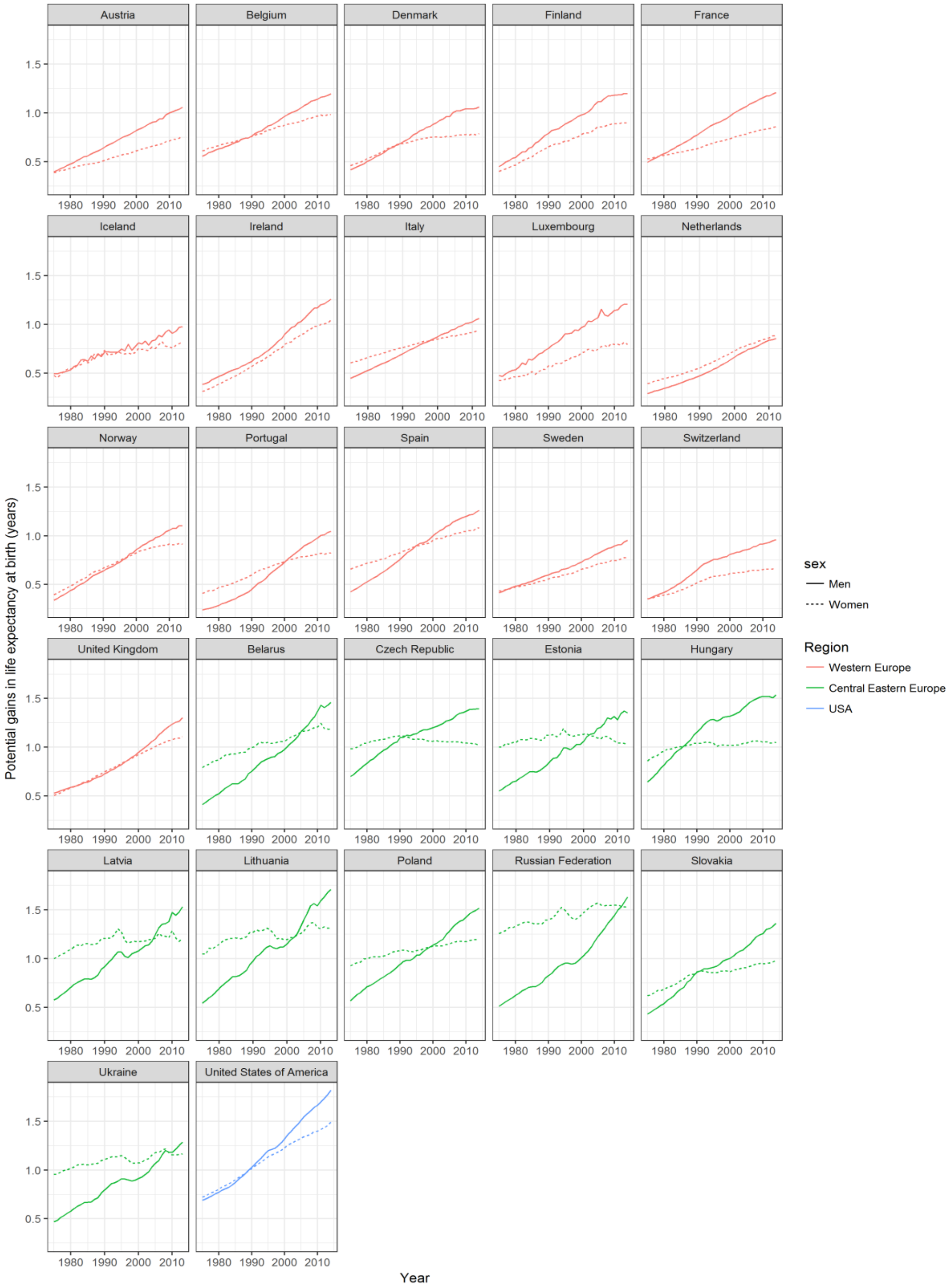

Countries within the same region are presented with the same colour

Table S2: Potential gains in life expectancy at birth (PGLE) if obesity-attributable mortality was eliminated, in 26 European countries (differentiating Western and Central Eastern Europe) and the USA, in 1975 and 2012, 18-100 years

	PGLE 1975		PGLE 2012	
**Country**	***Men***	***Women***	***Men***	***Women***
Central Eastern Europe (CEE)				
Belarus	0.41	0.79	1.41	1.19
Czech Republic	0.70	0.98	1.39	1.03
Estonia	0.55	1.00	1.37	1.04
Hungary	0.64	0.86	1.52	1.04
Latvia	0.58	1.00	1.48	1.18
Lithuania	0.54	1.05	1.67	1.31
Poland	0.57	0.93	1.48	1.19
Russian Federation	0.51	1.26	1.53	1.54
Slovakia	0.43	0.62	1.31	0.96
Ukraine	0.47	0.95	1.25	1.16
**Average CEE**	0.54	0.94	1.44	1.16
Western Europe				
Austria	0.40	0.39	1.03	0.73
Belgium	0.55	0.61	1.17	0.97
Denmark	0.42	0.46	1.04	0.79
France	0.49	0.53	1.18	0.84
Finland	0.45	0.40	1.19	0.90
Ireland	0.38	0.31	1.21	1.01
Iceland	0.49	0.48	0.97	0.80
Italy	0.44	0.60	1.06	0.93
Luxembourg	0.47	0.42	1.19	0.79
Netherlands	0.29	0.39	0.86	0.88
Norway	0.34	0.39	1.07	0.91
Portugal	0.24	0.41	1.01	0.81
Spain	0.42	0.66	1.22	1.05
Sweden	0.42	0.43	0.91	0.76
Switzerland	0.35	0.35	0.93	0.66
United Kingdom	0.53	0.50	1.27	1.09
**Average Western Europe**	0.41	0.48	1.08	0.86
USA	0.69	0.72	1.73	1.44
**Average European countries**	0.46	0.64	1.22	0.98
**Average all countries**	0.47	0.64	1.23	1.00

For peer review only - http://bmjopen.bmj.com/site/about/guidelines.xhtml

Table S3: Potential gains in life expectancy at age 50 (PGLE) if obesity-attributable mortality was eliminated, own and Preston estimates, in the same countries studied, in 2006

	PGLE e50 2006, own estimates	PGLE e50 2006, Preston's estimates	Difference
**Country**			
Men			
Austria	0.81	1.00	-0.19
Belgium	0.95	0.98	-0.03
Czech Republic	1.14	1.34	-0.20
Denmark	0.88	0.82	0.06
France	0.94	0.99	-0.05
Italy	0.88	0.90	-0.02
Netherlands	0.69	0.73	-0.04
Poland	1.14	1.37	-0.23
Spain	1.02	1.15	-0.13
Sweden	0.75	0.72	0.03
Switzerland	0.77	0.79	-0.02
United Kingdom	0.99	1.34	-0.35
USA	1.29	1.85	-0.56
Women			
Austria	0.62	0.71	-0.09
Belgium	0.86	0.73	0.13
Czech Republic	0.87	1.01	-0.14
Denmark	0.71	0.62	0.09
France	0.72	0.52	0.2
Italy	0.84	0.57	0.27
Netherlands	0.76	0.69	0.07
Poland	1.08	1.19	-0.11
Spain	0.95	0.87	0.08
Sweden	0.67	0.63	0.04
Switzerland	0.59	0.50	0.09
United Kingdom	0.94	1.23	-0.29
USA	1.18	1.28	-0.10

For peer review only - http://bmjopen.bmj.com/site/about/guidelines.xhtml

Figure S3: Age-standardised obesity prevalence in 26 European countries, grouped by 2 regions and USA, 1975-2012, 18-100 years

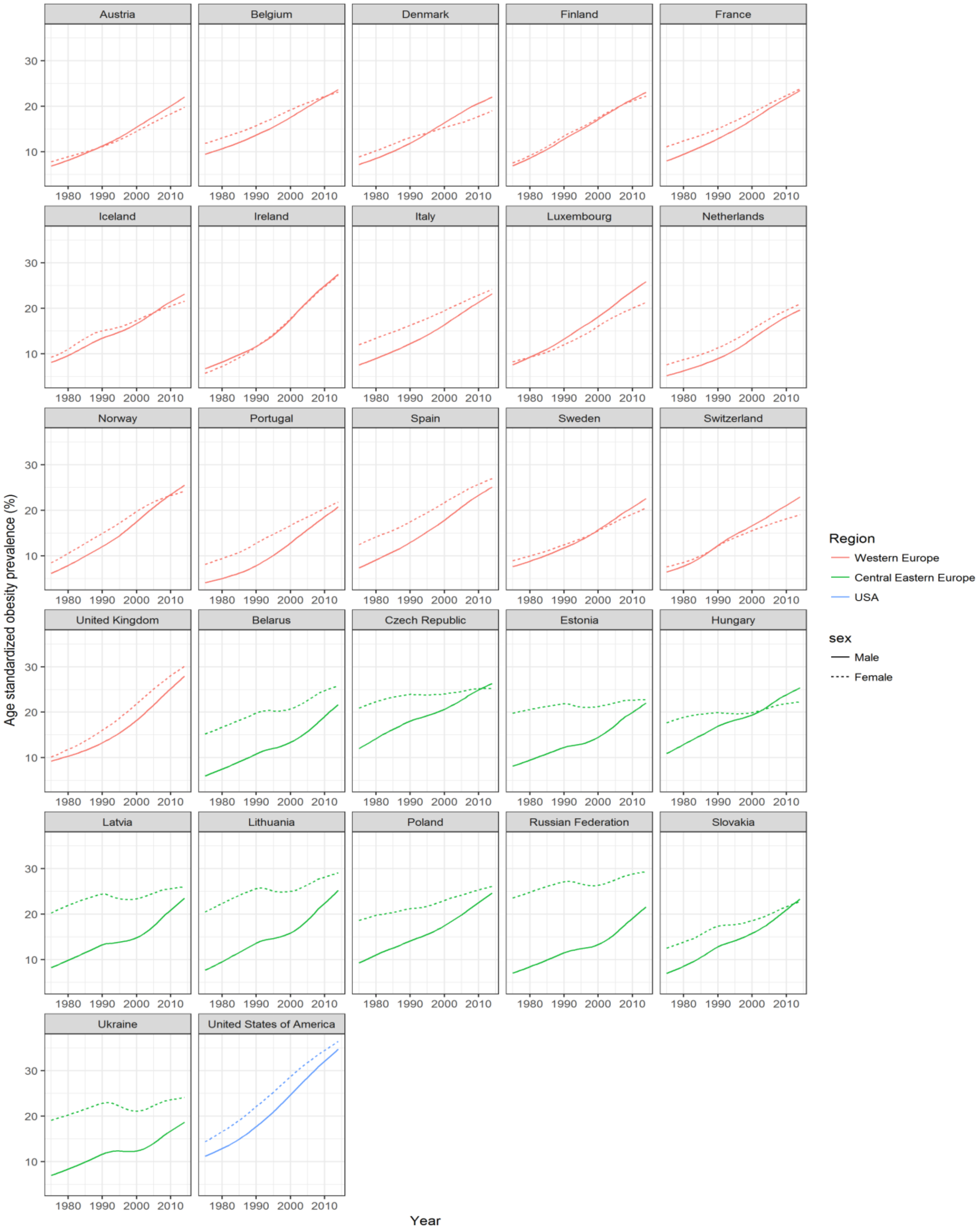

Countries within the same region are presented with the same colour

BMJ Open

Impact of obesity on life expectancy among different European countries, 1975-2012

Journal:	*BMJ Open*
Manuscript ID	bmjopen-2018-028086.R1
Article Type:	Research
Date Submitted by the Author:	28-Apr-2019
Complete List of Authors:	Vidra, Nikoletta; Population Research Centre, Faculty of Spatial Sciences, Demography<br>Trias-Llimós, Sergi; University of Groningen, Population Research Centre<br>Janssen, Fanny; University of Groningen, Population Research Centre; Nederlands Interdisciplinair Demografisch Instituut
**Primary Subject Heading**:	Epidemiology
Secondary Subject Heading:	Public health, Nutrition and metabolism
Keywords:	EPIDEMIOLOGY, NUTRITION & DIETETICS, PUBLIC HEALTH

SCHOLARONE™
Manuscripts

Impact of obesity on life expectancy among different European countries, 1975-2012

Nikoletta Vidra[1], Sergi Trias-Llimós[1], Fanny Janssen[1,2]

[1] Population Research Centre, Faculty of Spatial Sciences, University of Groningen, Landleven 1, 9747 AD Groningen, The Netherlands.

[2] Netherlands Interdisciplinary Demographic Institute, Lange Houtstraat 19, 2511 CV The Hague, The Netherlands.

Corresponding author: Nikoletta Vidra, Population Research Centre, Faculty of Spatial Sciences, University of Groningen, PO Box 800, 9700 AV Groningen, The Netherlands. Tel: 0031 (0) 50 363 86 52; e-mail: n.vidra@rug.nl

Word count: 3669 words

For peer review only

Abstract

Objective: This study assesses the impact of obesity on life expectancy for 26 European national populations and the USA over the 1975-2012 period.

Design: Secondary analysis of population-level obesity and mortality data.

Setting: European countries, namely Austria, Belarus, Belgium, the Czech Republic, Denmark, Estonia, Finland, France, Hungary, Iceland, Ireland, Italy, Latvia, Lithuania, Luxembourg, the Netherlands, Norway, Poland, Portugal, the Russian Federation, Slovakia, Spain, Sweden, Switzerland, Ukraine, the United Kingdom (UK); and the USA.

Participants: National populations aged 18–100 years, by sex.

Measurements: Using data by age and sex, we calculated obesity-attributable mortality by multiplying all-cause mortality (Human Mortality Database) with obesity-attributable mortality fractions (OAMFs). OAMFs were obtained by applying the weighted-sum method to obesity prevalence data (NCD Risk Factor Collaboration) and European Relative Risks (RRs) (DYNAMO). We estimated potential gains in life expectancy at birth (PGLE) by eliminating obesity-attributable mortality from all-cause mortality using associated single-decrement life tables.

Results: In the 26 European countries in 2012, PGLE due to obesity ranged from 0.86 to 1.67 years among men, and from 0.66 to 1.54 years among women. In all countries, PGLE increased over time, with an average annual increase of 2.68% among men and 1.33% and among women. Among women in Denmark, Switzerland, and Central and Eastern European countries, the increase in PGLE levelled off after 1995. Without obesity, the average increase

For peer review only

in life expectancy between 1975 and 2012 would have been 0.78 years higher among men and 0.30 years higher among women.

Conclusions: Obesity was proven to have an impact on both life expectancy levels and trends in Europe. The differences found in this impact between countries and the sexes can be linked to contextual factors, as well as to differences in people's ability and capacity to adopt healthier lifestyles.

Keywords: Obesity, life expectancy, Europe, USA

Article Summary

Strengths and limitations of the study

- This is the first study to assess the impact of obesity on life expectancy at birth over time, and we do so here for 26 European countries and the USA.
- We used recent long-term comparable data on obesity prevalence based on population-based measurement studies, and European relative risks of dying from obesity by age and sex from a recent meta-analysis.
- Because of remaining data limitations regarding prevalence and relative risks, we had to use a fairly simple – albeit common applied - methodology to estimate obesity-attributable mortality.

For peer review only

Introduction

Obesity is a global epidemic (1), with Europe currently ranking second worldwide after the USA (2). Over the last 20 years obesity prevalence has increased threefold in Europe(3), although not uniformly across countries (4). Estimates for 2014 indicate that obesity varied threefold across European countries, ranging from a low of 9% in Romania to a high of 26% in Malta (5). Obesity constitutes a serious health burden at the individual and population levels because it is associated with an increased risk of morbidity (6), and mortality (7). However, the potential impact of the increase in obesity on life expectancy trends remains largely unknown (8).

The few existing studies that assessed the impact of obesity on life expectancy at the population level provided estimates at one specific point in time only (9, 10). Olshansky et al. found that if obesity was eliminated, life expectancy at birth (e0) in the USA in 2000 would be 0.21 to 1.08 years higher, depending on gender and ethnicity (9). Preston et al. (10) estimated for 16 low-mortality countries in 2006 that the reduction in life expectancy at age 50 (e50) due to obesity was greatest in the USA, at more than 1.5 years; and ranged from 0.50 to 1.19 years for women and from 0.72 to 1.37 years for men in European countries.

Gaining insight into the impact of obesity on trends in life expectancy is especially relevant (11) given the marked differences in life expectancy trends across Europe (12). In Western European countries, e0 has been increasing steadily, and has risen six to eight years since 1970. But in Central and Eastern Europe (CEE), e0 stagnated or even declined between the 1970s and the 1980s, and did not start increasing again until the 1990s. There are also marked differences in e0 trends between individual European countries (12).

For peer review only - http://bmjopen.bmj.com/site/about/guidelines.xhtml

In light of these important differences between European countries in both obesity prevalence and life expectancy over time, our aim is to assess the impact of obesity on long-term trends in life expectancy across a wide range of European countries.

Our sole focus is on the impact of obesity, given the significant health burden caused by obesity, the large body of literature on its impact, and the well-documented association of obesity with mortality.

Data and Methods

Setting

We studied the impact of obesity on life expectancy by sex over the 1975-2012 period in 26 European countries: Austria, Belarus, Belgium, the Czech Republic, Denmark, Estonia, Finland, France, Hungary, Iceland, Ireland, Italy, Latvia, Lithuania, Luxembourg, the Netherlands, Norway, Poland, Portugal, the Russian Federation, Slovakia, Spain, Sweden, Switzerland, Ukraine, the United Kingdom (UK); and the USA as a comparison country (10).

Data

Long-term comparable obesity prevalence data (BMI≥30kg/m2) by country, sex, age (18-19, 20-24, …, 85+), and year (1975-2012), based on 1698 population-based measurement studies, were obtained from the NCD Risk Factor Collaboration study (13). These validated data comprise the available measured height and weight data of 19.2 million participants from representative data sources, supplemented with estimates based on information from other years and related countries from a Bayesian hierarchical model (13). The same model was applied to all countries and used as an input the measured weight and height data, including covariates that help predict BMI (13).

For peer review only - http://bmjopen.bmj.com/site/about/guidelines.xhtml

For peer review only

The age- (<50, 50-59, 60-69 and ≥70 years) and sex-specific relative risks (RRs) of dying from obesity (see Supplementary Material Table S1) came from a review of studies mainly conducted in Western Europe and the USA, with the normal-weight category used as the reference group (18.5≥BMI≤25 kg/m^2) (14). These age- and sex-specific RRs were largely in line with the overall European RR of 1.64 recently estimated by the Global BMI Mortality Collaboration (7). The differences across age groups found in that study were similar with those reported in our findings (i.e., higher RRs at younger than at older ages), though they were less distinct (7). In addition, the use of RRs with the normal weight category as the reference category is in line with previous studies that estimated obesity-attributable mortality (15-19), while the estimation of obesity-attributable mortality with such a RR can be considered the theoretically maximally possible attributable mortality (20).

All-cause mortality numbers and exposure population data by single year of age, sex, year, and country were obtained from the Human Mortality Database (21). These data are of high quality and are widely used within the demographic community and beyond (22).

For peer review only

Patient and Public Involvement

No patients were involved in this study.

Methods

We performed our analyses separately by country and sex, based on data by single year of age (18-100). The obesity prevalence data were turned into single-age prevalence (18-100) by applying Loess smoothing (23). The RRs were turned into single-year RRs (18- 100) using linear regression.

For peer review only - http://bmjopen.bmj.com/site/about/guidelines.xhtml

To estimate the obesity-attributable mortality fraction (OAMF) – i.e., the share of all-cause mortality due to obesity – we used the Rockhill formula to estimate OAMFs by age (a) and sex (s) (24).

$$OAMF_{a,s} = \frac{P_{a,s} \cdot (RR_{a,s} - 1)}{1 + (P_{a,s} \cdot (RR_{a,s} - 1))} \text{(Equation 1)}$$

where *P* is the obesity prevalence. We then weighted the $OAMF_{a,s}$ with the corresponding number of deaths.

For the estimation of the impact of obesity on life expectancy (see 2.3.2) we needed age-and sex-specific (non-) obesity-attributable mortality rates. These were obtained by multiplying $OAMF_{a,s}$ and [1- $OAMF_{a,s}$], respectively, with age- and sex-specific all-cause mortality rates.

To ensure comparability across countries, over time, and between men and women, we applied direct age- and sex-standardisation (25) to obesity prevalence, obesity-attributable mortality fractions, and obesity-attributable mortality rates, using the European population of 2011 (26) as the standard.

To assess the impact of adult obesity on e_0, we calculated for each country the potential gain in life expectancy (PGLE) if obesity-attributable mortality were eliminated, by calendar year and sex and is in line with the approach by Preston et al. First, we calculated e_0 by applying standard life table techniques to age-specific all-cause mortality rates (0-100)(25)). Second, we applied associated single-decrement life tables (ASDLT)(25)) to age- and sex-specific non-obesity-attributable mortality rates (0-100) to obtain e_0 if obesity-attributable mortality were eliminated. The PGLE represents the difference between the e_0 based on the ASDLT and the original e_0.

For peer review only

For peer review only - http://bmjopen.bmj.com/site/about/guidelines.xhtml

To summarise the changes in PGLE across countries, we estimated the average annual changes in PGLE (in %):

$$Average\ annual\ changes\ in\ PGLE\ (\%) = \frac{\sum_{t=1976}^{2012} \frac{(PGLE_t - PGLE_{t-1})}{PGLE_{t-1}}}{2012 - 1975} 100$$

To assess the impact of obesity on time trends in e_0 between 1975 and 2012, we subtracted the observed change in e_0 from the change in e_0 without obesity. The change in e_0 without obesity was obtained by using the e_0 values from the associated single-decrement life tables applied to non-obesity-attributable mortality for 1975 and 2012.

Results

For the 26 European countries, the age-standardised obesity-attributable mortality fraction (OAMF) was, on average, 11% among men and 10% among women in 2012. For the USA, these estimates were substantially higher; i.e., 15% and 14%, respectively. The average OAMF levels were higher in Northern, Western, and Southern Europe combined (hereafter, Western Europe) than in CEE among men, while the opposite was the case among women.

OAMFs were increasing over time for all countries and both sexes, although not to the same extent (see Figure 1, Figure S1). In Western Europe, OAMFs generally increased over the 1975-2012 period, and at a faster pace among men. In CEE, by contrast, OAMFs clearly stagnated, and even declined between 1990 and 2000. The overall increase in OAMFs was greatest in the USA, Ireland, Norway (men), and the UK (women).

For peer review only

Figure 1: Age-standardised obesity-attributable mortality fractions in 26 European countries* (by 5 regions) and USA, 1975-2014, 18-100 years

* Countries within the same region are presented with the same colour

Central Europe: Czech Republic, Hungary, Poland, Slovakia

Eastern Europe: Belarus, Estonia, Ukraine, Latvia, Lithuania, Russian Federation

Northern Europe: Denmark, Finland, Iceland, Norway, Sweden

Southern Europe: Italy, Portugal, Spain

Western Europe: Austria, Belgium, France, Ireland, Luxembourg, Netherlands, Switzerland, United Kingdom

USA: United States of America

<approximately here>

In the 26 European countries in 2012, estimates of potential gains in life expectancy at birth (PGLE) if obesity was eliminated ranged from 0.86 to 1.67 years among men (1.22 on average) and from 0.66 to 1.54 years (0.98 on average) among women (Figure 2; Figure S2 and Table S2 Supplementary Material). Among men in the USA, the PGLE estimate was, at 1.73 years, slightly higher than the highest estimate in Europe; and among women in the USA, the PGLE estimate was, at 1.44 years, the second-highest after the estimate for Russia. The average PGLE estimate was 1.08 among men and 0.86 among women in Western Europe, and was 1.44 among men and 1.16 among women in CEE (see Supplementary Material, Table S2).

For peer review only

Figure 2: Potential gains in life expectancy at birth (PGLE) if obesity-attributable mortality was eliminated, in 26 European countries* (differentiating Western and Central Eastern Europe) and the USA, 1975-2012

* Countries within the same region are presented with the same colour

Central Eastern Europe: Belarus, Czech Republic, Estonia, Hungary, Latvia, Lithuania, Poland, Russian Federation, Slovakia, Ukraine

Western Europe: Austria, Belgium, Denmark, Finland, France, Iceland, Ireland, Italy, Luxembourg, Netherlands, Norway, Portugal, Spain, Sweden, Switzerland, United Kingdom

USA: United States of America

<approximately here>

Overall, from 1975 to 2012, PGLE due to obesity increased in all of the countries (Figure 2, Figure S2, Figure 3, 4). The increase was greater among men (average annual increase of 2.68%) than among women (average annual increase of 1.33%), was largest among men in Portugal and Belarus and among women in Portugal, and was substantial among men and women in Norway (Figures 3, 4). While there was a general increase in PGLE due to obesity, this trend stagnated among women in CEE from around 1990 onwards, and levelled off after 1995 among women in Denmark and Switzerland.

Figure 3: Average annual increase (%) in potential gains in life expectancy due to obesity in 26 European countries and the USA between 1975-2012, in men

<approximately here>

Figure 4: Average annual increase (%) in potential gains in life expectancy due to obesity in 26 European countries and the USA between 1975-2012, in women

<approximately here>

For peer review only - http://bmjopen.bmj.com/site/about/guidelines.xhtml

For peer review only

Table 1 shows the impact of obesity on time trends in life expectancy at birth (e_0). Overall, the average increase in e_0 between 1975 and 2012 was 7.26 years for men and 6.28 years for women in the 26 European countries. Without obesity, the average increase in e_0 would have been 8.04 years for men and 6.58 years for women; or 0.78 and 0.30 years higher, respectively. Among men, obesity had the greatest impact on e_0 trends in Lithuania and the USA (more than one year), and the smallest impact in Iceland and Sweden (0.5 years). Among women, obesity had the greatest impact on e_0 trends in the USA and Ireland (0.7 years) and the smallest impact in Estonia and the Czech Republic (less than 0.1 year).

Table 1: Impact of obesity on trends in life expectancy at birth (e_0) in 26 European countries and USA 1975-2012, by sex

Country	Change in e_0 with obesity 2012-1975 (years)		Change in e_0 without obesity 2012-1975 (years)		Effect of obesity on e_0 change 2012-1975 (years)	
	Men	*Women*	*Men*	*Women*	*Men*	*Women*
Austria	10.62	8.61	11.25	8.95	0.63	0.34
Belarus	-0.55	1.43	0.46	1.83	1.00	0.40
Belgium	8.85	7.63	9.46	7.99	0.61	0.36
Czech Republic	7.97	6.98	8.66	7.03	0.69	0.05
Denmark	6.78	5.03	7.40	5.36	0.63	0.33
Estonia	6.43	6.42	7.26	6.46	0.82	0.04
France	9.49	7.99	10.17	8.30	0.68	0.31
Finland	10.07	7.26	10.82	7.75	0.74	0.50
Hungary	5.29	6.18	6.17	6.36	0.87	0.18
Iceland	9.02	5.19	9.51	5.51	0.48	0.32
Ireland	9.40	8.40	10.22	9.10	0.83	0.69
Italy	10.19	8.56	10.81	8.89	0.62	0.33
Latvia	4.91	4.53	5.82	4.70	0.90	0.18
Lithuania	2.01	3.80	3.14	4.06	1.13	0.26
Luxembourg	11.78	9.27	12.50	9.65	0.72	0.37
Netherlands	7.68	5.10	8.26	5.6	0.56	0.49
Norway	7.70	5.33	8.42	5.86	0.74	0.51
Poland	5.90	6.74	6.81	7.00	0.91	0.27
Portugal	12.14	10.87	12.91	11.26	0.77	0.40

For peer review only

For peer review only - http://bmjopen.bmj.com/site/about/guidelines.xhtml

Russian Federation	2.05	2.62	3.06	2.89	1.02	0.28
Slovakia	5.65	5.82	6.52	6.16	0.88	0.34
Spain	8.82	8.75	9.62	9.14	0.79	0.39
Sweden	7.69	5.59	8.18	5.93	0.49	0.33
Switzerland	8.98	6.63	9.55	6.93	0.58	0.30
Ukraine	0.48	1.73	1.26	1.94	0.78	0.21
United Kingdom	9.46	6.96	10.20	7.55	0.74	0.59
USA	7.86	4.89	8.90	5.61	1.04	0.71
**Average CEE countries**	4.01	4.63	4.92	4.84	0.90	0.22
**Average Western countries**	9.10	7.09	9.76	7.50	0.66	0.41

For peer review only

Discussion

Summary of results

In the 26 European countries studied, the share of mortality due to obesity in 2012 was, on average, 11% among men and 10% among women. PGLE due to obesity in 2012 ranged from 0.86 to 1.73 years among men, and from 0.66 to 1.54 years among women. Overall, PGLE increased between 1975 and 2012, albeit more quickly among men (average annual increase: 2.68%) than among women (1.33%). Among women in Denmark, Switzerland, and the CEE countries the increase in PGLE levelled off after 1995. Without obesity, the average increase in e0 between 1975 and 2012 would have been 0.78 years higher among men and 0.30 years higher among women.

Evaluation of data and methods

Using the recent advances in obesity data, it is now possible to study the impact of obesity on life expectancy for a large number of countries and a long period of time. Two methodological issues warrant our attention, however.

First, in calculating the share of mortality due to obesity (OAMF), which also forms the basis for our PGLE calculations, we were hindered by limitations in the available prevalence and RRs data, which also affected the method used. As has previously been documented, OAMF estimates are sensitive to the data and the methods used (27).

In selecting obesity prevalence data, we used the longest validated time series based on population-based measurement studies that are suitable for studying the impact of obesity on long-term life expectancy trends across Europe (13). For those countries with less available obesity data – especially the CEE countries a portion of the data we used were merely the result of modelling. Thus, the resulting estimates should be treated with some caution . By contrast, for the non-CEE countries, most of the data we used pertain to measured data (13). Supplementary Material, Table S3 gives the confidence intervals around the age-standardised prevalence estimates for each country by sex in order, as to provide more information on the relative reliability of the data for the different countries in our analysis.

Because age- and sex-specific RRs of mortality associated with obesity are not readily available by country and year, we have decided to apply to all of the countries studied age- and sex-specific RRs from Western European and US populations that are largely suitable for our setting, as had previously been done (10). Although RRs could differ slightly across contexts, studies that compared RRs across continents found only small differences in RRs between Europe and North America (7). Consequently, we do not expect to observe large differences between individual countries. In addition, as time-variant European RRs were not available, we had to apply time-constant RRs, even though it is possible that changes in the association of obesity with mortality – which could, for example, occur because of improvements in the treatment of chronic diseases – have affected the impact of obesity on

For peer review only - http://bmjopen.bmj.com/site/about/guidelines.xhtml

life expectancy. Previous studies that assessed changes over time in the association of obesity with mortality did so only for the US, and, unfortunately, provided mixed evidence, with some of these studies reporting a decline (18, 28, 29), and others finding an increase (30).Therefore, before implementing time-variant European RRs, more information on their direction is required. Similarly, comparable country-specific RRs are urgently needed. In addition, the choice of these RRs along with their reference group might exert an effect in our estimates. Based on the available data, only a fairly simple – albeit common applied – the weighted sum method could be applied (27) to estimate the OAMFs. The application of a more advanced methodology (27) could have affected the OAMFs and thus the PGLE levels, but less the trends(31). The lack of information on the uncertainty of the RRs we used limited us in estimating confidence intervals for the OAMFs and PGLEs.

Second, besides being the result of the OAMFs, the PGLE estimates can also be affected by all-cause mortality levels and trends as age- and sex-specific all-cause mortality rates are used to estimate PGLE. Since all-cause mortality fluctuated greatly in CEE in the analysed period (12), short-term variations in PGLE in CEE countries should be treated with more caution.

Explanation of results

In 2012, the PGLE due to obesity were, on average, 1.22 years for men and 0.98 years for women in the 26 European countries, and 1.73 years for men and 1.43 years for women in the USA. A comparison of our 2006 e50 estimates with those of Preston et al. (10) for the same countries uncovered only small differences, except among men in the USA (our estimate was 0.56 years lower) and women in the UK (our estimate was 0.29 years lower) (see Supplementary Material, Table S4). Given that approximately the same methodology was used to estimate the OAMFs, the observed differences are most likely due to the use of

For peer review only - http://bmjopen.bmj.com/site/about/guidelines.xhtml

different obesity prevalence and RRs data. Preston used prevalence data from national representative surveys and RRs from the Prospective Studies collaboration (10). Given that the observed differences do not have the same direction for the different countries, we believe that these differences are mainly attributable to the prevalence data used. To further evaluate our observed PGLE levels, we compared them with own PGLE estimates for smoking and alcohol (32). Our PGLE estimates for smoking were 2.38 years for men and 1.00 year for women in Western Europe, and 3.82 years for men and 0.67 years for women in CEE. Our PGLE estimates for alcohol were 0.90 years for men and 0.44 years for women in Western Europe, and 2.15 years for men and 1.00 year for women in CEE (32) while our average PGLE for obesity was 1.08 among men and 0.86 among women in Western Europe, and 1.44 among men and 1.16 among women in CEE. Thus, obesity's impact on life expectancy lies between that of smoking and alcohol, and can be considered significant.

In our study, we found that PGLE due to obesity was increasing, but that this trend differed across countries and between the sexes. This overall trend can be explained by the general increase in obesity prevalence in European countries (see Supplementary Material, Figure S3) (13) and the resulting growth in the burden of obesity (3) , which is also reflected in the OAMFs (Figure 1, Figure S1) in these countries.

At the same time, parts of the observed variation in the increase in PGLE estimates across the USA, Western Europe, and CEE and between the sexes reflect differences in the onset, the development, and the impact of the obesity epidemic in these countries and in men and women. Across the countries studied, the absolute increase in PGLE was largest among women and second-largest among men in the USA. This pattern is in line with evidence showing that between 1980 and 2008, obesity increased much more in the USA

For peer review only - http://bmjopen.bmj.com/site/about/guidelines.xhtml

than in Europe(1, 33). This rapid progression of the obesity epidemic in the USA and its large impact on life expectancy has been attributed to an increasingly obesogenic environment caused by factors such as changes in food preparation and processes that promote the consumption of calorically dense foods, and a pronounced decrease in physical activity levels (34). The obesity epidemic has progressed more slowly in Western Europe than in the USA(1, 13). However, obesity levels in countries like the UK and Ireland are rapidly approaching those in the USA (35), as our PGLE estimates also show.

In the CEE countries, the PGLE trends track the evolution of the obesity epidemic in that region (see Supplementary Material, Figure S3). Obesity levels have been higher in CEE than in Western Europe since 1980 (36, 37), which suggests that the epidemic started earlier in CEE. As a result of this earlier onset, the impact of obesity (as expressed in terms of OAMF and PGLE) in the 1970s and 1980s was at times even greater in CEE than in the USA, especially among women. While there are many potential explanations for this early onset of the obesity epidemic in CEE, the available data indicate that the main factors were the relatively high total energy supply and energy intake in CEE in those years (38).

The overall progress of the obesity epidemic was lower in CEE than in Western Europe, and the increase was not constant (1). Indeed, in CEE, increases in obesity prevalence(1, 39), OAMFs, and PGLE stagnated in the 1980-2008 period, and were more pronounced in the 1990s (1, 39). However, as these countries started the study period with higher obesity prevalence levels, these trends resulted in the CEE having higher average PGLE levels than the West.   The pattern of stagnation observed in the CEE, could be explained by the decrease in energy supplies at the beginning of the 1990s in CEE (37) resulting from the dramatic economic and political changes in those countries (3, 36, 39) and

For peer review only - http://bmjopen.bmj.com/site/about/guidelines.xhtml

which in turn affected the socio-economic status of the population (SES). Among CEE women, the increase in obesity starting in the 1990s was smaller than it was in the previous period, and was smaller than it was among CEE men. The lower risk of obesity observed among women than among men with low socioeconomic status (SES) in low-income countries (40) may explain this difference.

In Western Europe, a stagnation in PGLE levels was observed among women in Denmark and Switzerland after 1995. This finding seems to be in line with studies reporting a levelling-off of mean BMI since the 1990s (41); and in specific sub-populations, such as adults with high SES in regions within Switzerland, Italy, France, and Finland (37). Although dietary and physical activity information is spreading equally across socioeconomic groups, those with higher SES have a greater ability and capacity to adopt a healthier dietary and physical activity pattern (42). In addition, it appears that higher SES women in particular are more health-conscious, have healthier food habits, and are more prone to follow nutritional recommendations (43) as they are under greater social pressure to be thin (44). Similarly, countries with higher income levels and lower levels of inequality (45), like Switzerland and Denmark, tend to have lower obesity levels, especially among women.

For peer review only

When we considered the impact of obesity on life expectancy in the 26 European countries, we found that without obesity, the increase in e0 between 1975 and 2012 would have been, on average, 0.78 years higher among men and 0.30 years higher among women. These figures account for approximately 10% of the average change in e0 between 1975 and 2012 among men, and 5% among women. It is therefore clear that the impact of obesity on changes in e0 should not be ignored. Moreover, the impact of obesity on life expectancy

For peer review only - http://bmjopen.bmj.com/site/about/guidelines.xhtml

levels and on life expectancy trends is likely to increase, as previous studies have also suggested (8). There are several indicators pointing in that direction, including evidence that obesity's impact is already substantially greater in the USA (13% among men and 15% among women) than elsewhere; obesity prevalence is increasing rapidly in most European countries (see Supplementary Material, Figure S3); obesity is increasing in severity; and the duration of obesity is rising in younger generations (8).

Conclusion and implications

Obesity was proven to have an impact on both life expectancy levels and trends in Europe. The observed differences in the increase in the impact of obesity across countries and between the sexes reflect differences in the onset and the progression of the obesity epidemic, and can be linked to contextual factors (economic conditions, obesogenic environment, energy supplies), as well as to differences in people's ability and capacity to adopt healthier lifestyles.

It is likely that in the future obesity will have a larger impact on mortality and life expectancy in Europe, as obesity prevalence and obesity-attributable mortality continue to increase in the majority of countries. These trends will have important health, economic, and social implications. Specifically, the increasing prevalence of obesity among European populations, and especially at younger ages, will lead to an increased prevalence of obesity-related disorders, as well as to increases in the mortality burden associated with obesity and in obesity's effects on life expectancy and quality of life. Thus, obesity will constitute an additional burden for societies, economies, and public health. It is therefore crucial that effective public health initiatives are undertaken to tackle the obesity epidemic and its effects on public health. Such initiatives should address the multifactorial and complex

For peer review only - http://bmjopen.bmj.com/site/about/guidelines.xhtml

obesity aetiology; the clear differences between countries and the sexes; as well as the factors underlying these differences, such as contextual factors and differences in individuals' ability and capacity to adopt healthier lifestyles.

Funding

This work was supported by the Netherlands Organisation for Scientific Research (NWO) in connection with the research programme "Smoking, alcohol, and obesity, ingredients for improved and robust mortality projections, grant no. 452-13-001; see www.futuremortality.com.

For peer review only

Conflict of Interest

None declared.

Author contribution

N.Vidra contributed to the conception and design of the work; to the acquisition, analysis, and interpretation of data for the work; drafted and revised the work critically for important intellectual content; approved the final version of the work to be published and agrees to be accountable for all aspects of the work in ensuring that questions related to the accuracy or integrity of any part of the work are appropriately investigated and resolved.

S. Trias-Llimós contributed to the analysis, and interpretation of data for the work; revised it critically for important intellectual content; approved the final version of the work to be

For peer review only - http://bmjopen.bmj.com/site/about/guidelines.xhtml

published and agrees to be accountable for all aspects of the work in ensuring that questions related to the accuracy or integrity of any part of the work are appropriately investigated and resolved.

F. Janssen contributed to the conception and design of the work; to the interpretation of data for the work; revised the work critically for important intellectual content; approved the final version of the work to be published and agrees to be accountable for all aspects of the work in ensuring that questions related to the accuracy or integrity of any part of the work are appropriately investigated and resolved.

Data statement

Obesity prevalence data can be obtained from NCD Risk Factor Collaboration (NCD-RisC) (www.ncdrisc.org).

All-cause mortality and exposure population data can be obtained from the Human Mortality Database (http://www.mortality.org).

Acknowledgements

The authors are grateful to Professor Majid Ezatti and Dr James Bentham (Faculty of Medicine, School of Public Health at Imperial College London); Dr Gretchen A. Stevens (World Health Organization); and the NCD Risk Factor Collaboration (NCD-RisC) (www.ncdrisc.org) for sharing the obesity data.

This work is financed by the Netherlands Organisation for Scientific Research (NWO) as part of the research programme “Smoking, alcohol, and obesity, ingredients for improved and robust mortality projections, grant no. 452-13-001. See www.futuremortality.com

For peer review only

References

1. Finucane MM, Stevens GA, Cowan MJ, Danaei G, Lin JK, Paciorek CJ, et al. National, regional, and global trends in body-mass index since 1980: systematic analysis of health examination surveys and epidemiological studies with 960 country-years and 9.1 million participants. Lancet. 2011 Feb 12;377(9765):557-67.

2. Overweight and obesity - BMI statistics [Internet].; 2017 []. Available from: http://ec.europa.eu/eurostat/statistics-explained/index.php/Overweight_and_obesity_-_BMI_statistics.

3. WHO. The challenge of Obesity in the WHO European Region and the Strategies for Response: Summary. Copenhagen: World Health Organization, Regional Office for Europe; 2007.

4. Seidell JC. Prevalence and time trends of obesity in Europe. Journal of endocrinological investigation. 2002;25(10):816-22.

5. Eurostat. European Health Interview Survey - Almost 1 adult in 6 in the EU is considered obese - Share of obesity increases with age and decreases with education level. 2016.

6. Field AE, Coakley EH, Must A, Spadano JL, Laird N, Dietz WH, et al. Impact of overweight on the risk of developing common chronic diseases during a 10-year period. Archives of Internal Medicine. 2001 Jul 9;161(13):1581-6.

7. Global BMI Mortality Collaboration, Di Angelantonio E, Bhupathiraju S, Wormser D, Gao P, Kaptoge S, et al. Body-mass index and all-cause mortality: individual-participant-data meta-analysis of 239 prospective studies in four continents. Lancet. 2016 Aug 20;388(10046):776-86.

For peer review only

For peer review only - http://bmjopen.bmj.com/site/about/guidelines.xhtml

8. Alley DE, Lloyd J, Shardell M. Can obesity account for cross-national differences in life expectancy trends? In: Crimmins EM, Preston SH, Cohen B, editors. International differences in mortality at older ages: Dimensions and sources, panel on understanding divergent trends in longevity in high-income countries. Washington, DC: National Academies Press; 2011. p. 164-92.

9. Olshansky SJ, Passaro DJ, Hershow RC, Layden J, Carnes BA, Brody J, et al. A potential decline in life expectancy in the United States in the 21st century. The New England Journal of Medicine. 2005 Mar 17;352(11):1138-45.

10. Preston SH, Stokes A. Contribution of obesity to international differences in life expectancy. American Journal of Public Health. 2011 Nov;101(11):2137-43.

11. National Research Council. The Role of Obesity. In: Crimmins EM, Preston SH, Cohen B, editors. Explaining Divergent Levels of Longevity in High-Income Countries. Washington, DC: National Academies Press; 2011. p. 43-55.

12. Leon DA. Trends in European life expectancy: a salutary view. International Journal of Epidemiology. 2011 Apr;40(2):271-7.

13. NCD Risk Factor Collaboration. Trends in adult body-mass index in 200 countries from 1975 to 2014: a pooled analysis of 1698 population-based measurement studies with 19.2 million participants. Lancet. 2016 Apr 2;387(10026):1377-96.

14. Lobstein T, Leach RJ. Workpackage 7: Overweight and Obesity Report on data collection for overweight and obesity prevalence and related relative risks. 2010.

15. Allison DB, Fontaine KR, Manson JE, Stevens J, VanItallie TB. Annual deaths attributable to obesity in the United States. JAMA. 1999 Oct 27;282(16):1530-8.

16. Banegas JR, Lopez-Garcia E, Gutierrez-Fisac JL, Guallar-Castillon P, Rodriguez-Artalejo F. A simple estimate of mortality attributable to excess weight in the European Union. Eur J Clin Nutr. 2003 Feb;57(2):201-8.

For peer review only

For peer review only - http://bmjopen.bmj.com/site/about/guidelines.xhtml

472 17. A Robertson, T Lobstein, C Knai. Obesity and socio-economic groups in Europe: Evidence review and implications for action. 2007.

474 18. Flegal KM, Graubard BI, Williamson DF, Gail MH. Excess deaths associated with underweight, overweight, and obesity. JAMA. 2005 Apr 20;293(15):1861-7.

476 19. Katzmarzyk PT, Ardern CI. Overweight and obesity mortality trends in Canada, 1985-2000. Can J Public Health. 2004 Jan-Feb;95(1):16-20.

478 20. GBD 2017 Risk Factor Collaborators. Global, regional, and national comparative risk assessment of 84 behavioural, environmental and occupational, and metabolic risks or clusters of risks for 195 countries and territories, 1990-2017: a systematic analysis for the Global Burden of Disease Study 2017. Lancet. 2018 Nov 10;392(10159):1923-94.

482 21. University of California, Berkeley (USA), and Max Planck Institute for Demographic Research (Germany). [Internet]. []. Available from: http://www.mortality.org.

484 22. Barbieri M, Wilmoth JR, Shkolnikov VM, Glei D, Jasilionis D, Jdanov D, et al. Data Resource Profile: The Human Mortality Database (HMD). Int J Epidemiol. 2015 Oct;44(5):1549-56.

487 23. Cleveland WS, Loader C. Smoothing by local regression: Principles and methods. In: Murray Hill: A&T Bell Laboratorie; 1995.

489 24. Rockhill B, Newman B, Weinberg C. Use and misuse of population attributable fractions. American Journal of Public Health. 1998 Jan;88(1):15-9.

491 25. Preston SH, Heuveline P, Guillot M. Demography. Malden, MA: Blackwell; 2001.

492 26. Census data [Internet].; 2011 []. Available from: https://ec.europa.eu/CensusHub2/query.do?step=selectHyperCube&qhc=false.

494 27. Flegal KM, Panagiotou OA, Graubard BI. Estimating population attributable fractions to quantify the health burden of obesity. Annals of Epidemiology. 2015 Mar;25(3):201-7.

For peer review only

28. Mehta NK, Chang VW. Secular declines in the association between obesity and mortality in the United States. Population and Development Review. 2011;37(3):435-51.

29. Yu Y. Reexamining the declining effect of age on mortality differentials associated with excess body mass: evidence of cohort distortions in the United States. American Journal of Public Health. 2012 May;102(5):915-22.

30. Yu Y. The Changing Body Mass-Mortality Association in the United States: Evidence of Sex-Specific Cohort Trends from Three National Health and Nutrition Examination Surveys. Biodemography and Social Biology. 2016;62(2):143-63.

31. Vidra N, Bijlsma MJ, Janssen F. Impact of Different Estimation Methods on Obesity-Attributable Mortality Levels and Trends: The Case of The Netherlands. Int J Environ Res Public Health. 2018 Sep 29;15(10):10.3390/ijerph15102146.

32. Trias-Llimós S, Kunst AE, Jasilionis D, Janssen F. The contribution of alcohol to the East-West life expectancy gap in Europe from 1990 onward. International Journal of Epidemiology. 2017;47(3):731-9.

33. Doak CM, Wijnhoven TM, Schokker DF, Visscher TL, Seidell JC. Age standardization in mapping adult overweight and obesity trends in the WHO European Region. Obesity Reviews. 2012 Feb;13(2):174-91.

34. Cutler DM, Glaeser EL, Shapiro JM. Why Have Americans Become More Obese? The Journal of Economic Perspectives. 2003;17(1):93-118.

35. Obesity update 2012 [Internet].; 2012 []. Available from: http://www.oecd.org/els/health-systems/49716427.pdf.

36. Malik VS, Willett WC, Hu FB. Global obesity: trends, risk factors and policy implications. Nature reviews Endocrinology. 2013 Jan;9(1):13-27.

37. Silventoinen K, Sans S, Tolonen H, Monterde D, Kuulasmaa K, Kesteloot H, et al. Trends in obesity and energy supply in the WHO MONICA Project. International Journal of Obesity and Related Metabolic Disorders. 2004 May;28(5):710-8.

For peer review only

38. Zatonski WA, Bhala N. Changing trends of diseases in Eastern Europe: closing the gap. Public Health. 2012 Mar;126(3):248-52.

39. Bray GA, Bouchard C. Handbook of Obesity: Etiology and Pathophysiology. second ed. Boca Raton: CRC Press Taylor & Francis Group; 2003.

40. Monteiro CA, Conde WL, Lu B, Popkin BM. Obesity and inequities in health in the developing world. International Journal of Obesity. 2004 Sep;28(9):1181-6.

41. Wilkins E, Wilson L, Wickramasinghe K, Bhatnagar P, Leal J, Luengo-Fernandez R, et al. European Cardiovascular Disease Statistics 2017. Brussels: European Heart Network; 2017.

42. Robertson A, Lobstein T, Knai C. Obesity and socio-economic groups in Europe: Evidence review and implications for action. Brussels: European Commission. 2007.

43. Fagerli RA, Wandel M. Gender differences in opinions and practices with regard to a "healthy diet". Appetite. 1999 Apr;32(2):171-90.

44. Psaltopoulou T, Hatzis G, Papageorgiou N, Androulakis E, Briasoulis A, Tousoulis D. Socioeconomic status and risk factors for cardiovascular disease: Impact of dietary mediators. Hellenic Journal of Cardiology. 2017 Jan - Feb;58(1):32-42.

45. WHO. Obesity and inequities. Guidance for addressing inequities in overweight and obesity. Copenhagen: World Health Organization, Regional Office for Europe; 2014.

For peer review only

For peer review only - http://bmjopen.bmj.com/site/about/guidelines.xhtml

Figure legends

Figure 1: Age-standardised obesity-attributable mortality fractions in 26 European countries* (by 5 regions) and USA, 1975-2014, 18-100 years

* Countries within the same region are presented with the same colour

Central Europe: Czech Republic, Hungary, Poland, Slovakia

Eastern Europe: Belarus, Estonia, Ukraine, Latvia, Lithuania, Russian Federation

Northern Europe: Denmark, Finland, Iceland, Norway, Sweden

Southern Europe: Italy, Portugal, Spain

Western Europe: Austria, Belgium, France, Ireland, Luxembourg, Netherlands, Switzerland, United Kingdom

USA: United States of America

Figure 2: Potential gains in life expectancy at birth (PGLE) if obesity-attributable mortality was eliminated, in 26 European countries* (differentiating Western and Central Eastern Europe) and the USA, 1975-2012, 18-100 years

* Countries within the same region are presented with the same colour

Central Eastern Europe: Belarus, Czech Republic, Estonia, Hungary, Latvia, Lithuania, Poland, Russian Federation, Slovakia, Ukraine

Western Europe: Austria, Belgium, Denmark, Finland, France, Iceland, Ireland, Italy, Luxembourg, Netherlands, Norway, Portugal, Spain, Sweden, Switzerland, United Kingdom

USA: United States of America

Figure 3: Average annual increase (%) in potential gains in life expectancy due to obesity in 26 European countries and the USA between 1975-2012, in men

Figure 4: Average annual increase (%) in potential gains in life expectancy due to obesity in 26 European countries and the USA between 1975-2012, in women

For peer review only - http://bmjopen.bmj.com/site/about/guidelines.xhtml

For peer review only

For peer review only

For peer review only - http://bmjopen.bmj.com/site/about/guidelines.xhtml

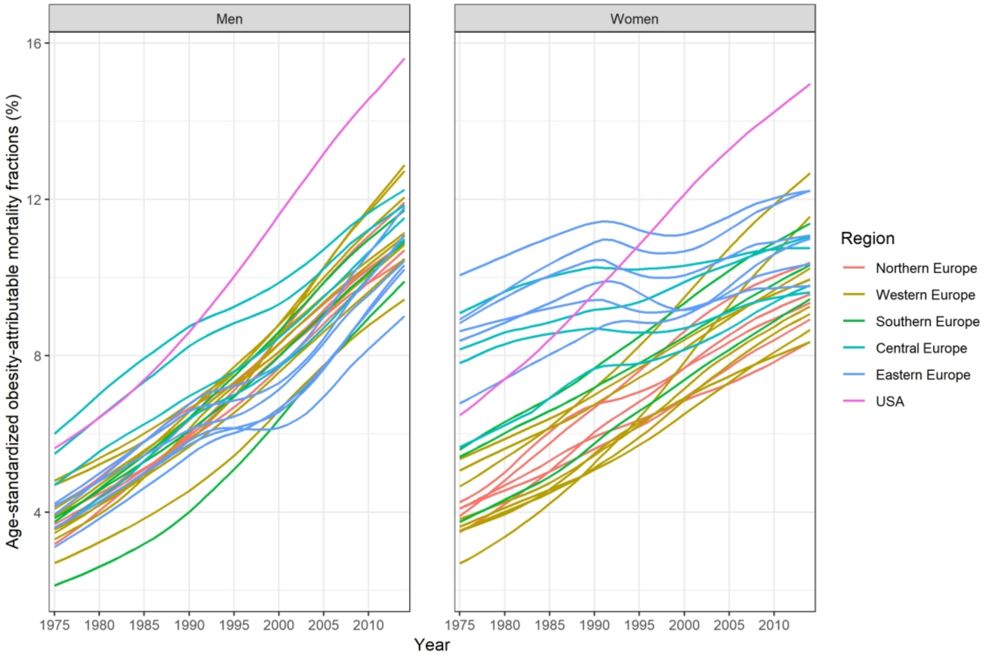

Figure 1: Age-standardised obesity-attributable mortality fractions in 26 European countries* (by 5 regions) and USA, 1975-2014, 18-100 years
* Countries within the same region are presented with the same colour
Central Europe: Czech Republic, Hungary, Poland, Slovakia
Eastern Europe: Belarus, Estonia, Ukraine, Latvia, Lithuania, Russian Federation
Northern Europe: Denmark, Finland, Iceland, Norway, Sweden
Southern Europe: Italy, Portugal, Spain
Western Europe: Austria, Belgium, France, Ireland, Luxembourg, Netherlands, Switzerland, United Kingdom
USA: United States of America

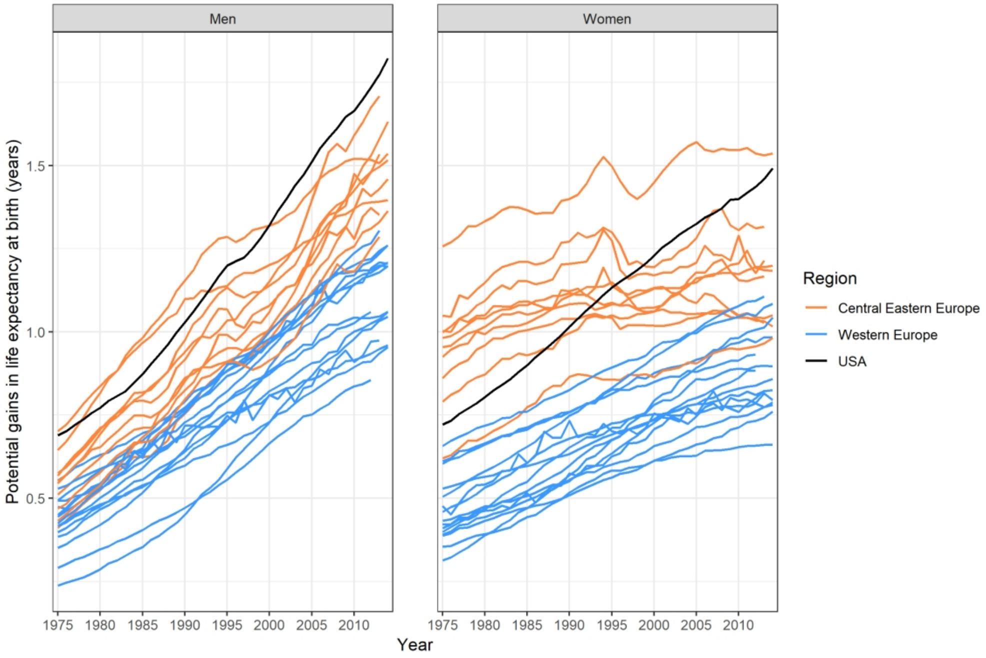

Figure 2: Potential gains in life expectancy at birth (PGLE) if obesity-attributable mortality was eliminated, in 26 European countries* (differentiating Western and Central Eastern Europe) and the USA, 1975-2012
* Countries within the same region are presented with the same colour
Central Eastern Europe: Belarus, Czech Republic, Estonia, Hungary, Latvia, Lithuania, Poland, Russian Federation, Slovakia, Ukraine
Western Europe: Austria, Belgium, Denmark, Finland, France, Iceland, Ireland, Italy, Luxembourg, Netherlands, Norway, Portugal, Spain, Sweden, Switzerland, United Kingdom
USA: United States of America

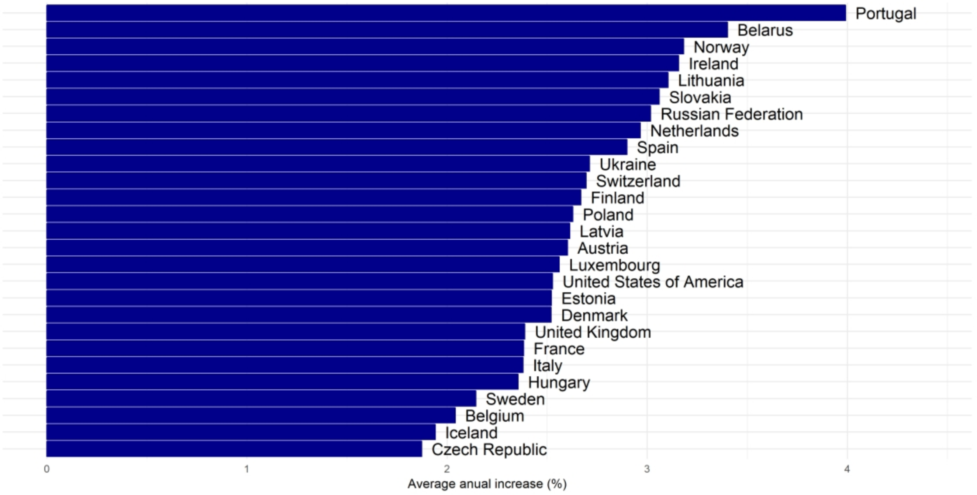

Figure 3: Average annual increase (%) in potential gains in life expectancy due to obesity in 26 European countries and the USA between 1975-2012, in men

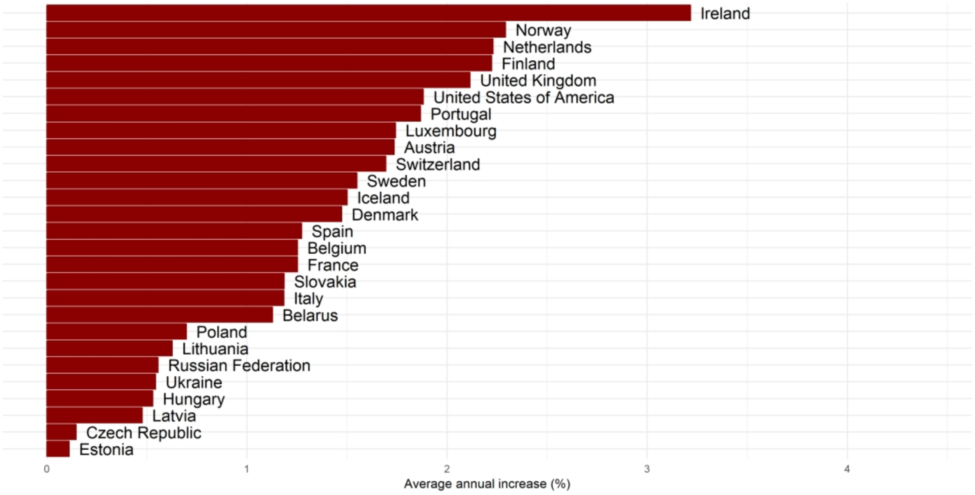

Figure 4: Average annual increase (%) in potential gains in life expectancy due to obesity in 26 European countries and the USA between 1975-2012, in women

Impact of obesity on life expectancy among different European countries, 1975-2012

Nikoletta Vidra[1], Sergi Trias-Llimós[1], Fanny Janssen[1,2]

[1] Population Research Centre, Faculty of Spatial Sciences, University of Groningen, Landleven 1, 9747 AD Groningen, The Netherlands.

[2] Netherlands Interdisciplinary Demographic Institute, Lange Houtstraat 19, 2511 CV The Hague, The Netherlands

For peer review only

Supplementary material

Table S1: Age-and sex-specific RRs of dying from obesity from the meta-review from Lobstein et al. (2010)

**Age**	**RR***	
	Men	***Women***
**<50**	1.55	1.5
**50-59**	1.539	1.49
**60-69**	1.5225	1.475
**70+**	1.495	1.45

*Reference group for the RRs: normal weight (18.5≥BMI≤24.9 kg/m^2)

For peer review only - http://bmjopen.bmj.com/site/about/guidelines.xhtml

Table S2: Potential gains in life expectancy at birth (PGLE) if obesity-attributable mortality was eliminated, in 26 European countries (differentiating Western and Central Eastern Europe) and the USA, in 1975 and 2012, 18-100 years

	**PGLE 1975**		**PGLE 2012**	
**Country**	***Men***	***Women***	***Men***	***Women***
Central Eastern Europe (CEE)				
Belarus	0.41	0.79	1.41	1.19
Czech Republic	0.70	0.98	1.39	1.03
Estonia	0.55	1.00	1.37	1.04
Hungary	0.64	0.86	1.52	1.04
Latvia	0.58	1.00	1.48	1.18
Lithuania	0.54	1.05	1.67	1.31
Poland	0.57	0.93	1.48	1.19
Russian Federation	0.51	1.26	1.53	1.54
Slovakia	0.43	0.62	1.31	0.96
Ukraine	0.47	0.95	1.25	1.16
Average CEE	0.54	0.94	1.44	1.16
Western Europe				
Austria	0.40	0.39	1.03	0.73
Belgium	0.55	0.61	1.17	0.97
Denmark	0.42	0.46	1.04	0.79
France	0.49	0.53	1.18	0.84
Finland	0.45	0.40	1.19	0.90
Ireland	0.38	0.31	1.21	1.01
Iceland	0.49	0.48	0.97	0.80
Italy	0.44	0.60	1.06	0.93
Luxembourg	0.47	0.42	1.19	0.79
Netherlands	0.29	0.39	0.86	0.88
Norway	0.34	0.39	1.07	0.91
Portugal	0.24	0.41	1.01	0.81
Spain	0.42	0.66	1.22	1.05
Sweden	0.42	0.43	0.91	0.76
Switzerland	0.35	0.35	0.93	0.66
United Kingdom	0.53	0.50	1.27	1.09
**Average Western Europe**	0.41	0.48	1.08	0.86
USA	0.69	0.72	1.73	1.44
**Average European countries**	0.46	0.64	1.22	0.98
**Average all countries**	0.47	0.64	1.23	1.00

For peer review only

Figure S1: Age-standardised obesity-attributable mortality fractions in 26 European countries*, grouped by 5 regions and USA, 1975-2014, 18-100 years

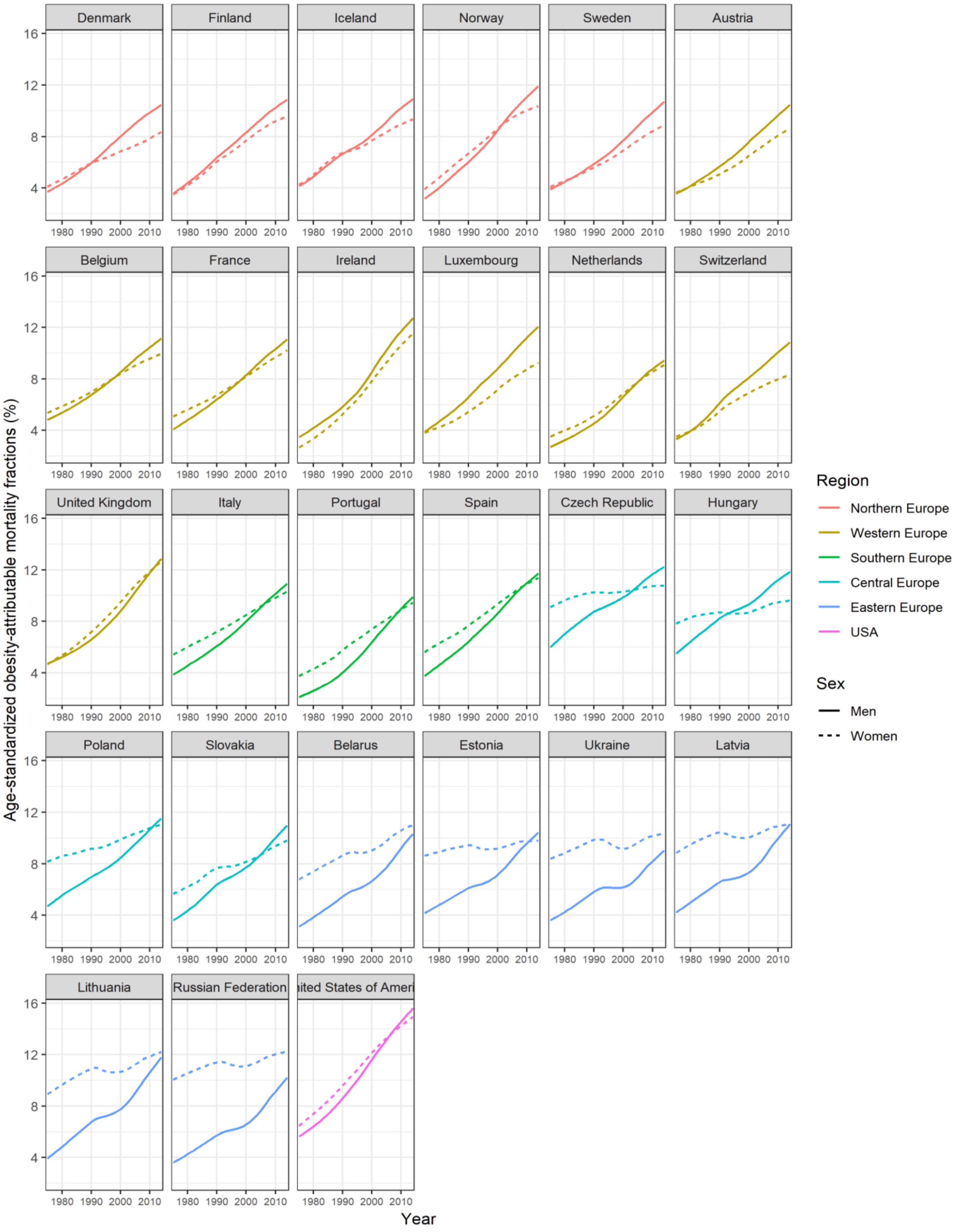

* Countries within the same region are presented with the same colour

Figure S2: Potential gains in life expectancy at birth (PGLE) if obesity-attributable mortality was eliminated, in 26 European countries*, grouped by 5 regions and USA, 1975-2012, 18-100 years

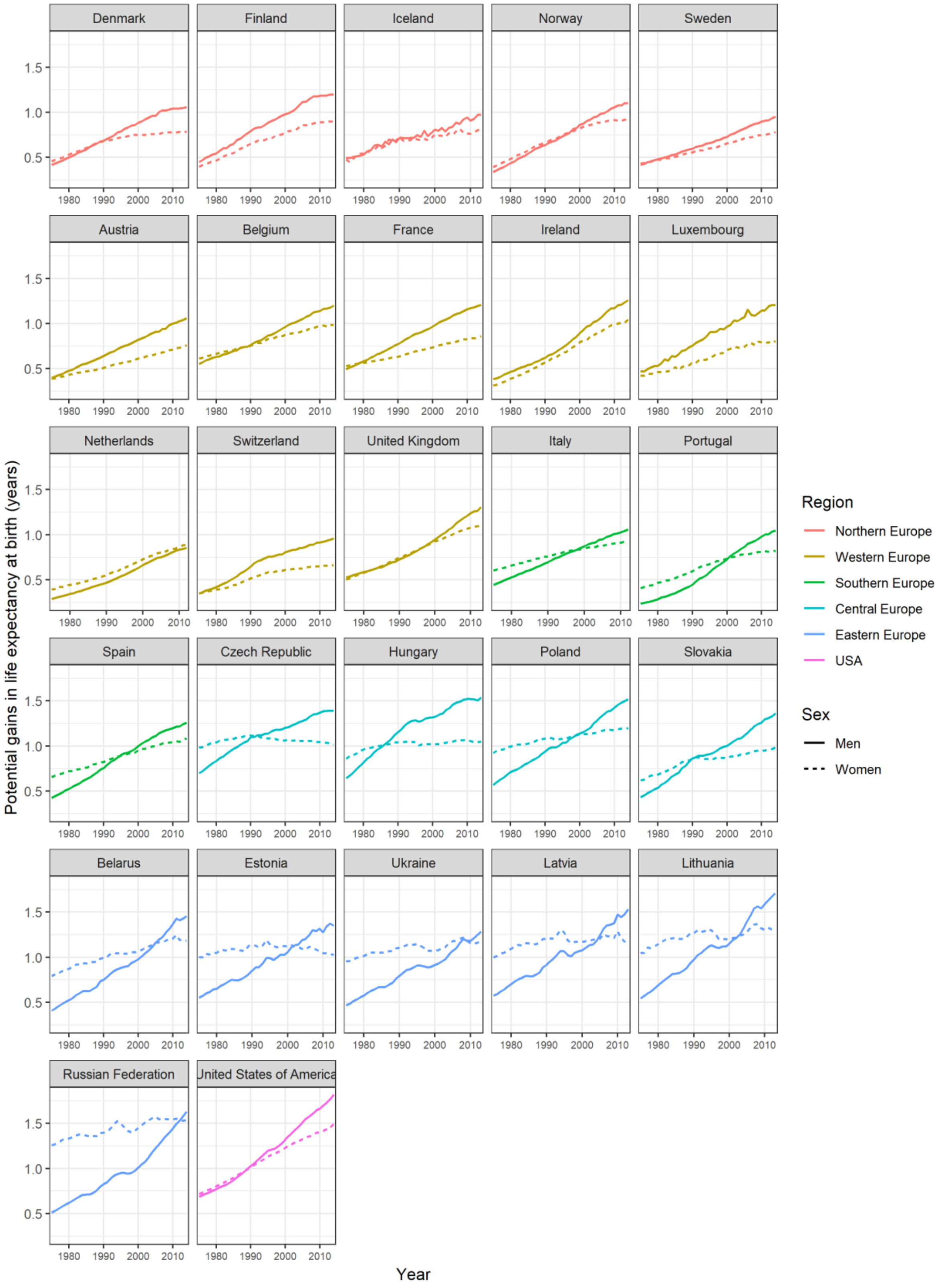

* Countries within the same region are presented with the same colour

Table S3: Age-standardised obesity prevalence and 95% confidence intervals, in 26 European countries (differentiating Western and Central Eastern Europe) and USA, 18-100 years in 2012.

	Age-standardised (stand.) obesity prevalence (%)			
**Country**	**Age-stand. Men**	**95% confidence intervals**	**Age-stand. Women**	**95% confidence intervals**
Central Eastern Europe				
Belarus	20.4	13.6; 28.5	25.4	17.7; 34.1
Czech Republic	25.6	18.9; 33.4	25.2	18.3; 33.0
Estonia	21.0	16.0; 26.8	22.7	17.4; 28.8
Hungary	24.6	17.9;32.0	22.1	15.4; 29.8
Latvia	22.2	15.2; 30.2	25.8	18.4; 34.5
Lithuania	23.8	16.9; 31.8	28.7	21.2; 37.1
Poland	23.6	18.0; 29.7	25.7	19.5; 32.5
Russian Federation	20.4	14.8; 26.8	29.1	22.6; 36.2
Slovakia	22.2	15.7; 29.6	22.2	15.8; 29.6
Ukraine	17.7	11.2; 25.7	23.9	16.3; 32.5
Western Europe				
Austria	21.1	14.7; 28.2	19.1	13.4; 25.5
Belgium	22.9	17.3; 29.1	22.7	17.1; 28.9
Denmark	21.3	15.5; 27.9	18.4	13.1; 24.5
France	22.6	16.3; 29.6	23.1	16.9; 29.9
Finland	22.3	17.1; 28.2	21.7	16.7; 27.3
Ireland	26.2	19.4; 33.8	26.0	19.5; 33.2
Iceland	22.3	15.6; 29.8	21.0	14.7; 28.3
Italy	22.3	17.0; 28.1	23.5	18.1; 29.6
Luxembourg	24.9	17.6; 32.9	20.7	14.3; 28.0
Netherlands	18.9	13.8; 24.5	20.3	15.3; 25.8
Norway	24.5	18.5; 31.2	23.7	18.0; 30.2
Portugal	19.6	13.8; 26.4	21.1	15.0; 28.1
Spain	24.2	18.5; 30.5	26.4	20.3; 32.8
Sweden	21.6	16.3; 27.4	19.8	14.7; 25.6
Switzerland	22.0	16.3; 28.3	18.6	13.1; 24.8
United Kingdom	26.5	22.3; 31.2	29.1	24.8; 33.6
USA	33.4	27.5; 39.5	35.5	29.7; 41.5

For peer review only - http://bmjopen.bmj.com/site/about/guidelines.xhtml

For peer review only

Table S4: Potential gains in life expectancy at age 50 (PGLE) if obesity-attributable mortality was eliminated, own estimates and those by Preston et al.2011, in the same countries studied, in 2006

Country	PGLE e50 2006, own estimates	PGLE e50 2006, Preston's estimates	Difference
Men			
Austria	0.81	1.00	-0.19
Belgium	0.95	0.98	-0.03
Czech Republic	1.14	1.34	-0.20
Denmark	0.88	0.82	0.06
France	0.94	0.99	-0.05
Italy	0.88	0.90	-0.02
Netherlands	0.69	0.73	-0.04
Poland	1.14	1.37	-0.23
Spain	1.02	1.15	-0.13
Sweden	0.75	0.72	0.03
Switzerland	0.77	0.79	-0.02
United Kingdom	0.99	1.34	-0.35
USA	1.29	1.85	-0.56
Women			
Austria	0.62	0.71	-0.09
Belgium	0.86	0.73	0.13
Czech Republic	0.87	1.01	-0.14
Denmark	0.71	0.62	0.09
France	0.72	0.52	0.2
Italy	0.84	0.57	0.27
Netherlands	0.76	0.69	0.07
Poland	1.08	1.19	-0.11
Spain	0.95	0.87	0.08
Sweden	0.67	0.63	0.04
Switzerland	0.59	0.50	0.09
United Kingdom	0.94	1.23	-0.29
USA	1.18	1.28	-0.10

For peer review only

For peer review only - http://bmjopen.bmj.com/site/about/guidelines.xhtml

Figure S3: Age-standardised obesity prevalence in 26 European countries*, grouped by 5 regions and USA, 1975-2012, 18-100 years

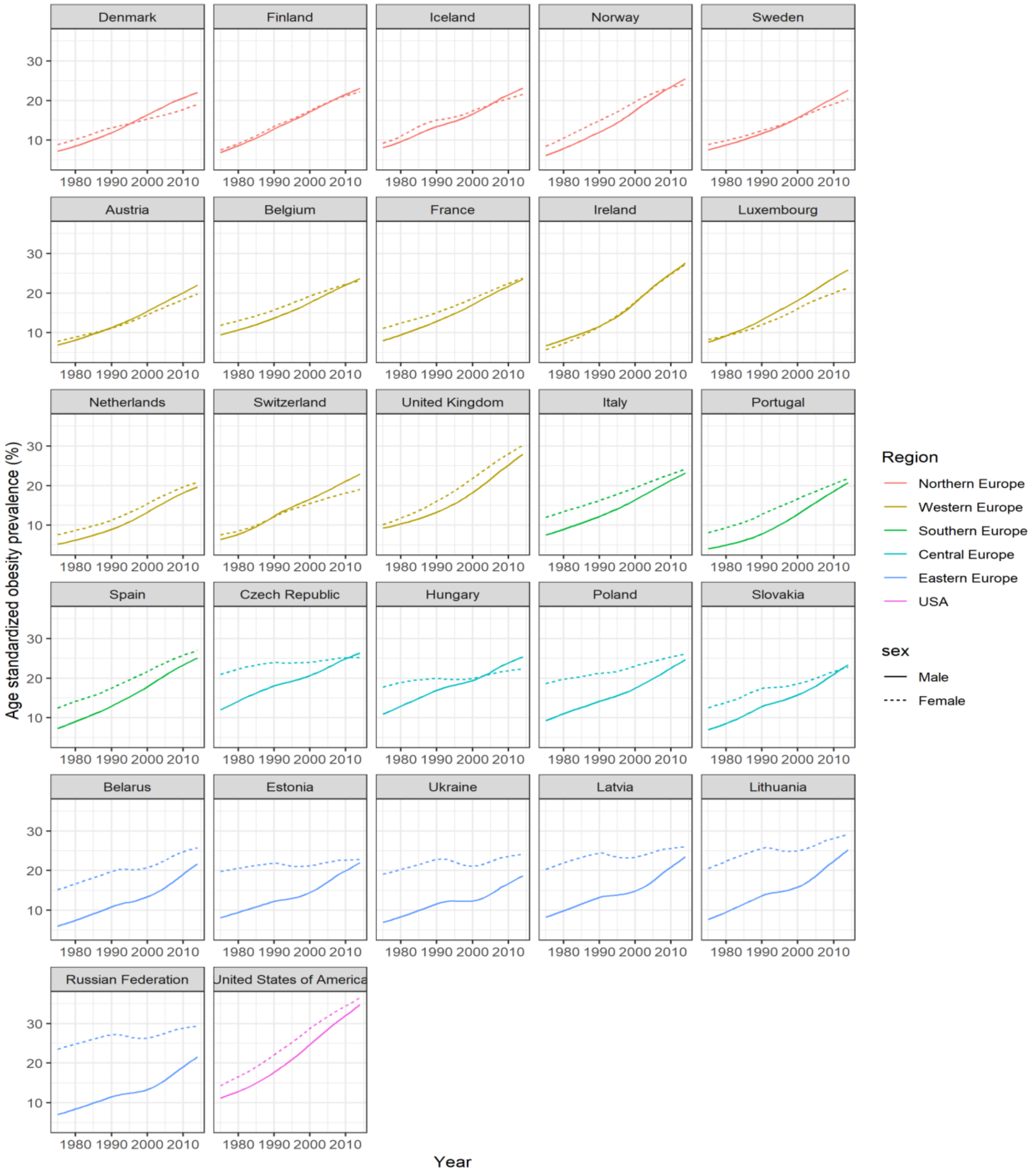

* Countries within the same region are presented with the same colour

BMJ Open

Impact of obesity on life expectancy among different European countries: secondary analysis of population-level data over the 1975-2012 period

Journal:	*BMJ Open*
Manuscript ID	bmjopen-2018-028086.R2
Article Type:	Research
Date Submitted by the Author:	25-Jun-2019
Complete List of Authors:	Vidra, Nikoletta; Population Research Centre, Faculty of Spatial Sciences, Demography<br>Trias-Llimós, Sergi; University of Groningen, Population Research Centre, Faculty of Spatial Sciences; London School of Hygiene and Tropical Medicine, Faculty of Epidemiology and Population Health<br>Janssen, Fanny; University of Groningen, Population Research Centre; Nederlands Interdisciplinair Demografisch Instituut
**Primary Subject Heading**:	Epidemiology
Secondary Subject Heading:	Public health, Nutrition and metabolism
Keywords:	EPIDEMIOLOGY, NUTRITION & DIETETICS, PUBLIC HEALTH

SCHOLARONE™
Manuscripts

Impact of obesity on life expectancy among different European countries: secondary analysis of population-level data over the 1975-2012 period

Nikoletta Vidra[1], Sergi Trias-Llimós[1], Fanny Janssen[1,2]

[1] Population Research Centre, Faculty of Spatial Sciences, University of Groningen, Landleven 1, 9747 AD Groningen, The Netherlands.

[2] Netherlands Interdisciplinary Demographic Institute, Lange Houtstraat 19, 2511 CV The Hague, The Netherlands.

Corresponding author: Nikoletta Vidra, Population Research Centre, Faculty of Spatial Sciences, University of Groningen, PO Box 800, 9700 AV Groningen, The Netherlands. Tel: 0031 (0) 50 363 86 52; e-mail: n.vidra@rug.nl

For peer review only

Word count: 3669 words

For peer review only - http://bmjopen.bmj.com/site/about/guidelines.xhtml

Abstract

Objective: This study assesses the impact of obesity on life expectancy for 26 European national populations and the USA over the 1975-2012 period.

Design: Secondary analysis of population-level obesity and mortality data.

Setting: European countries, namely Austria, Belarus, Belgium, the Czech Republic, Denmark, Estonia, Finland, France, Hungary, Iceland, Ireland, Italy, Latvia, Lithuania, Luxembourg, the Netherlands, Norway, Poland, Portugal, the Russian Federation, Slovakia, Spain, Sweden, Switzerland, Ukraine, the United Kingdom (UK); and the USA.

Participants: National populations aged 18–100 years, by sex.

Measurements: Using data by age and sex, we calculated obesity-attributable mortality by multiplying all-cause mortality (Human Mortality Database) with obesity-attributable mortality fractions (OAMFs). OAMFs were obtained by applying the weighted-sum method to obesity prevalence data (NCD Risk Factor Collaboration) and European Relative Risks (RRs) (DYNAMO). We estimated potential gains in life expectancy at birth (PGLE) by eliminating obesity-attributable mortality from all-cause mortality using associated single-decrement life tables.

Results: In the 26 European countries in 2012, PGLE due to obesity ranged from 0.86 to 1.67 years among men, and from 0.66 to 1.54 years among women. In all countries, PGLE increased over time, with an average annual increase of 2.68% among men and 1.33% and among women. Among women in Denmark, Switzerland, and Central and Eastern European countries, the increase in PGLE levelled off after 1995. Without obesity, the average increase

For peer review only - http://bmjopen.bmj.com/site/about/guidelines.xhtml

in life expectancy between 1975 and 2012 would have been 0.78 years higher among men and 0.30 years higher among women.

Conclusions: Obesity was proven to have an impact on both life expectancy levels and trends in Europe. The differences found in this impact between countries and the sexes can be linked to contextual factors, as well as to differences in people’s ability and capacity to adopt healthier lifestyles.

Keywords: Obesity, life expectancy, Europe, USA

Article Summary

Strengths and limitations of the study

- This is the first study to assess the impact of obesity on life expectancy at birth over time, and we do so here for 26 European countries and the USA.
- We used recent long-term comparable data on obesity prevalence based on population-based measurement studies, and European relative risks of dying from obesity by age and sex from a recent meta-analysis.
- Because of remaining data limitations regarding prevalence and relative risks, we had to use a fairly simple – albeit common applied - methodology to estimate obesity-attributable mortality.

For peer review only

Introduction

Obesity is a global epidemic (1), with Europe currently ranking second worldwide after the USA (2). Over the last 20 years obesity prevalence has increased threefold in Europe(3), although not uniformly across countries (4). Estimates for 2014 indicate that obesity varied threefold across European countries, ranging from a low of 9% in Romania to a high of 26% in Malta (5). Obesity constitutes a serious health burden at the individual and population levels because it is associated with an increased risk of morbidity (6), and mortality (7). However, the potential impact of the increase in obesity on life expectancy trends remains largely unknown (8).

The few existing studies that assessed the impact of obesity on life expectancy at the population level provided estimates at one specific point in time only (9, 10). Olshansky et al. found that if obesity was eliminated, life expectancy at birth (e0) in the USA in 2000 would be 0.21 to 1.08 years higher, depending on gender and ethnicity (9). Preston et al. (10) estimated for 16 low-mortality countries in 2006 that the reduction in life expectancy at age 50 (e50) due to obesity was greatest in the USA, at more than 1.5 years; and ranged from 0.50 to 1.19 years for women and from 0.72 to 1.37 years for men in European countries.

Gaining insight into the impact of obesity on trends in life expectancy is especially relevant (11) given the marked differences in life expectancy trends across Europe (12). In Western European countries, e0 has been increasing steadily, and has risen six to eight years since 1970. But in Central and Eastern Europe (CEE), e0 stagnated or even declined between the 1970s and the 1980s, and did not start increasing again until the 1990s. There are also marked differences in e0 trends between individual European countries (12).

For peer review only - http://bmjopen.bmj.com/site/about/guidelines.xhtml

In light of these important differences between European countries in both obesity prevalence and life expectancy over time, our aim is to assess the impact of obesity on long-term trends in life expectancy across a wide range of European countries.

Our sole focus is on the impact of obesity, given the significant health burden caused by obesity, the large body of literature on its impact, and the well-documented association of obesity with mortality.

Data and Methods

Setting

We studied the impact of obesity on life expectancy by sex over the 1975-2012 period in 26 European countries: Austria, Belarus, Belgium, the Czech Republic, Denmark, Estonia, Finland, France, Hungary, Iceland, Ireland, Italy, Latvia, Lithuania, Luxembourg, the Netherlands, Norway, Poland, Portugal, the Russian Federation, Slovakia, Spain, Sweden, Switzerland, Ukraine, the United Kingdom (UK); and the USA as a comparison country (10).

Data

Long-term comparable obesity prevalence data (BMI≥30kg/m2) by country, sex, age (18-19, 20-24, …, 85+), and year (1975-2012), based on 1698 population-based measurement studies, were obtained from the NCD Risk Factor Collaboration study (13). These validated data comprise the available measured height and weight data of 19.2 million participants from representative data sources, supplemented with estimates based on information from other years and related countries from a Bayesian hierarchical model (13). The same model was applied to all countries and used as an input the measured weight and height data, including covariates that help predict BMI (13).

For peer review only - http://bmjopen.bmj.com/site/about/guidelines.xhtml

The age- (<50, 50-59, 60-69 and ≥70 years) and sex-specific relative risks (RRs) of dying from obesity (see Supplementary Material Table S1) came from a review of studies mainly conducted in Western Europe and the USA, with the normal-weight category used as the reference group (18.5≥BMI≤25 kg/m^2) (14). These age- and sex-specific RRs were largely in line with the overall European RR of 1.64 recently estimated by the Global BMI Mortality Collaboration (7). The differences across age groups found in that study were similar with those reported in our findings (i.e., higher RRs at younger than at older ages), though they were less distinct (7). In addition, the use of RRs with the normal weight category as the reference category is in line with previous studies that estimated obesity-attributable mortality (15-19), while the estimation of obesity-attributable mortality with such a RR can be considered the theoretically maximally possible attributable mortality (20).

For peer review only

All-cause mortality numbers and exposure population data by single year of age, sex, year, and country were obtained from the Human Mortality Database (21). These data are of high quality and are widely used within the demographic community and beyond (22).

Patient and Public Involvement

No patients were involved in this study.

Methods

We performed our analyses separately by country and sex, based on data by single year of age (18-100). The obesity prevalence data were turned into single-age prevalence (18-100) by applying Loess smoothing (23). The RRs were turned into single-year RRs (18- 100) using linear regression.

For peer review only - http://bmjopen.bmj.com/site/about/guidelines.xhtml

To estimate the obesity-attributable mortality fraction (OAMF) – i.e., the share of all-cause mortality due to obesity – we used the Rockhill formula to estimate OAMFs by age (a) and sex (s) (24).

$$OAMF_{a,s} = \frac{P_{a,s} \cdot (RR_{a,s} - 1)}{1 + (P_{a,s} \cdot \; (RR_{a,s} - 1))} \text{(Equation 1)}$$

where *P* is the obesity prevalence. We then weighted the $OAMF_{a,s}$ with the corresponding number of deaths.

For the estimation of the impact of obesity on life expectancy (see 2.3.2) we needed age-and sex-specific (non-) obesity-attributable mortality rates. These were obtained by multiplying $OAMF_{a,s}$ and [1- $OAMF_{a,s}$], respectively, with age- and sex-specific all-cause mortality rates.

To ensure comparability across countries, over time, and between men and women, we applied direct age- and sex-standardisation (25) to obesity prevalence, obesity-attributable mortality fractions, and obesity-attributable mortality rates, using the European population of 2011 (26) as the standard.

To assess the impact of adult obesity on e_0, we calculated for each country the potential gain in life expectancy (PGLE) if obesity-attributable mortality were eliminated, by calendar year and sex and is in line with the approach by Preston et al. First, we calculated e_0 by applying standard life table techniques to age-specific all-cause mortality rates (0-100)(25)). Second, we applied associated single-decrement life tables (ASDLT)(25)) to age- and sex-specific non-obesity-attributable mortality rates (0-100) to obtain e_0 if obesity-attributable mortality were eliminated. The PGLE represents the difference between the e_0 based on the ASDLT and the original $e_{0.}$

For peer review only

For peer review only - http://bmjopen.bmj.com/site/about/guidelines.xhtml

To summarise the changes in PGLE across countries, we estimated the average annual changes in PGLE (in %):

$$Average\ annual\ changes\ in\ PGLE\ (\%) = \frac{\sum_{t=1976}^{2012} \frac{(PGLE_t - PGLE_{t-1})}{PGLE_{t-1}}}{2012 - 1975} 100$$

To assess the impact of obesity on time trends in e_0 between 1975 and 2012, we subtracted the observed change in e_0 from the change in e_0 without obesity. The change in e_0 without obesity was obtained by using the e_0 values from the associated single-decrement life tables applied to non-obesity-attributable mortality for 1975 and 2012.

Results

For the 26 European countries, the age-standardised obesity-attributable mortality fraction (OAMF) was, on average, 11% among men and 10% among women in 2012. For the USA, these estimates were substantially higher; i.e., 15% and 14%, respectively. The average OAMF levels were higher in Northern, Western, and Southern Europe combined (hereafter, Western Europe) than in CEE among men, while the opposite was the case among women.

OAMFs were increasing over time for all countries and both sexes, although not to the same extent (see Figure 1, Figure S1). In Western Europe, OAMFs generally increased over the 1975-2012 period, and at a faster pace among men. In CEE, by contrast, OAMFs clearly stagnated, and even declined between 1990 and 2000. The overall increase in OAMFs was greatest in the USA, Ireland, Norway (men), and the UK (women).

For peer review only - http://bmjopen.bmj.com/site/about/guidelines.xhtml

Figure 1: Age-standardised obesity-attributable mortality fractions in 26 European countries* (by 5 regions) and USA, 1975-2014, 18-100 years

* Countries within the same region are presented with the same colour

Central Europe: Czech Republic, Hungary, Poland, Slovakia

Eastern Europe: Belarus, Estonia, Ukraine, Latvia, Lithuania, Russian Federation

Northern Europe: Denmark, Finland, Iceland, Norway, Sweden

Southern Europe: Italy, Portugal, Spain

Western Europe: Austria, Belgium, France, Ireland, Luxembourg, Netherlands, Switzerland, United Kingdom

USA: United States of America

<approximately here>

In the 26 European countries in 2012, estimates of potential gains in life expectancy at birth (PGLE) if obesity was eliminated ranged from 0.86 to 1.67 years among men (1.22 on average) and from 0.66 to 1.54 years (0.98 on average) among women (Figure 2; Figure S2 and Table S2 Supplementary Material). Among men in the USA, the PGLE estimate was, at 1.73 years, slightly higher than the highest estimate in Europe; and among women in the USA, the PGLE estimate was, at 1.44 years, the second-highest after the estimate for Russia. The average PGLE estimate was 1.08 among men and 0.86 among women in Western Europe, and was 1.44 among men and 1.16 among women in CEE (see Supplementary Material, Table S2).

For peer review only

Figure 2: Potential gains in life expectancy at birth (PGLE) if obesity-attributable mortality was eliminated, in 26 European countries* (differentiating Western and Central Eastern Europe) and the USA, 1975-2012

* Countries within the same region are presented with the same colour
Central Eastern Europe: Belarus, Czech Republic, Estonia, Hungary, Latvia, Lithuania, Poland, Russian Federation, Slovakia, Ukraine
Western Europe: Austria, Belgium, Denmark, Finland, France, Iceland, Ireland, Italy, Luxembourg, Netherlands, Norway, Portugal, Spain, Sweden, Switzerland, United Kingdom
USA: United States of America

<approximately here>

Overall, from 1975 to 2012, PGLE due to obesity increased in all of the countries (Figure 2, Figure S2, Figure 3, 4). The increase was greater among men (average annual increase of 2.68%) than among women (average annual increase of 1.33%), was largest among men in Portugal and Belarus and among women in Portugal, and was substantial among men and women in Norway (Figures 3, 4). While there was a general increase in PGLE due to obesity, this trend stagnated among women in CEE from around 1990 onwards, and levelled off after 1995 among women in Denmark and Switzerland.

Figure 3: Average annual increase (%) in potential gains in life expectancy due to obesity in 26 European countries and the USA between 1975-2012, in men

<approximately here>

Figure 4: Average annual increase (%) in potential gains in life expectancy due to obesity in 26 European countries and the USA between 1975-2012, in women

<approximately here>

For peer review only
For peer review only - http://bmjopen.bmj.com/site/about/guidelines.xhtml

Table 1 shows the impact of obesity on time trends in life expectancy at birth (e_0). Overall, the average increase in e_0 between 1975 and 2012 was 7.26 years for men and 6.28 years for women in the 26 European countries. Without obesity, the average increase in e_0 would have been 8.04 years for men and 6.58 years for women; or 0.78 and 0.30 years higher, respectively. Among men, obesity had the greatest impact on e_0 trends in Lithuania and the USA (more than one year), and the smallest impact in Iceland and Sweden (0.5 years). Among women, obesity had the greatest impact on e_0 trends in the USA and Ireland (0.7 years) and the smallest impact in Estonia and the Czech Republic (less than 0.1 year).

Table 1: Impact of obesity on trends in life expectancy at birth (e_0) in 26 European countries and USA 1975-2012, by sex

Country	Change in e_0 with obesity 2012-1975 (years)		Change in e_0 without obesity 2012-1975 (years)		Effect of obesity on e_0 change 2012-1975 (years)	
	Men	*Women*	*Men*	*Women*	*Men*	*Women*
Austria	10.62	8.61	11.25	8.95	0.63	0.34
Belarus	-0.55	1.43	0.46	1.83	1.00	0.40
Belgium	8.85	7.63	9.46	7.99	0.61	0.36
Czech Republic	7.97	6.98	8.66	7.03	0.69	0.05
Denmark	6.78	5.03	7.40	5.36	0.63	0.33
Estonia	6.43	6.42	7.26	6.46	0.82	0.04
France	9.49	7.99	10.17	8.30	0.68	0.31
Finland	10.07	7.26	10.82	7.75	0.74	0.50
Hungary	5.29	6.18	6.17	6.36	0.87	0.18
Iceland	9.02	5.19	9.51	5.51	0.48	0.32
Ireland	9.40	8.40	10.22	9.10	0.83	0.69
Italy	10.19	8.56	10.81	8.89	0.62	0.33
Latvia	4.91	4.53	5.82	4.70	0.90	0.18
Lithuania	2.01	3.80	3.14	4.06	1.13	0.26
Luxembourg	11.78	9.27	12.50	9.65	0.72	0.37
Netherlands	7.68	5.10	8.26	5.6	0.56	0.49
Norway	7.70	5.33	8.42	5.86	0.74	0.51
Poland	5.90	6.74	6.81	7.00	0.91	0.27
Portugal	12.14	10.87	12.91	11.26	0.77	0.40

For peer review only

For peer review only - http://bmjopen.bmj.com/site/about/guidelines.xhtml

Russian Federation	2.05	2.62	3.06	2.89	1.02	0.28
Slovakia	5.65	5.82	6.52	6.16	0.88	0.34
Spain	8.82	8.75	9.62	9.14	0.79	0.39
Sweden	7.69	5.59	8.18	5.93	0.49	0.33
Switzerland	8.98	6.63	9.55	6.93	0.58	0.30
Ukraine	0.48	1.73	1.26	1.94	0.78	0.21
United Kingdom	9.46	6.96	10.20	7.55	0.74	0.59
USA	7.86	4.89	8.90	5.61	1.04	0.71
**Average CEE countries**	4.01	4.63	4.92	4.84	0.90	0.22
**Average Western countries**	9.10	7.09	9.76	7.50	0.66	0.41

For peer review only

Discussion

Summary of results

In the 26 European countries studied, the share of mortality due to obesity in 2012 was, on average, 11% among men and 10% among women. PGLE due to obesity in 2012 ranged from 0.86 to 1.73 years among men, and from 0.66 to 1.54 years among women. Overall, PGLE increased between 1975 and 2012, albeit more quickly among men (average annual increase: 2.68%) than among women (1.33%). Among women in Denmark, Switzerland, and the CEE countries the increase in PGLE levelled off after 1995. Without obesity, the average increase in e0 between 1975 and 2012 would have been 0.78 years higher among men and 0.30 years higher among women.

Evaluation of data and methods

Using the recent advances in obesity data, it is now possible to study the impact of obesity on life expectancy for a large number of countries and a long period of time. Two methodological issues warrant our attention, however.

For peer review only - http://bmjopen.bmj.com/site/about/guidelines.xhtml

First, in calculating the share of mortality due to obesity (OAMF), which also forms the basis for our PGLE calculations, we were hindered by limitations in the available prevalence and RRs data, which also affected the method used. As has previously been documented, OAMF estimates are sensitive to the data and the methods used (27).

In selecting obesity prevalence data, we used the longest validated time series based on population-based measurement studies that are suitable for studying the impact of obesity on long-term life expectancy trends across Europe (13). For those countries with less available obesity data – especially the CEE countries a portion of the data we used were merely the result of modelling. Thus, the resulting estimates should be treated with some caution . By contrast, for the non-CEE countries, most of the data we used pertain to measured data (13). Supplementary Material, Table S3 gives the confidence intervals around the age-standardised prevalence estimates for each country by sex in order, as to provide more information on the relative reliability of the data for the different countries in our analysis.

Because age- and sex-specific RRs of mortality associated with obesity are not readily available by country and year, we have decided to apply to all of the countries studied age- and sex-specific RRs from Western European and US populations that are largely suitable for our setting, as had previously been done (10). Although RRs could differ slightly across contexts, studies that compared RRs across continents found only small differences in RRs between Europe and North America (7). Consequently, we do not expect to observe large differences between individual countries. In addition, as time-variant European RRs were not available, we had to apply time-constant RRs, even though it is possible that changes in the association of obesity with mortality – which could, for example, occur because of improvements in the treatment of chronic diseases – have affected the impact of obesity on

For peer review only - http://bmjopen.bmj.com/site/about/guidelines.xhtml

life expectancy. Previous studies that assessed changes over time in the association of obesity with mortality did so only for the US, and, unfortunately, provided mixed evidence, with some of these studies reporting a decline (18, 28, 29), and others finding an increase (30).Therefore, before implementing time-variant European RRs, more information on their direction is required. Similarly, comparable country-specific RRs are urgently needed. In addition, the choice of these RRs along with their reference group might exert an effect in our estimates. Based on the available data, only a fairly simple – albeit common applied – the weighted sum method could be applied (27) to estimate the OAMFs. The application of a more advanced methodology (27) could have affected the OAMFs and thus the PGLE levels, but less the trends(31). The lack of information on the uncertainty of the RRs we used limited us in estimating confidence intervals for the OAMFs and PGLEs.

Second, besides being the result of the OAMFs, the PGLE estimates can also be affected by all-cause mortality levels and trends as age- and sex-specific all-cause mortality rates are used to estimate PGLE. Since all-cause mortality fluctuated greatly in CEE in the analysed period (12), short-term variations in PGLE in CEE countries should be treated with more caution.

Explanation of results

In 2012, the PGLE due to obesity were, on average, 1.22 years for men and 0.98 years for women in the 26 European countries, and 1.73 years for men and 1.43 years for women in the USA. A comparison of our 2006 e50 estimates with those of Preston et al. (10) for the same countries uncovered only small differences, except among men in the USA (our estimate was 0.56 years lower) and women in the UK (our estimate was 0.29 years lower) (see Supplementary Material, Table S4). Given that approximately the same methodology was used to estimate the OAMFs, the observed differences are most likely due to the use of

For peer review only - http://bmjopen.bmj.com/site/about/guidelines.xhtml

different obesity prevalence and RRs data. Preston used prevalence data from national representative surveys and RRs from the Prospective Studies collaboration (10). Given that the observed differences do not have the same direction for the different countries, we believe that these differences are mainly attributable to the prevalence data used. To further evaluate our observed PGLE levels, we compared them with own PGLE estimates for smoking and alcohol (32). Our PGLE estimates for smoking were 2.38 years for men and 1.00 year for women in Western Europe, and 3.82 years for men and 0.67 years for women in CEE. Our PGLE estimates for alcohol were 0.90 years for men and 0.44 years for women in Western Europe, and 2.15 years for men and 1.00 year for women in CEE (32) while our average PGLE for obesity was 1.08 among men and 0.86 among women in Western Europe, and 1.44 among men and 1.16 among women in CEE. Thus, obesity's impact on life expectancy lies between that of smoking and alcohol, and can be considered significant.

In our study, we found that PGLE due to obesity was increasing, but that this trend differed across countries and between the sexes. This overall trend can be explained by the general increase in obesity prevalence in European countries (see Supplementary Material, Figure S3) (13) and the resulting growth in the burden of obesity (3) , which is also reflected in the OAMFs (Figure 1, Figure S1) in these countries.

At the same time, parts of the observed variation in the increase in PGLE estimates across the USA, Western Europe, and CEE and between the sexes reflect differences in the onset, the development, and the impact of the obesity epidemic in these countries and in men and women. Across the countries studied, the absolute increase in PGLE was largest among women and second-largest among men in the USA. This pattern is in line with evidence showing that between 1980 and 2008, obesity increased much more in the USA

For peer review only - http://bmjopen.bmj.com/site/about/guidelines.xhtml

than in Europe(1, 33). This rapid progression of the obesity epidemic in the USA and its large impact on life expectancy has been attributed to an increasingly obesogenic environment caused by factors such as changes in food preparation and processes that promote the consumption of calorically dense foods, and a pronounced decrease in physical activity levels (34). The obesity epidemic has progressed more slowly in Western Europe than in the USA(1, 13). However, obesity levels in countries like the UK and Ireland are rapidly approaching those in the USA (35), as our PGLE estimates also show.

In the CEE countries, the PGLE trends track the evolution of the obesity epidemic in that region (see Supplementary Material, Figure S3). Obesity levels have been higher in CEE than in Western Europe since 1980 (36, 37), which suggests that the epidemic started earlier in CEE. As a result of this earlier onset, the impact of obesity (as expressed in terms of OAMF and PGLE) in the 1970s and 1980s was at times even greater in CEE than in the USA, especially among women. While there are many potential explanations for this early onset of the obesity epidemic in CEE, the available data indicate that the main factors were the relatively high total energy supply and energy intake in CEE in those years (38).

The overall progress of the obesity epidemic was lower in CEE than in Western Europe, and the increase was not constant (1). Indeed, in CEE, increases in obesity prevalence(1, 39), OAMFs, and PGLE stagnated in the 1980-2008 period, and were more pronounced in the 1990s (1, 39). However, as these countries started the study period with higher obesity prevalence levels, these trends resulted in the CEE having higher average PGLE levels than the West.   The pattern of stagnation observed in the CEE, could be explained by the decrease in energy supplies at the beginning of the 1990s in CEE (37) resulting from the dramatic economic and political changes in those countries (3, 36, 39) and

For peer review only - http://bmjopen.bmj.com/site/about/guidelines.xhtml

which in turn affected the socio-economic status of the population (SES). Among CEE women, the increase in obesity starting in the 1990s was smaller than it was in the previous period, and was smaller than it was among CEE men. The lower risk of obesity observed among women than among men with low socioeconomic status (SES) in low-income countries (40) may explain this difference.

In Western Europe, a stagnation in PGLE levels was observed among women in Denmark and Switzerland after 1995. This finding seems to be in line with studies reporting a levelling-off of mean BMI since the 1990s (41); and in specific sub-populations, such as adults with high SES in regions within Switzerland, Italy, France, and Finland (37). Although dietary and physical activity information is spreading equally across socioeconomic groups, those with higher SES have a greater ability and capacity to adopt a healthier dietary and physical activity pattern (42). In addition, it appears that higher SES women in particular are more health-conscious, have healthier food habits, and are more prone to follow nutritional recommendations (43) as they are under greater social pressure to be thin (44). Similarly, countries with higher income levels and lower levels of inequality (45), like Switzerland and Denmark, tend to have lower obesity levels, especially among women.

When we considered the impact of obesity on life expectancy in the 26 European countries, we found that without obesity, the increase in e0 between 1975 and 2012 would have been, on average, 0.78 years higher among men and 0.30 years higher among women. These figures account for approximately 10% of the average change in e0 between 1975 and 2012 among men, and 5% among women. It is therefore clear that the impact of obesity on changes in e0 should not be ignored. Moreover, the impact of obesity on life expectancy

For peer review only - http://bmjopen.bmj.com/site/about/guidelines.xhtml

levels and on life expectancy trends is likely to increase, as previous studies have also suggested (8). There are several indicators pointing in that direction, including evidence that obesity's impact is already substantially greater in the USA (13% among men and 15% among women) than elsewhere; obesity prevalence is increasing rapidly in most European countries (see Supplementary Material, Figure S3); obesity is increasing in severity; and the duration of obesity is rising in younger generations (8).

Conclusion and implications

Obesity was proven to have an impact on both life expectancy levels and trends in Europe. The observed differences in the increase in the impact of obesity across countries and between the sexes reflect differences in the onset and the progression of the obesity epidemic, and can be linked to contextual factors (economic conditions, obesogenic environment, energy supplies), as well as to differences in people's ability and capacity to adopt healthier lifestyles.

It is likely that in the future obesity will have a larger impact on mortality and life expectancy in Europe, as obesity prevalence and obesity-attributable mortality continue to increase in the majority of countries. These trends will have important health, economic, and social implications. Specifically, the increasing prevalence of obesity among European populations, and especially at younger ages, will lead to an increased prevalence of obesity-related disorders, as well as to increases in the mortality burden associated with obesity and in obesity's effects on life expectancy and quality of life. Thus, obesity will constitute an additional burden for societies, economies, and public health. It is therefore crucial that effective public health initiatives are undertaken to tackle the obesity epidemic and its effects on public health. Such initiatives should address the multifactorial and complex

For peer review only

obesity aetiology; the clear differences between countries and the sexes; as well as the factors underlying these differences, such as contextual factors and differences in individuals' ability and capacity to adopt healthier lifestyles.

Funding

This work was supported by the Netherlands Organisation for Scientific Research (NWO) in connection with the research programme "Smoking, alcohol, and obesity, ingredients for improved and robust mortality projections, grant no. 452-13-001; see www.futuremortality.com.

Conflict of Interest

None declared.

Author contribution

N.Vidra contributed to the conception and design of the work; to the acquisition, analysis, and interpretation of data for the work; drafted and revised the work critically for important intellectual content; approved the final version of the work to be published and agrees to be accountable for all aspects of the work in ensuring that questions related to the accuracy or integrity of any part of the work are appropriately investigated and resolved.

S. Trias-Llimós contributed to the analysis, and interpretation of data for the work; revised it critically for important intellectual content; approved the final version of the work to be

For peer review only

For peer review only - http://bmjopen.bmj.com/site/about/guidelines.xhtml

published and agrees to be accountable for all aspects of the work in ensuring that questions related to the accuracy or integrity of any part of the work are appropriately investigated and resolved.

F. Janssen contributed to the conception and design of the work; to the interpretation of data for the work; revised the work critically for important intellectual content; approved the final version of the work to be published and agrees to be accountable for all aspects of the work in ensuring that questions related to the accuracy or integrity of any part of the work are appropriately investigated and resolved.

For peer review only

Data statement

Obesity prevalence data can be obtained from NCD Risk Factor Collaboration (NCD-RisC) (www.ncdrisc.org).

All-cause mortality and exposure population data can be obtained from the Human Mortality Database (http://www.mortality.org).

Acknowledgements

The authors are grateful to Professor Majid Ezatti and Dr James Bentham (Faculty of Medicine, School of Public Health at Imperial College London); Dr Gretchen A. Stevens (World Health Organization); and the NCD Risk Factor Collaboration (NCD-RisC) (www.ncdrisc.org) for sharing the obesity data.

This work is financed by the Netherlands Organisation for Scientific Research (NWO) as part of the research programme “Smoking, alcohol, and obesity, ingredients for improved and robust mortality projections, grant no. 452-13-001. See www.futuremortality.com

For peer review only - http://bmjopen.bmj.com/site/about/guidelines.xhtml

References

1. Finucane MM, Stevens GA, Cowan MJ, Danaei G, Lin JK, Paciorek CJ, et al. National, regional, and global trends in body-mass index since 1980: systematic analysis of health examination surveys and epidemiological studies with 960 country-years and 9.1 million participants. Lancet. 2011 Feb 12;377(9765):557-67.

2. Overweight and obesity - BMI statistics [Internet].; 2017 []. Available from: http://ec.europa.eu/eurostat/statistics-explained/index.php/Overweight_and_obesity_-_BMI_statistics.

3. WHO. The challenge of Obesity in the WHO European Region and the Strategies for Response: Summary. Copenhagen: World Health Organization, Regional Office for Europe; 2007.

4. Seidell JC. Prevalence and time trends of obesity in Europe. Journal of endocrinological investigation. 2002;25(10):816-22.

5. Eurostat. European Health Interview Survey - Almost 1 adult in 6 in the EU is considered obese - Share of obesity increases with age and decreases with education level. 2016.

6. Field AE, Coakley EH, Must A, Spadano JL, Laird N, Dietz WH, et al. Impact of overweight on the risk of developing common chronic diseases during a 10-year period. Archives of Internal Medicine. 2001 Jul 9;161(13):1581-6.

7. Global BMI Mortality Collaboration, Di Angelantonio E, Bhupathiraju S, Wormser D, Gao P, Kaptoge S, et al. Body-mass index and all-cause mortality: individual-participant-data meta-analysis of 239 prospective studies in four continents. Lancet. 2016 Aug 20;388(10046):776-86.

For peer review only - http://bmjopen.bmj.com/site/about/guidelines.xhtml

448 8. Alley DE, Lloyd J, Shardell M. Can obesity account for cross-national differences in life expectancy trends? In: Crimmins EM, Preston SH, Cohen B, editors. International differences in mortality at older ages: Dimensions and sources, panel on understanding divergent trends in longevity in high-income countries. Washington, DC: National Academies Press; 2011. p. 164-92.

453 9. Olshansky SJ, Passaro DJ, Hershow RC, Layden J, Carnes BA, Brody J, et al. A potential decline in life expectancy in the United States in the 21st century. The New England Journal of Medicine. 2005 Mar 17;352(11):1138-45.

456 10. Preston SH, Stokes A. Contribution of obesity to international differences in life expectancy. American Journal of Public Health. 2011 Nov;101(11):2137-43.

458 11. National Research Council. The Role of Obesity. In: Crimmins EM, Preston SH, Cohen B, editors. Explaining Divergent Levels of Longevity in High-Income Countries. Washington, DC: National Academies Press; 2011. p. 43-55.

461 12. Leon DA. Trends in European life expectancy: a salutary view. International Journal of Epidemiology. 2011 Apr;40(2):271-7.

463 13. NCD Risk Factor Collaboration. Trends in adult body-mass index in 200 countries from 1975 to 2014: a pooled analysis of 1698 population-based measurement studies with 19.2 million participants. Lancet. 2016 Apr 2;387(10026):1377-96.

466 14. Lobstein T, Leach RJ. Workpackage 7: Overweight and Obesity Report on data collection for overweight and obesity prevalence and related relative risks. 2010.

468 15. Allison DB, Fontaine KR, Manson JE, Stevens J, VanItallie TB. Annual deaths attributable to obesity in the United States. JAMA. 1999 Oct 27;282(16):1530-8.

470 16. Banegas JR, Lopez-Garcia E, Gutierrez-Fisac JL, Guallar-Castillon P, Rodriguez-Artalejo F. A simple estimate of mortality attributable to excess weight in the European Union. Eur J Clin Nutr. 2003 Feb;57(2):201-8.

For peer review only - http://bmjopen.bmj.com/site/about/guidelines.xhtml

17. A Robertson, T Lobstein, C Knai. Obesity and socio-economic groups in Europe: Evidence review and implications for action. 2007.

18. Flegal KM, Graubard BI, Williamson DF, Gail MH. Excess deaths associated with underweight, overweight, and obesity. JAMA. 2005 Apr 20;293(15):1861-7.

19. Katzmarzyk PT, Ardern CI. Overweight and obesity mortality trends in Canada, 1985-2000. Can J Public Health. 2004 Jan-Feb;95(1):16-20.

20. GBD 2017 Risk Factor Collaborators. Global, regional, and national comparative risk assessment of 84 behavioural, environmental and occupational, and metabolic risks or clusters of risks for 195 countries and territories, 1990-2017: a systematic analysis for the Global Burden of Disease Study 2017. Lancet. 2018 Nov 10;392(10159):1923-94.

21. University of California, Berkeley (USA), and Max Planck Institute for Demographic Research (Germany). [Internet]. []. Available from: http://www.mortality.org.

22. Barbieri M, Wilmoth JR, Shkolnikov VM, Glei D, Jasilionis D, Jdanov D, et al. Data Resource Profile: The Human Mortality Database (HMD). Int J Epidemiol. 2015 Oct;44(5):1549-56.

23. Cleveland WS, Loader C. Smoothing by local regression: Principles and methods. In: Murray Hill: A&T Bell Laboratorie; 1995.

24. Rockhill B, Newman B, Weinberg C. Use and misuse of population attributable fractions. American Journal of Public Health. 1998 Jan;88(1):15-9.

25. Preston SH, Heuveline P, Guillot M. Demography. Malden, MA: Blackwell; 2001.

26. Census data [Internet].; 2011 []. Available from: https://ec.europa.eu/CensusHub2/query.do?step=selectHyperCube&qhc=false.

27. Flegal KM, Panagiotou OA, Graubard BI. Estimating population attributable fractions to quantify the health burden of obesity. Annals of Epidemiology. 2015 Mar;25(3):201-7.

For peer review only - http://bmjopen.bmj.com/site/about/guidelines.xhtml

497 28. Mehta NK, Chang VW. Secular declines in the association between obesity and mortality
498 in the United States. Population and Development Review. 2011;37(3):435-51.

499 29. Yu Y. Reexamining the declining effect of age on mortality differentials associated with
500 excess body mass: evidence of cohort distortions in the United States. American Journal of
501 Public Health. 2012 May;102(5):915-22.

502 30. Yu Y. The Changing Body Mass-Mortality Association in the United States: Evidence of
503 Sex-Specific Cohort Trends from Three National Health and Nutrition Examination Surveys.
504 Biodemography and Social Biology. 2016;62(2):143-63.

505 31. Vidra N, Bijlsma MJ, Janssen F. Impact of Different Estimation Methods on Obesity-
506 Attributable Mortality Levels and Trends: The Case of The Netherlands. Int J Environ Res
507 Public Health. 2018 Sep 29;15(10):10.3390/ijerph15102146.

508 32. Trias-Llimós S, Kunst AE, Jasilionis D, Janssen F. The contribution of alcohol to the East-
509 West life expectancy gap in Europe from 1990 onward. International Journal of
510 Epidemiology. 2017;47(3):731-9.

511 33. Doak CM, Wijnhoven TM, Schokker DF, Visscher TL, Seidell JC. Age standardization in
512 mapping adult overweight and obesity trends in the WHO European Region. Obesity
513 Reviews. 2012 Feb;13(2):174-91.

514 34. Cutler DM, Glaeser EL, Shapiro JM. Why Have Americans Become More Obese? The
515 Journal of Economic Perspectives. 2003;17(1):93-118.

516 35. Obesity update 2012 [Internet].; 2012 []. Available from:
517 http://www.oecd.org/els/health-systems/49716427.pdf.

518 36. Malik VS, Willett WC, Hu FB. Global obesity: trends, risk factors and policy implications.
519 Nature reviews Endocrinology. 2013 Jan;9(1):13-27.

520 37. Silventoinen K, Sans S, Tolonen H, Monterde D, Kuulasmaa K, Kesteloot H, et al. Trends in
521 obesity and energy supply in the WHO MONICA Project. International Journal of Obesity and
522 Related Metabolic Disorders. 2004 May;28(5):710-8.

For peer review only

38. Zatonski WA, Bhala N. Changing trends of diseases in Eastern Europe: closing the gap. Public Health. 2012 Mar;126(3):248-52.

39. Bray GA, Bouchard C. Handbook of Obesity: Etiology and Pathophysiology. second ed. Boca Raton: CRC Press Taylor & Francis Group; 2003.

40. Monteiro CA, Conde WL, Lu B, Popkin BM. Obesity and inequities in health in the developing world. International Journal of Obesity. 2004 Sep;28(9):1181-6.

41. Wilkins E, Wilson L, Wickramasinghe K, Bhatnagar P, Leal J, Luengo-Fernandez R, et al. European Cardiovascular Disease Statistics 2017. Brussels: European Heart Network; 2017.

42. Robertson A, Lobstein T, Knai C. Obesity and socio-economic groups in Europe: Evidence review and implications for action. Brussels: European Commission. 2007.

43. Fagerli RA, Wandel M. Gender differences in opinions and practices with regard to a "healthy diet". Appetite. 1999 Apr;32(2):171-90.

44. Psaltopoulou T, Hatzis G, Papageorgiou N, Androulakis E, Briasoulis A, Tousoulis D. Socioeconomic status and risk factors for cardiovascular disease: Impact of dietary mediators. Hellenic Journal of Cardiology. 2017 Jan - Feb;58(1):32-42.

45. WHO. Obesity and inequities. Guidance for addressing inequities in overweight and obesity. Copenhagen: World Health Organization, Regional Office for Europe; 2014.

For peer review only

For peer review only - http://bmjopen.bmj.com/site/about/guidelines.xhtml

Figure legends

Figure 1: Age-standardised obesity-attributable mortality fractions in 26 European countries* (by 5 regions) and USA, 1975-2014, 18-100 years

* Countries within the same region are presented with the same colour

Central Europe: Czech Republic, Hungary, Poland, Slovakia

Eastern Europe: Belarus, Estonia, Ukraine, Latvia, Lithuania, Russian Federation

Northern Europe: Denmark, Finland, Iceland, Norway, Sweden

Southern Europe: Italy, Portugal, Spain

Western Europe: Austria, Belgium, France, Ireland, Luxembourg, Netherlands, Switzerland, United Kingdom

USA: United States of America

Figure 2: Potential gains in life expectancy at birth (PGLE) if obesity-attributable mortality was eliminated, in 26 European countries* (differentiating Western and Central Eastern Europe) and the USA, 1975-2012, 18-100 years

* Countries within the same region are presented with the same colour

Central Eastern Europe: Belarus, Czech Republic, Estonia, Hungary, Latvia, Lithuania, Poland, Russian Federation, Slovakia, Ukraine

Western Europe: Austria, Belgium, Denmark, Finland, France, Iceland, Ireland, Italy, Luxembourg, Netherlands, Norway, Portugal, Spain, Sweden, Switzerland, United Kingdom

USA: United States of America

Figure 3: Average annual increase (%) in potential gains in life expectancy due to obesity in 26 European countries and the USA between 1975-2012, in men

Figure 4: Average annual increase (%) in potential gains in life expectancy due to obesity in 26 European countries and the USA between 1975-2012, in women

For peer review only

For peer review only - http://bmjopen.bmj.com/site/about/guidelines.xhtml

For peer review only

For peer review only - http://bmjopen.bmj.com/site/about/guidelines.xhtml

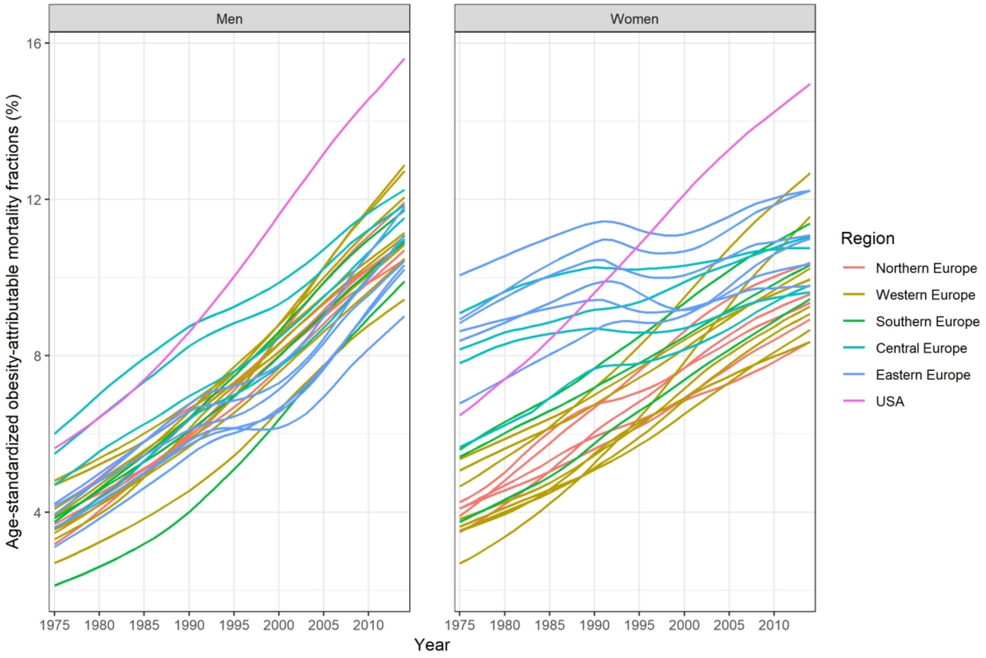

Figure 1: Age-standardised obesity-attributable mortality fractions in 26 European countries* (by 5 regions) and USA, 1975-2014, 18-100 years
* Countries within the same region are presented with the same colour
Central Europe: Czech Republic, Hungary, Poland, Slovakia
Eastern Europe: Belarus, Estonia, Ukraine, Latvia, Lithuania, Russian Federation
Northern Europe: Denmark, Finland, Iceland, Norway, Sweden
Southern Europe: Italy, Portugal, Spain
Western Europe: Austria, Belgium, France, Ireland, Luxembourg, Netherlands, Switzerland, United Kingdom
USA: United States of America

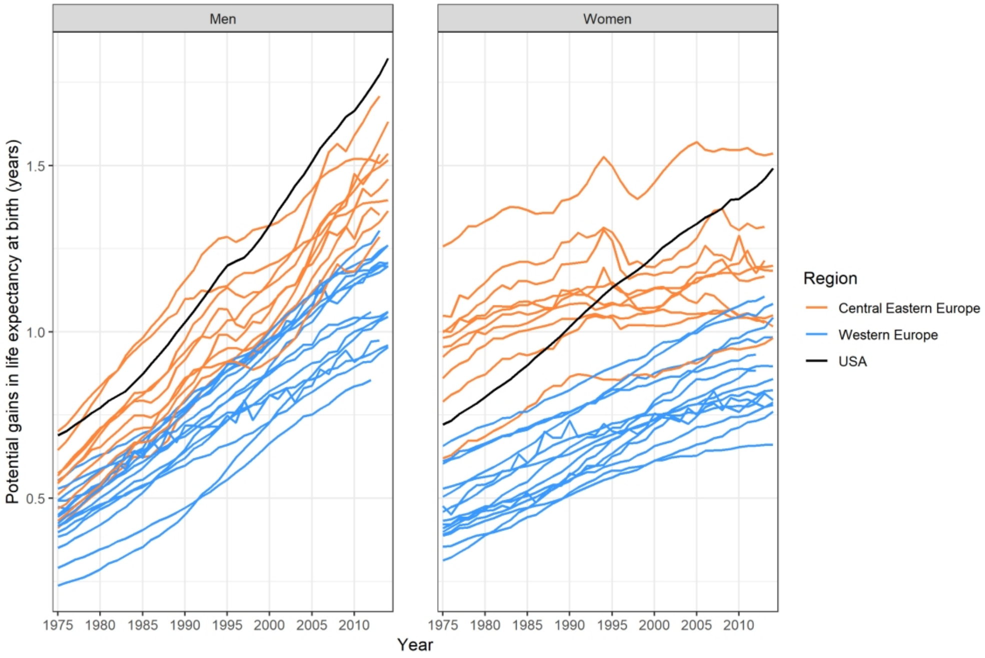

Figure 2: Potential gains in life expectancy at birth (PGLE) if obesity-attributable mortality was eliminated, in 26 European countries* (differentiating Western and Central Eastern Europe) and the USA, 1975-2012
* Countries within the same region are presented with the same colour
Central Eastern Europe: Belarus, Czech Republic, Estonia, Hungary, Latvia, Lithuania, Poland, Russian Federation, Slovakia, Ukraine
Western Europe: Austria, Belgium, Denmark, Finland, France, Iceland, Ireland, Italy, Luxembourg, Netherlands, Norway, Portugal, Spain, Sweden, Switzerland, United Kingdom
USA: United States of America

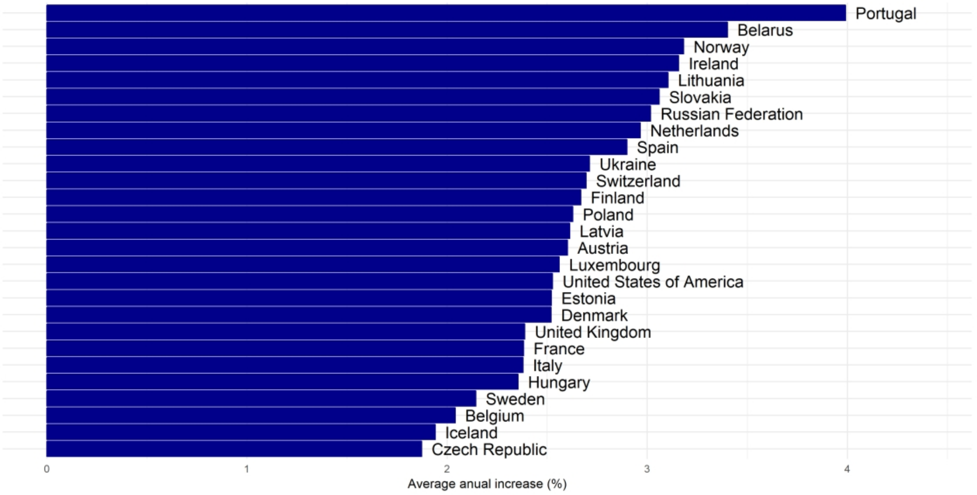

Figure 3: Average annual increase (%) in potential gains in life expectancy due to obesity in 26 European countries and the USA between 1975-2012, in men

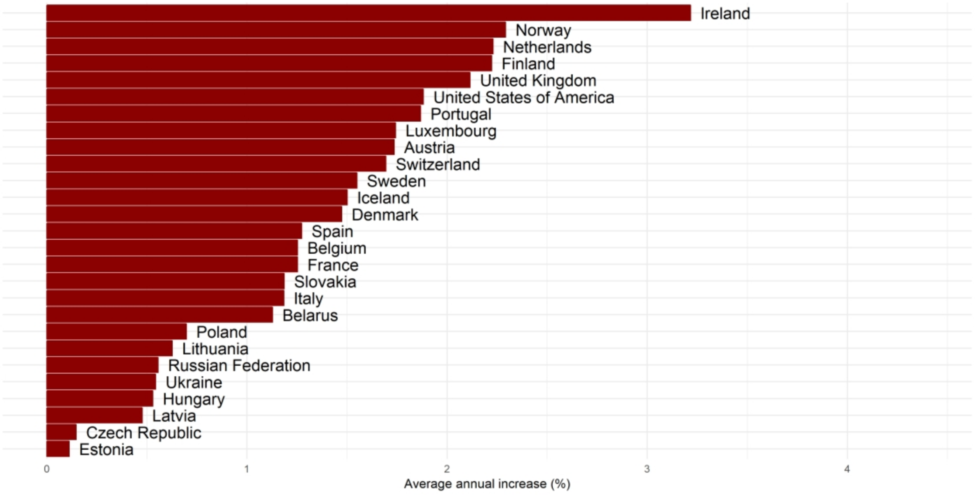

Figure 4: Average annual increase (%) in potential gains in life expectancy due to obesity in 26 European countries and the USA between 1975-2012, in women

Impact of obesity on life expectancy among different European countries, 1975-2012

Nikoletta Vidra[1], Sergi Trias-Llimós[1], Fanny Janssen[1,2]

[1] Population Research Centre, Faculty of Spatial Sciences, University of Groningen, Landleven 1, 9747 AD Groningen, The Netherlands.

[2] Netherlands Interdisciplinary Demographic Institute, Lange Houtstraat 19, 2511 CV The Hague, The Netherlands

Supplementary material

Table S1: Age-and sex-specific RRs of dying from obesity from the meta-review from Lobstein et al. (2010)

**Age**	**RR***	
	Men	***Women***
**<50**	1.55	1.5
**50-59**	1.539	1.49
**60-69**	1.5225	1.475
**70+**	1.495	1.45

*Reference group for the RRs: normal weight (18.5≥BMI≤24.9 kg/m^2)

For peer review only

Table S2: Potential gains in life expectancy at birth (PGLE) if obesity-attributable mortality was eliminated, in 26 European countries (differentiating Western and Central Eastern Europe) and the USA, in 1975 and 2012, 18-100 years

	**PGLE 1975**		**PGLE 2012**	
**Country**	***Men***	***Women***	***Men***	***Women***
Central Eastern Europe (CEE)				
Belarus	0.41	0.79	1.41	1.19
Czech Republic	0.70	0.98	1.39	1.03
Estonia	0.55	1.00	1.37	1.04
Hungary	0.64	0.86	1.52	1.04
Latvia	0.58	1.00	1.48	1.18
Lithuania	0.54	1.05	1.67	1.31
Poland	0.57	0.93	1.48	1.19
Russian Federation	0.51	1.26	1.53	1.54
Slovakia	0.43	0.62	1.31	0.96
Ukraine	0.47	0.95	1.25	1.16
Average CEE	0.54	0.94	1.44	1.16
Western Europe				
Austria	0.40	0.39	1.03	0.73
Belgium	0.55	0.61	1.17	0.97
Denmark	0.42	0.46	1.04	0.79
France	0.49	0.53	1.18	0.84
Finland	0.45	0.40	1.19	0.90
Ireland	0.38	0.31	1.21	1.01
Iceland	0.49	0.48	0.97	0.80
Italy	0.44	0.60	1.06	0.93
Luxembourg	0.47	0.42	1.19	0.79
Netherlands	0.29	0.39	0.86	0.88
Norway	0.34	0.39	1.07	0.91
Portugal	0.24	0.41	1.01	0.81
Spain	0.42	0.66	1.22	1.05
Sweden	0.42	0.43	0.91	0.76
Switzerland	0.35	0.35	0.93	0.66
United Kingdom	0.53	0.50	1.27	1.09
**Average Western Europe**	0.41	0.48	1.08	0.86
USA	0.69	0.72	1.73	1.44
**Average European countries**	0.46	0.64	1.22	0.98
**Average all countries**	0.47	0.64	1.23	1.00

For peer review only

Figure S1: Age-standardised obesity-attributable mortality fractions in 26 European countries*, grouped by 5 regions and USA, 1975-2014, 18-100 years

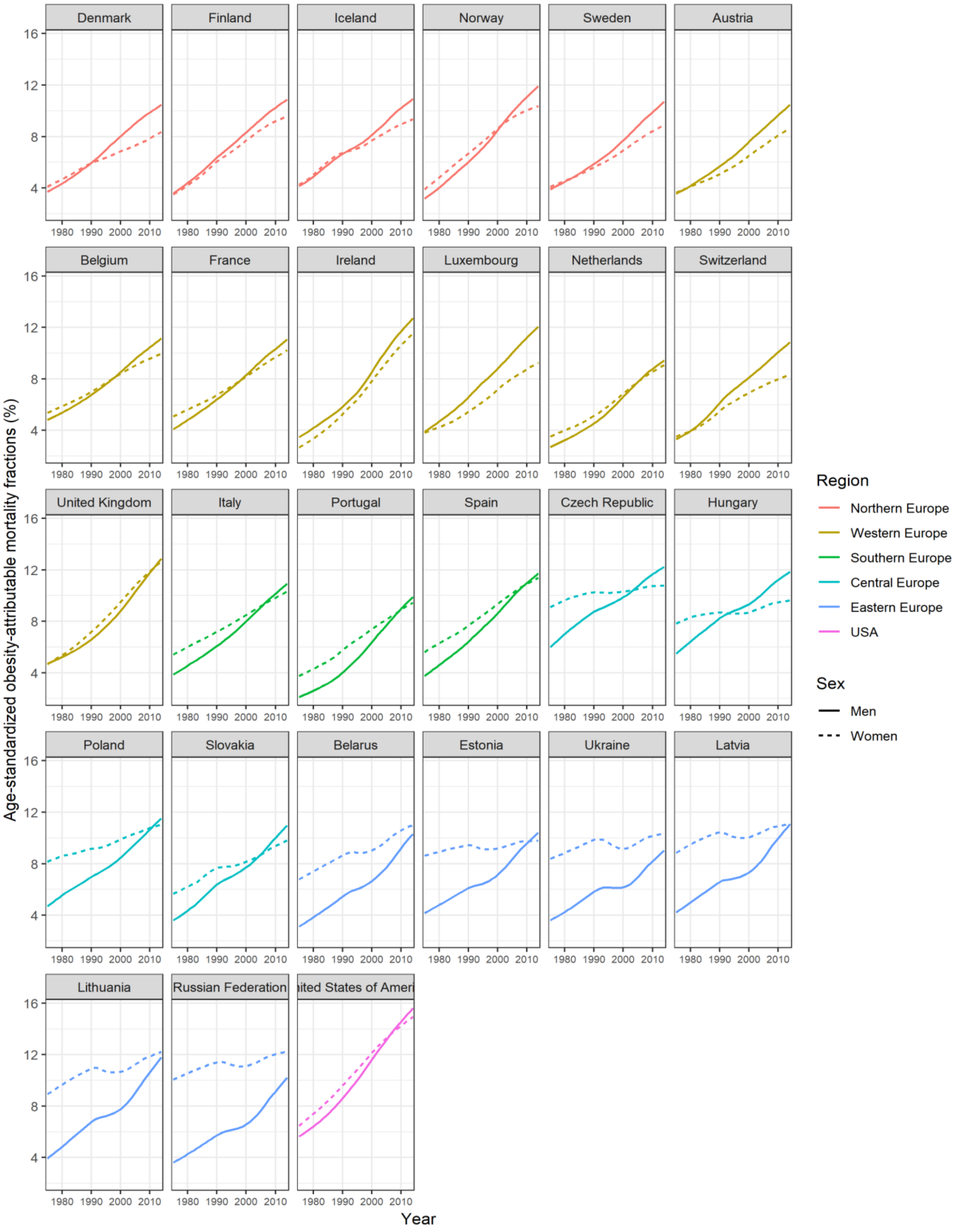

* Countries within the same region are presented with the same colour

Figure S2: Potential gains in life expectancy at birth (PGLE) if obesity-attributable mortality was eliminated, in 26 European countries*, grouped by 5 regions and USA, 1975-2012, 18-100 years

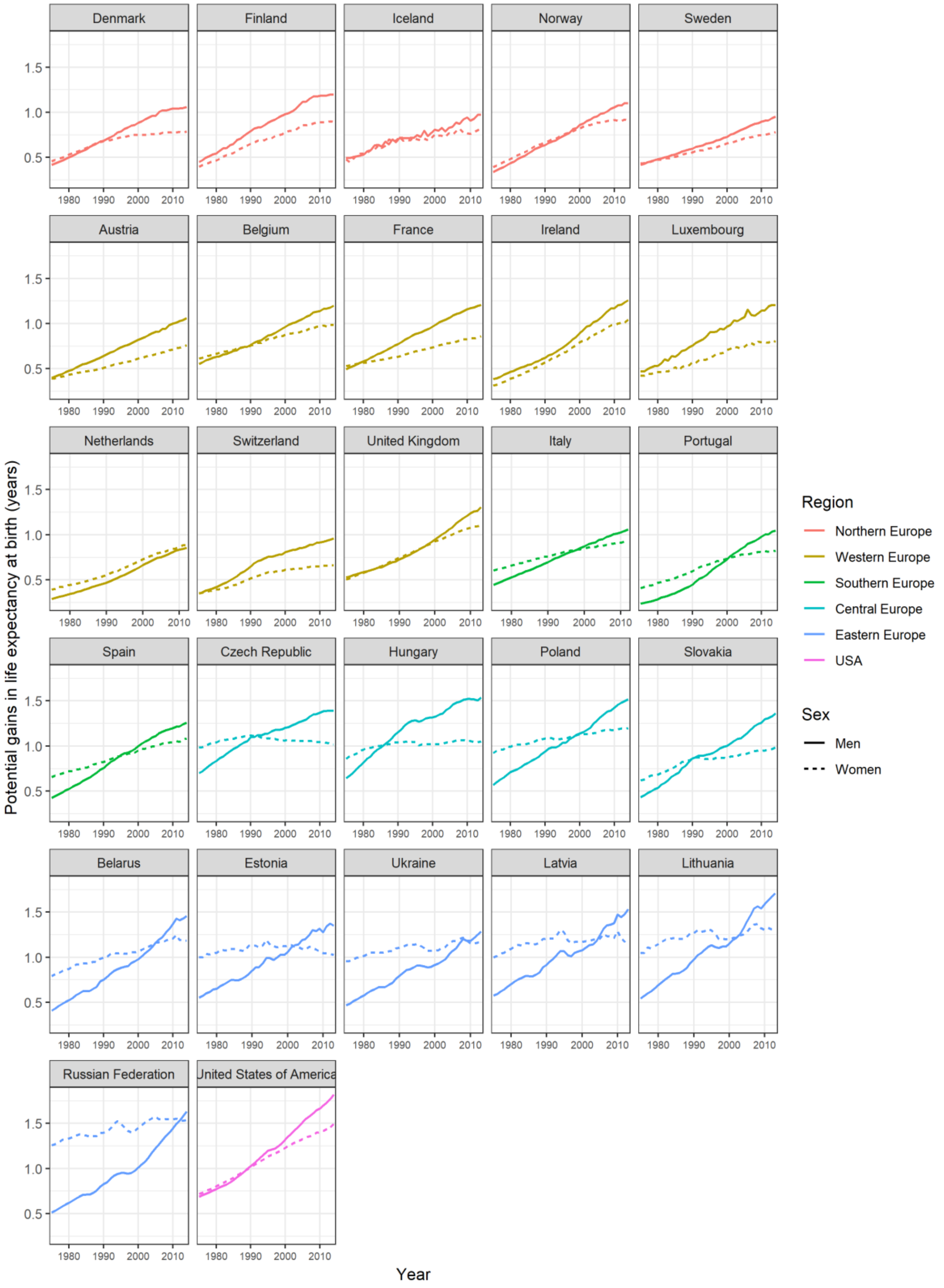

* Countries within the same region are presented with the same colour

Table S3: Age-standardised obesity prevalence and 95% confidence intervals, in 26 European countries (differentiating Western and Central Eastern Europe) and USA, 18-100 years in 2012.

	Age-standardised (stand.) obesity prevalence (%)			
**Country**	**Age-stand. Men**	**95% confidence intervals**	**Age-stand. Women**	**95% confidence intervals**
Central Eastern Europe				
Belarus	20.4	13.6; 28.5	25.4	17.7; 34.1
Czech Republic	25.6	18.9; 33.4	25.2	18.3; 33.0
Estonia	21.0	16.0; 26.8	22.7	17.4; 28.8
Hungary	24.6	17.9;32.0	22.1	15.4; 29.8
Latvia	22.2	15.2; 30.2	25.8	18.4; 34.5
Lithuania	23.8	16.9; 31.8	28.7	21.2; 37.1
Poland	23.6	18.0; 29.7	25.7	19.5; 32.5
Russian Federation	20.4	14.8; 26.8	29.1	22.6; 36.2
Slovakia	22.2	15.7; 29.6	22.2	15.8; 29.6
Ukraine	17.7	11.2; 25.7	23.9	16.3; 32.5
Western Europe				
Austria	21.1	14.7; 28.2	19.1	13.4; 25.5
Belgium	22.9	17.3; 29.1	22.7	17.1; 28.9
Denmark	21.3	15.5; 27.9	18.4	13.1; 24.5
France	22.6	16.3; 29.6	23.1	16.9; 29.9
Finland	22.3	17.1; 28.2	21.7	16.7; 27.3
Ireland	26.2	19.4; 33.8	26.0	19.5; 33.2
Iceland	22.3	15.6; 29.8	21.0	14.7; 28.3
Italy	22.3	17.0; 28.1	23.5	18.1; 29.6
Luxembourg	24.9	17.6; 32.9	20.7	14.3; 28.0
Netherlands	18.9	13.8; 24.5	20.3	15.3; 25.8
Norway	24.5	18.5; 31.2	23.7	18.0; 30.2
Portugal	19.6	13.8; 26.4	21.1	15.0; 28.1
Spain	24.2	18.5; 30.5	26.4	20.3; 32.8
Sweden	21.6	16.3; 27.4	19.8	14.7; 25.6
Switzerland	22.0	16.3; 28.3	18.6	13.1; 24.8
United Kingdom	26.5	22.3; 31.2	29.1	24.8; 33.6
USA	33.4	27.5; 39.5	35.5	29.7; 41.5

For peer review only - http://bmjopen.bmj.com/site/about/guidelines.xhtml

Table S4: Potential gains in life expectancy at age 50 (PGLE) if obesity-attributable mortality was eliminated, own estimates and those by Preston et al.2011, in the same countries studied, in 2006

Country	PGLE e50 2006, own estimates	PGLE e50 2006, Preston's estimates	Difference
Men			
Austria	0.81	1.00	-0.19
Belgium	0.95	0.98	-0.03
Czech Republic	1.14	1.34	-0.20
Denmark	0.88	0.82	0.06
France	0.94	0.99	-0.05
Italy	0.88	0.90	-0.02
Netherlands	0.69	0.73	-0.04
Poland	1.14	1.37	-0.23
Spain	1.02	1.15	-0.13
Sweden	0.75	0.72	0.03
Switzerland	0.77	0.79	-0.02
United Kingdom	0.99	1.34	-0.35
USA	1.29	1.85	-0.56
Women			
Austria	0.62	0.71	-0.09
Belgium	0.86	0.73	0.13
Czech Republic	0.87	1.01	-0.14
Denmark	0.71	0.62	0.09
France	0.72	0.52	0.2
Italy	0.84	0.57	0.27
Netherlands	0.76	0.69	0.07
Poland	1.08	1.19	-0.11
Spain	0.95	0.87	0.08
Sweden	0.67	0.63	0.04
Switzerland	0.59	0.50	0.09
United Kingdom	0.94	1.23	-0.29
USA	1.18	1.28	-0.10

For peer review only - http://bmjopen.bmj.com/site/about/guidelines.xhtml

Figure S3: Age-standardised obesity prevalence in 26 European countries*, grouped by 5 regions and USA, 1975-2012, 18-100 years

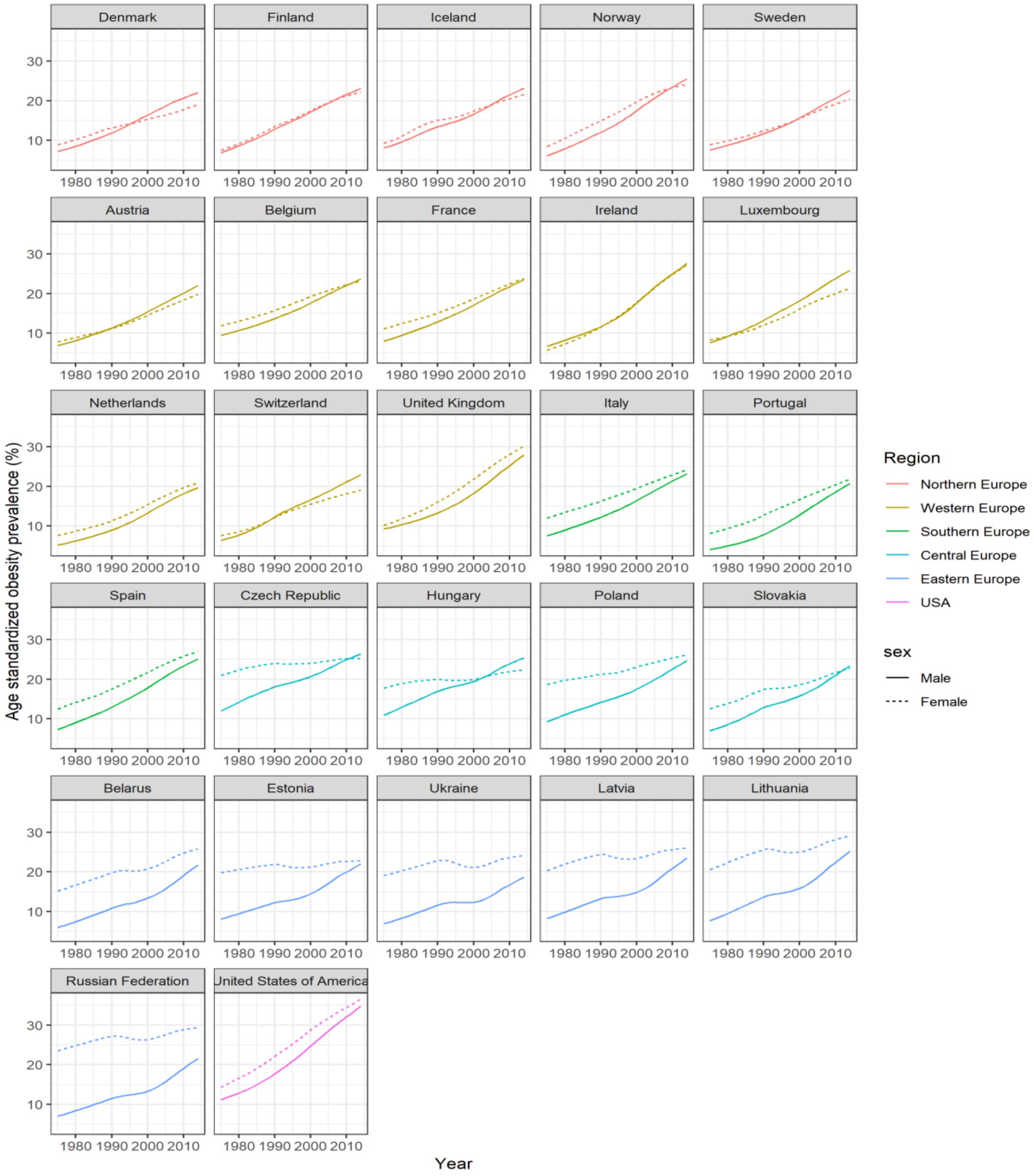

* Countries within the same region are presented with the same colour
